# S100A5 Attenuates Efficiency of Anti‐PD‐L1/PD‐1 Immunotherapy by Inhibiting CD8^+^ T Cell‐Mediated Anti‐Cancer Immunity in Bladder Carcinoma

**DOI:** 10.1002/advs.202300110

**Published:** 2023-07-06

**Authors:** Huihuang Li, Jinbo Chen, Zhenghao Li, Minfeng Chen, Zhenyu Ou, Miao Mo, Ruizhe Wang, Shiyu Tong, Peihua Liu, Zhiyong Cai, Chunyu Zhang, Zhi Liu, Dingshan Deng, Jinhui Liu, Chunliang Cheng, Jiao Hu, Xiongbing Zu

**Affiliations:** ^1^ Department of Urology Xiangya Hospital Central South University Changsha 410008 China; ^2^ National Clinical Research Center for Geriatric Disorders Xiangya Hospital Central South University Changsha 410008 China; ^3^ Hunan Provincial Key Laboratory of Hepatobiliary Disease Research and Division of Hepato‐Biliary‐Pancreatic Surgery Department of General Surgery The Second Xiangya Hospital Central South University Changsha 410011 China

**Keywords:** bladder carcinoma, effector immune cells, immunotherapy, S100 family, tumor microenvironment

## Abstract

Although immune checkpoint blockade (ICB) therapies have been approved for bladder cancer (BLCA), only a minority of patients respond to these therapies, and there is an urgent need to explore combined therapies. Systematic multi‐omics analysis identified S100A5 as a novel immunosuppressive target for BLCA. The expression of S100A5 in malignant cells inhibited CD8^+^ T cell recruitment by decreasing pro‐inflammatory chemokine secretion. Furthermore, S100A5 attenuated effector T cell killing of cancer cells by inhibiting CD8^+^ T cell proliferation and cytotoxicity. In addition, S100A5 acted as an oncogene, thereby promoting tumor proliferation and invasion. Targeting S100A5 synergized with the efficacy of anti‐PD‐1 treatment by enhancing infiltration and cytotoxicity of CD8^+^ T cells in vivo. Clinically, there was a spatially exclusive relationship between S100A5^+^ tumor cells and CD8^+^ T cells in tissue microarrays. Moreover, S100A5 negatively correlated with immunotherapy efficacy in our real‐world and several public immunotherapy cohorts. In summary, S100A5 shapes a non‐inflamed tumor microenvironment in BLCA by inhibiting the secretion of pro‐inflammatory chemokines and the recruitment and cytotoxicity of CD8^+^ T cells. Targeting S100A5 converts cold tumors into hot tumors, thus enhancing the efficacy of ICB therapy in BLCA.

## Graphical abstract

Systematic multi‐omics analysis identifies S100A5 as a novel immunosuppressive target for bladder cancer, which shapes a non‐inflamed tumor microenvironment in bladder cancer by inhibiting the secretion of pro‐inflammatory chemokines and the recruitment and cytotoxicity of CD8^+^ T cells. Targeting S100A5 converts cold tumors into hot tumors, thus enhancing the efficacy of immunotherapy in bladder cancer.

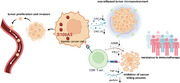

## 1 Introduction

Bladder carcinoma (BLCA) is one of the most prevalent carcinomas worldwide, with 573 278 new cases and 212 536 new deaths reported in 2020.^[^ ^1^ ^]^ Despite radical cystectomy and platinum‐based chemotherapy, advanced BLCA, including locally advanced and metastatic BLCA, is commonly regarded as an incurable disease with extremely poor prognosis.^[^ ^2^, ^3^ ^]^ Owing to their high tumor mutation burden (TMB), immune checkpoint inhibitors (ICIs) have gained increasing attention for the treatment of advanced BLCA.^[^ ^4^, ^5^ ^]^ Since 2016, five ICIs, including atezolizumab, durvalumab, avelumab, pembrolizumab, and nivolumab have been approved by the US Food and Drug Administration (FDA) for the treatment of advanced BLCA.^[^ ^2^ ^]^ However, only a minority of patients respond to ICIs treatment, suggesting an urgent need to identify novel biomarkers that can not only accurately predict ICIs response but also have the potential to be promising immunotherapy targets.^[^ ^5^, ^6^ ^]^

Numerous factors affect ICI efficiency. Immune profiles of the tumor microenvironment (TME) are vital elements that determine how a response occurs.^[^ ^7^ ^]^ The TME is composed of tumor cells, fibroblasts, vascular endothelial cells, immune cells, extracellular matrix, and extracellular soluble molecules.^[^ ^8^ ^]^ Based on the presence or absence of T cells in the tumor parenchyma, the TME can generally be divided into two profiles: non‐inflamed tumors (without T cell infiltration into the tumor parenchyma, including immune‐desert and immune‐excluded phenotypes) and inflamed tumors (with T cell infiltration into the tumor parenchyma, including immune‐inflamed phenotype).^[^ ^9^ ^]^ Theoretically, ICIs, including anti‐programmed death 1 (PD‐1) and anti‐programmed death‐ligand 1 (PD‐L1), exhibit no therapeutic effects without T cell immunity. Consistent with this, inflamed tumors have higher response rates to immunotherapy, including ICIs.^[^ ^10^ ^]^ In addition, the combination of both anti‐PD‐1 and cytotoxic T lymphocyte antigen 4 (CTLA‐4) has shown much higher effectiveness because CTLA‐4 increases the production of tumor‐specific T cells by moving the checkpoint for T cell proliferation and priming.^[^ ^11^ ^]^ Inspired by this, we are committed to identifying a biomarker that can not only predict the infiltration of tumor‐infiltrating immune cells (TIICs) for diagnosis, but also develop new combined anti‐PD‐1/PD‐L1 treatment strategies for higher therapeutic efficacy.

Members of the S100 protein family play vital roles in tumor invasion and immune evasion by acting as Ca^2+^ mediators and extracellular factors.^[^ ^12^ ^]^ In breast cancer, tumor cells secrete S100A7 to recruit tumor‐associated macrophages (TAMs).^[^ ^13^ ^]^ In addition, S100A8 and S100A9 can recruit myeloid‐derived suppressor cells (MDSCs) to maintain an immunosuppressive state in the TME of breast cancer.^[^ ^12^ ^]^ In melanoma, S100A4 can increase the release of inflammatory cytokines and promote tumor immune responses.^[^ ^14^ ^]^ S100A5 has been reported to play a vital role in the recurrence of the World Health Organization (WHO) grade I meningiomas.^[^ ^15^ ^]^ In addition, S100A5 was significantly upregulated in the BLCA.^[^ ^16^ ^]^ However, their comprehensive roles in TME and immunotherapy are unclear, especially in BLCA. Therefore, in this study, we focused on S100A5 by comprehensively analyzing the expression patterns and immunological roles of multiple S100 family proteins in BLCA. Pan‐cancer analyses revealed that the immunosuppressive role of S100A5 in the TME was most evident in BLCA. Based on these findings, we validated the oncogenic and immunosuppressive roles of S100A5 in BCLA both in vitro and in vivo. Moreover, the value of predicting immunotherapy response was revealed in our real‐world and multiple immunotherapy cohorts.

## 2 Results

### 2.1 Focusing S100A5 in BLCA through Comprehensive Analyses

S100 family proteins including S100A1‐16, S100B, and S100P were summarized based on the review of Bresnick et al.^[^ ^12^ ^]^ According to the principles of normalization cancer immunotherapy,^[^ ^49^ ^]^ molecular targets should meet two conditions for potential immunotherapy: specific expression in carcinoma cells and inhibition of TIICs infiltration. Therefore, we first analyzed the expression patterns of these proteins in the TCGA‐BLCA and Xiangya BLCA cohorts and found that S100A5, S100A7, S100A11, S100A14‐16, and S100B were significantly higher in carcinoma tissues than normal tissues in both cohorts (Figure S1A,B, Supporting Information). Then, 28 immune cells were identified using the single‐sample gene set enrichment analysis (ssGSEA) algorithm and systematically correlated with S100 proteins. As shown in Figure S1C,D (Supporting Information), only S100A5 and S100A6 were significantly negatively correlated with TIICs infiltration in both cohorts. Combined with the expression patterns, we focused on S100A5 in this study.

We then performed pan‐cancer analyses of S100A5 in 33 types of carcinomas. In most carcinomas, S100A5 was expressed at significantly higher levels in tumor tissues than in normal tissues, including bladder cancer tissues (Figure S2A,B, Supporting Information). In addition, we found that S100A5 was significantly higher in the tumor tissues in our TMA (Figure S2C, Supporting Information). S100A5 was also expressed in several normal tissues (Figure S2D, Supporting Information). Next, we explored the immunological role of S100A5 in multiple carcinomas. As shown in Figure S3A (Supporting Information), the negative correlation between S100A5 and immunomodulators was most evident in BLCA. We selected four important immune checkpoints, including PD‐L1, CTLA‐4, PD‐1, and lymphocyte activation gene‐3 (LAG‐3), and found that S100A5 had the most exclusive association with these immune checkpoints in BLCA (Figure S3B–E, Supporting Information). Furthermore, S100A5 was significantly negatively correlated with most TIICs in the BLCA (Figure S3F, Supporting Information). However, there was no significant negative correlation between S100A5 and TIICs in other carcinomas, such as lung adenocarcinoma (LUAD), sarcoma (SARC), breast invasive carcinoma (BRCA), and colon adenocarcinoma (COAD) (Figure S3F, Supporting Information). In summary, we found that only S100A5 shaped a non‐inflamed TME specifically in BLCA, by correlating multiple S100 proteins with multiple cancer types.

### 2.2 S100A5 Shapes a Non‐Inflamed TME in BLCA

**Figure 1:**
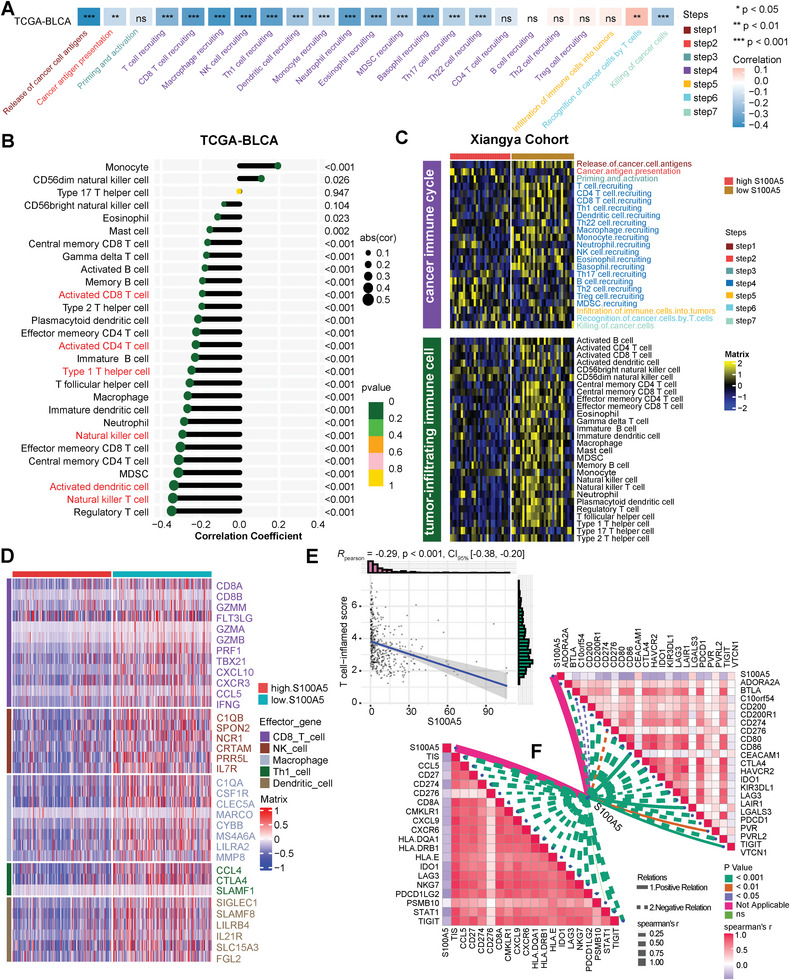
Correlations between S100A5 and tumor microenvironment (TME) characteristics in TCGA‐BLCA and Xiangya cohort. A) Correlation between S100A5 and cancer‐immunity cycles in TCGA‐BLCA cohort. Different colors represent different cycles; Positive correlation is marked in red, while negative correlation is marked in blue. B) Correlation between S100A5 and tumor‐infiltrating immune cells (TIICs) using ssGSEA algorithm in TCGA‐BLCA cohort. C) Validation the role of S100A5 with cancer‐immunity cycles and TIICs using ssGSEA algorithm in Xiangya cohort. Yellow represents higher infiltration cells, while blue represents lower infiltration cells. D) Different expression patterns of effector genes of CD8^+^ T cells, dendritic cells (DCs), macrophage cells, natural killer (NK) cells and type 1 T helper (Th1) cells between high and low S100A5 groups; Red represents higher expressed genes, while blue represents lower expressed genes. E) Correlation between S100A5 and pan‐cancer T cell‐inflamed score (TIS). F) Correlation between S100A5 and TIS related genes (bottom left) and immune checkpoint inhibitor (ICI) genes (upper right); Positive correlation was marked in red, while negative correlation was marked in blue. ns, not statistically significant. *p < 0.05; **p < 0.01; ***p < 0.001.

**Figure 2:**
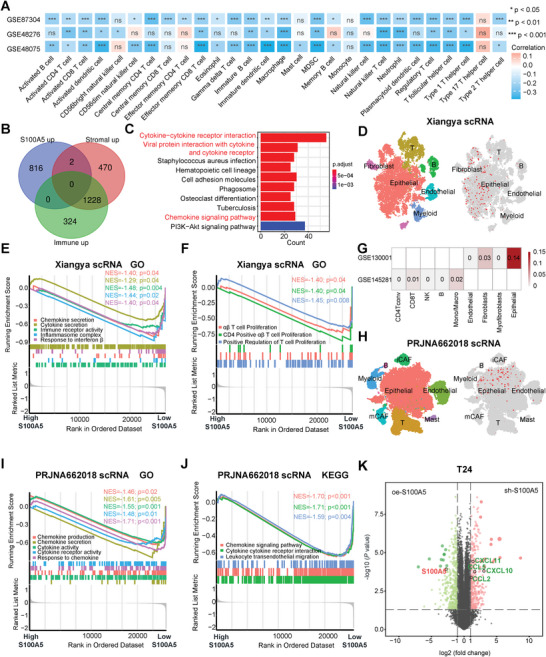
S100A5 specifically expressed on tumor cells and inhibited pro‐inflammatory cytokine and chemokines secretion. A) Correlation between S100A5 and tumor‐infiltrating immune cells (TIICs) using the ssGSEA algorithm in the GSE87304, GSE48276, and GSE48075 cohorts. B) Venn Diagram showing common differentially expressed genes (DEGs) between the high S100A5, stromal score and immune score groups. C) KEGG enrichment results for common DEGs. D) *t*SNE plot of all single cells and S100A5 expression patterns in Xiangya scRNA‐seq. E,F) GSEA shows the enrichment of cytokine and chemokine secretion related pathways E) and T cell infiltration‐related pathways F) between different S100A5 expression groups in malignant epithelial cells in the Xiangya scRNA‐seq cohort. NES: normalized enrichment score. G) S100A5 expression levels in the TME (GSE130001) and blood cells (GSE145281). H) *t*SNE plot of all single cells and S100A5 expression patterns in the PRJNA662018 scRNA cohort. iCAF, inflammatory cancer‐associated fibroblasts; mCAF, myo‐cancer‐associated fibroblasts. I,J) GSEA shows GO I) and KEGG J) enrichment of cytokine and chemokine secretion‐related pathways between different S100A5 expression groups in malignant epithelial cells in the PRJNA662018 scRNA cohort. K) Volcano plot showing the major DEGs between the knockdown and overexpression S100A5 groups in T24 cell. ns, not statistically significant. *p < 0.05; **p < 0.01; ***p < 0.001.

The cancer‐immune cycle includes the following steps: first, the death of cancer cells releases cancer antigens; then, antigen‐presenting cells (APCs), such as dendritic cells (DCs), capture and present antigens to T cells, resulting in the activation of effector T cells. Effector T cells traffic to the tumor sites and infiltrate the tumor bed. Finally, effector T cells recognize and kill tumor cells and release more antigens.^[^ ^50^, ^51^ ^]^ Based on the bulk RNA‐seq data of TCGA‐BLCA, we downloaded the levels of these steps from the tracking tumor immunophenotype (TIP) (http://biocc.hrbmu.edu.cn/TIP/). As shown in **Figure** **1**A; Table S1 (Supporting Information), most of these steps were significantly negatively correlated with S100A5, indicating that S100A5 may inhibit the cancer‐immune cycle and shape a non‐inflamed TME in BLCA. Furthermore, we directly evaluated the infiltration of 28 immune cell types using the ssGSEA algorithm (Figure 1B; Table S1, Supporting Information) and found significant negative correlations between S100A5 expression and most immune cells, including activated CD8^+^ T cells, type 1 T helper (Th1) cells, natural killer (NK) cells, activated CD4^+^ T cells, activated DCs, and NK T cells. To eliminate the influence of this algorithm, six other algorithms (CIBERSORT, EPIC, mMCP‐counter, quanTIseq, TIMER, and xCell) were used to calculate immune cell infiltration. As expected, S100A5 expression was significantly and negatively correlated with most cytotoxic T lymphocytes (CTLs) and NK cells, regardless of the algorithm (Table S1, Supporting Information). Importantly, the negative correlation between S100A5, the cancer‐immune cycle, and TIICs was validated in the Xiangya cohort (Figure 1C; Table S2, Supporting Information), indicating a robust predictive value of S100A5 for TIICs in BLCA. In addition to TIICs, S100A5 expression negatively correlated with the effector genes of CD8^+^ T cells, DCs, macrophages, NK cells, and Th1 cells (Figure 1D; Table S3, Supporting Information). Next, we calculated the pan‐cancer T cell‐inflamed score (TIS) and found that the S100A5 was significantly negatively correlated (R = −0.29, *p* < 0.001; Figure 1E). Furthermore, we summarized the TIS genes and found that S100A5 was significantly negatively correlated with almost all TIS genes (Figure 1F, bottom left; Table S3, Supporting Information). Finally, we found that S100A5 expression was significantly negatively correlated with most ICI genes, such as PD‐1, PD‐L1, LAG‐3, and CTLA‐4, which were reported to be lower in the non‐inflamed TME (Figure 1F, upper right; Table S3, Supporting Information).^[^ ^52^ ^]^ Numerous public databases were used to validate the results. As showed in **Figure** **2**A, S100A5 was significantly negatively correlated with immune cell infiltration in GSE87304, GSE48276, and GSE48075. Similar results were found for GSE120736, GSE31684, GSE32894, GSE69795, and E‐MTAB‐1803 (Figure S4–S8, Supporting Information). In summary, using bulk RNA‐seq data, we found that S100A5 shaped a non‐inflamed TME in BLCA.

### 2.3 Validating the Role of S100A5 in TME in Terms of Molecular Subtypes

Bladder cancer can be divided into molecular subtypes based on transcriptome profiling, which can precisely classify patients according to their prognosis and therapeutic options.^[^ ^35^, ^40^, ^42^ ^]^ We found that the low S100A5 group patients were more likely to be the basal subtype, while those belonging to the high S1005A group could be the luminal subtype based on seven molecular classification systems in both TCGA‐BLCA and Xiangya cohorts (Figure S9A and Table S4, Supporting Information). In addition, the area under the ROC curves (AUCs) were more than 0.85 in the TCGA‐BLCA cohort (Figure S9B and Table S4, Supporting Information) and reached 0.90 in the Xiangya cohort (Figure S9C and Table S4, Supporting Information), indicating a robust high predictive value of S100A5 for molecular subtypes. Urothelial, Ta, and luminal differentiation pathways were enriched in the high S100A5 group, whereas basal differentiation pathways were enriched in the low S100A5 group (Figure S9A and Table S4, Supporting Information). It is generally considered that the basal subtypes (low S100A5 group) possess more cytotoxic lymphocytes and NK cell infiltration and could be more sensitive to immunotherapies.^[^ ^35^ ^]^ In addition, the interferon response and immune differentiation pathways were enriched in the low S100A5 group (Figure S9A and Table S4, Supporting Information). We successfully validated these results using GSE48075 and GSE48276 cohorts (Figure S9D–F, Supporting Information). In summary, we confirmed that S100A5 expression was negatively correlated with immune cell infiltration and response to immunotherapies in terms of BLCA molecular subtypes.

### 2.4 Combining Bulk RNA‐seq and scRNA‐seq Revealed that S100A5 could Inhibit Pro‐Inflammatory Cytokine and Chemokines Secretion

Dividing the TCGA‐BLCA cohort into two groups based on the median expression of S100A5, we filtered 1803 differentially expressed genes (DEGs) between the high and low S100A5 groups (|log2FC| >1 and adj.P.value <0.05) (Table S5, Supporting Information). Surprisingly, there was only one common gene between the low S100A5, stromal score, and immune score groups (Figure S10A and Table S5, Supporting Information). Moreover, there were no common genes among the high S100A5, stromal score, and immune score groups (Figure 2B; Table S5, Supporting Information). These results confirmed the exclusive role of S100A5 with stromal and immune scores in terms of DEGs. We identified 967 common genes (Figure S10B and Table S5, Supporting Information) and performed Gene Ontology (GO) and Kyoto Encyclopedia of Genes and Genomes (KEGG) analyses using these DEGs. The majority of KEGG pathways were enriched in cytokine‐ or chemokine‐related pathways (Figure 2C; Table S6, Supporting Information). Also, GO pathways were enriched in immune‐related pathways, such as T cell activation, response to interferon‐*γ*, immune receptor activity, cytokine and chemokine activity (Figure S10C–E and Table S6, Supporting Information).

These analyses were conducted using bulk RNA‐seq, the fundamental principle of which is that all genes are expressed equally in every cell. It is difficult to investigate TME heterogeneity at the single‐cell level using bulk RNA‐seq. So, we collected three muscle invasive bladder cancer (MIBC) samples from our hospital and performed scRNA‐seq to overcome this limitation. A total of 19 852 cells were grouped into six major clusters after quality control and integration (Figure 2D, left). Clusters were annotated based on the well‐established marker genes as previous reported: epithelial cells (EPCAM), myeloid cells (LYZ), T cells (CD3D), fibroblasts (COL1A1), B cells (CD79A and CD19), and endothelial cells (PECAM1 and VWF)^[^ ^27^, ^28^ ^]^ (Figure S11A–H, Supporting Information). The UMIs and genes detected in the epithelial and immune cells are shown in Figure S11I,J (Supporting Information). As shown in Figure S12 (Supporting Information), CNVs accumulated mainly in EPCAM^+^ epithelial cells, confirming the malignant origin of the marked epithelial cells. According to an article reported by Peng et al.,^[^ ^32^ ^]^ we calculated the CNV scores of every epithelial cell and defined the epithelial cells with a CNV score of more than 0.02 as malignant epithelial cells, for further analysis. Interestingly, S100A5 was specifically expressed in bladder epithelial cells (Figure 2D, right). We focused on malignant epithelial cells and divided them into high S100A5 and low S100A5 expression groups. As shown in Figure 2E, GO pathways related to cytokine and chemokine secretion were downregulated in the high S100A5 expression group (Table S7, Supporting Information). Furthermore, the high S100A5 expression group showed significantly downregulated T cell proliferation and activation pathways (Figure 2F; Table S7, Supporting Information). S100A5 expression was the highest in epithelial cells, whereas no expression was observed in immune cells (such as CD4^+^ T, CD8^+^ T and NK cells) using another scRNA‐seq database containing two bladder cancer specimens (Figure 2G, GSE130001). We also analyzed S100A5 expression in peripheral blood cells (including CD4^+^ T, CD8^+^ T, NK cells, and et al.) and found that S100A5 was barely expressed in these cells (Figure 2G, GSE145281).

These results were further validated using public scRNA‐seq databases. Chen et al. reported the first and largest scRNA‐seq map of BLCA (PRJNA662018)^[^ ^27^ ^]^ and we clustered 59 045 cells from tumor tissues into eight major clusters after quality control and integration using their cohort (Figure 2H, left; Figure S13, Supporting Information). As expected, S100A5 was specifically expressed in the epithelial cells (Figure 2H, right). We then separated the malignant epithelial cells following the same role in Xiangya scRNA and found that malignant epithelial cells with high S100A5 expression significantly inhibited chemokine‐ and cytokine‐related pathways in both GO (Figure 2I; Table S8, Supporting Information) and KEGG (Figure 2J and Table S8, Supporting Information) analyses. In another scRNA‐seq cohort (GSE135337), we clustered 36 534 cells into five major clusters (Figure S14 and S15A, Supporting Information). S100A5 was specifically expressed on epithelial cells (Figure S15B, Supporting Information) and significantly negatively correlated with cytokine, chemokine and T cell infiltration related pathways (Figure S15C–E and Table S8, Supporting Information). Similar results were obtained for GSE145137 (Figure S15F–I and Table S8, Supporting Information).

### 2.5 S100A5 Inhibited CD8^+^ T Cells Recruitment through Decreasing Pro‐Inflammatory Chemokines Secretion

These results are mainly based on large and systematic bioinformatics analyses. We then validated these results in vitro and in vivo. S100A5 short hairpin RNA (sh‐S100A5) and S100A5‐cDNA (oe‐S100A5) and their negative controls (sh‐NC and oe‐vector respectively) were successfully transfected on human bladder cancer cell lines (T24 and 5637) (Figure S16A–D, Supporting Information). RNA‐Seq was performed on three parallel sh‐S100A5 and oe‐S100A5 cell lines. As expected, S100A5 was significantly downregulated in the sh‐S100A5 group compared to the oe‐S100A5 group (Figure 2K, T24; Figure S16E, 5637, Supporting Information). In contrast, several important chemokines, including CCL2, CCL5, CXCL10, and CXCL11 were significantly upregulated when S100A5 was downregulated (Figure 2K; Table S9, Supporting Information). Among these chemokines, CXCL10 and CXCL11 were reported to play vital roles in the process of recruiting immune cells.^[^ ^53^, ^54^ ^]^ Moreover, pathways related to cytokine/chemokine secretion/interaction (Figure S16F,G and Table S10, Supporting Information) and immune cell infiltration (Figure S16H and Table S10, Supporting Information) were significantly upregulated in the sh‐S100A5 cell line compared to those in the oe‐S100A5 cell line. Similar results were also found in the 5637 cell line (Figure S17A–C and Table S9 and S10, Supporting Information).

**Figure 3:**
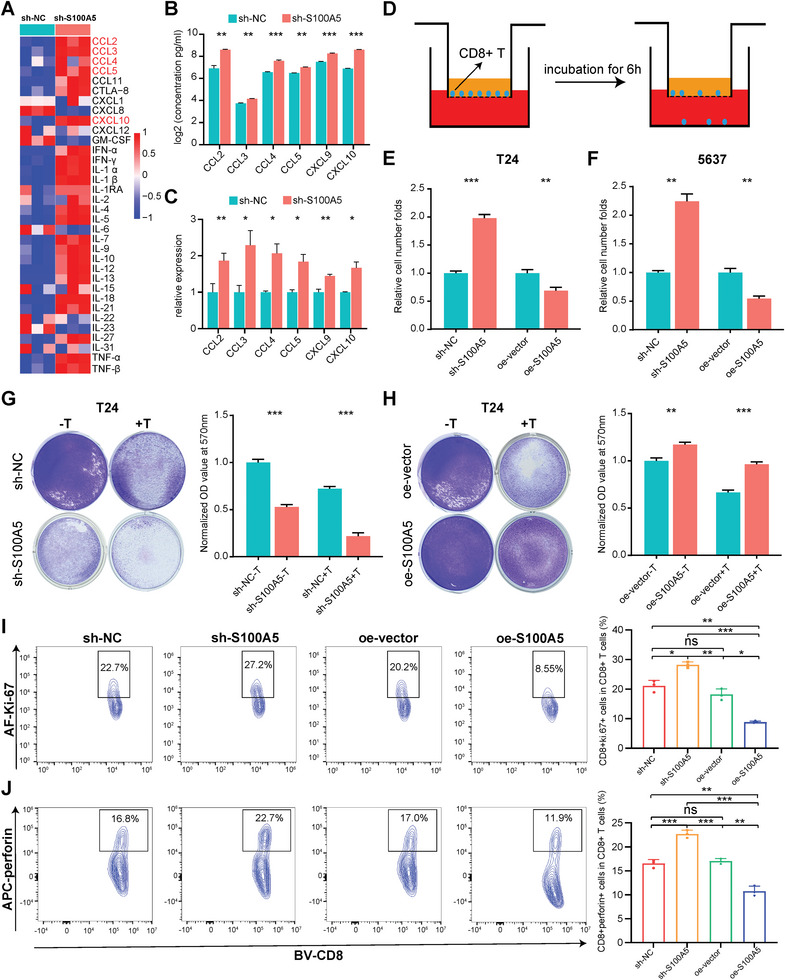
S100A5 inhibited CD8^+^ T cell recruitment and cancer cell killing process by regulating CD8^+^ T cell proliferation and cytotoxicity. A) Heatmap of multiple cytokines and chemokines detected by ProcartaPlex multiple immunoassays between S100A5 knockdown and the negative control groups in T24 cell culture supernatants. Red indicates higher secretion and blue indicates lower secretion. B,C) CCL2, CCL3, CCL4, CCL5, CXCL9, and CXCL10 levels detected using ELISA B) and RT‐PCR C), respectively, between S100A5 knockdown and negative control groups in the T24 cell line. D) Diagram of the CD8^+^ T cell migration assay. E,F) Relative migration of CD8^+^ T cells among S100A5 knockdown, S100A5 overexpression, and their negative controls in T24 cell line culture supernatants E) and 5637 cell line culture supernatants F). G) Representative images and histogram plots of the T cell‐mediated tumor cell‐killing assay between the S100A5 knockdown and negative control groups in the T24 cell line. The OD values were normalized to the mean value in the sh‐NC group without T cell co‐culture. H) Representative images and histogram plots of the T cell‐mediated tumor cell‐killing assay between S100A5 overexpression and negative control groups in the T24 cell line. The OD values were normalized to the mean value in the oe‐vector group without T cell co‐culture. I,J) Flow cytometry analysis shows different Ki‐67 I) and perforin J) expression on effector T cells after co‐cultured with S100A5 knockdown and overexpression of T24 cell lines and their negative control cell lines. ns, not statistically significant. *p < 0.05; **p < 0.01; ***p < 0.001.

To determine the key downstream cytokines/chemokines of S100A5, we applied ProcartaPlex multiple immunoassays to detect multiple cytokines and chemokines in cell culture supernatants from sh‐S100A5 and oe‐S100A5 cell lines. As shown in **Figure** **3**A (T24), the secretions of CCL2, CCL3, CCL4, CCL5, and CXCL10 were obviously increased when knocking down S100A5. Moreover, the secretion protein levels of these chemokines and another vital chemokines (CXCL9)^[^ ^53^ ^]^ were validated using ELISA. We found that all these chemokines were secreted at significantly higher levels in the sh‐S100A5 cell line (Figure 3B, T24). Furthermore, RT‐PCR results revealed that the mRNA expression levels of these chemokines were significantly higher when S100A5 was knocked down (Figure 3C, T24). In contrast, when S100A5 was overexpressed, the secreted protein levels of these chemokines were significantly decreased according to ProcartaPlex multiple immunoassays (Figure S17D,E, T24, Supporting Information) and ELISA (Figure S17F, T24, Supporting Information). mRNA expression was also significantly decreased, as shown by RT‐PCR (Figure S17G, T24, Supporting Information). Similar results were observed in 5637 cells (Figure S17H–K, Supporting Information). We conducted a CD8^+^ T cell migration assay to determine whether S100A5 inhibits CD8^+^ T cell infiltration by downregulating these chemokines (Figure 3D). We found that the knockdown and overexpression of S100A5 significantly increased and then decreased (Figure 3E, T24) CD8^+^ T cell migration, respectively, compared to their negative controls. Similar results were also found in the 5637 cells (Figure 3F, 5637). In summary, we found that S100A5 inhibited CD8^+^ T cell recruitment by decreasing chemokines secretion.

### 2.6 S100A5 Attenuated Effector T Cells Killing Cancer Cells through Inhibiting CD8^+^ T Cells Proliferation and Cytotoxicity

In addition to recruitment, we explored whether S100A5 expression could inhibit T cell function. First, we subdivided T cells into CD4^+^ and CD8^+^ T cells based on classical markers (including CD3D, CD3E, CD3G, CD4, CD8A, and CD8B) in Xiangya scRNA‐seq (Figure S18A–C, Supporting Information). Long et al. divided CD8^+^ T cells into progenitor exhausted T cells and terminally exhausted T cells, and revealed that patients with more progenitor exhausted T cells had a higher tumor‐killing efficiency and anti‐PD1 response rate.^[^ ^55^, ^56^ ^]^ We found that T cell cluster 1 had significantly lower progenitor exhaustion scores and higher exhaustion scores than cluster 3 (Figure S18D, Supporting Information); therefore, we defined cluster 1 as composed of terminally exhausted CD8^+^ T cells and cluster 3 as composed of progenitor exhausted CD8^+^ T cells (Figure S18E, Supporting Information). Then “CellChat” package was used to infer interactions and communication probability between high/low S100A5 expression epithelial cells and T cell subgroups (Figure S18F, Supporting Information). Surprisingly, epithelial cells with high S100A5 expression had a larger number of interactions and interaction weights with T cell subgroups than epithelial cells with low S100A5 expression (Figure S18G, Supporting Information), indicating that epithelial cells with high S100A5 expression had more communication with T cells and might inhibit their functions. Similar results were obtained for PRJNA662018 scRNA‐seq (Figure S19, Supporting Information).

We then performed a T cell‐mediated tumor cell‐killing assay to validate the results described above. In both the T24 and 5637 cell lines, S100A5 knockdown significantly enhanced the ability of T lymphocyte (activated from human peripheral blood mononuclear cells, PBMCs) to kill cancer cells (Figure 3G, T24; Figure S20A, 5637, Supporting Information), whereas S100A5 overexpression significantly inhibited this ability (Figure 3H, T24; Figure S20B, 5637, Supporting Information). Mechanistically, we found that the key proliferation marker Ki‐67 (Figure 3I) and cytotoxicity marker perforin (Figure 3J) were significantly upregulated in CD8^+^ T cells when lymphocytes were co‐cultured with the sh‐S100A5 cell line, but downregulated when lymphocytes were co‐cultured with the oe‐S100A5 cell line. When CD8^+^ T cells were directly cultured with recombinant S100A5, the proliferation marker Ki‐67 (Figure S20C, Supporting Information) and the cytotoxicity marker perforin (Figure S20D, Supporting Information) were significantly downregulated compared with the control groups. In addition, the exhausted marker PD‐1 was significantly upregulated when cultured with recombinant S100A5 (Figure S20E, Supporting Information).

Interestingly, when cancer cell lines were not co‐cultured with T lymphocytes (Figure 3G,H, Figure S20A,B, Supporting Information), knockdown or overexpression of S100A5 alone inhibited or promoted cancer cell proliferation, respectively. We speculated that S100A5 may also act as an oncogene and promote BLCA proliferation and invasion. MTT cell viability assays revealed that knockdown (Figure S21A, Supporting Information) and overexpression (Figure S21B, Supporting Information) of S100A5 significantly inhibited and promoted the proliferation of bladder cancer cells, respectively. In addition, the colony formation assay confirmed that the knockdown (Figure S21C, Supporting Information) and overexpression (Figure S21D, Supporting Information) of S100A5 significantly inhibited and promoted the colony formation ability of bladder cancer cells, respectively. In addition, a wound healing assay revealed that S100A5 promoted bladder cancer cell invasion (Figure S21E–G, Supporting Information). In summary, we found that S100A5 could act as an oncogene and inhibit cancer cell death by inhibiting CD8^+^ T cell proliferation and cytotoxicity.

### 2.7 Targeting S100A5 Enhanced the Efficacy of Anti‐PD‐1 Treatment and CD8^+^ T Cells Recruitment

**Figure 4:**
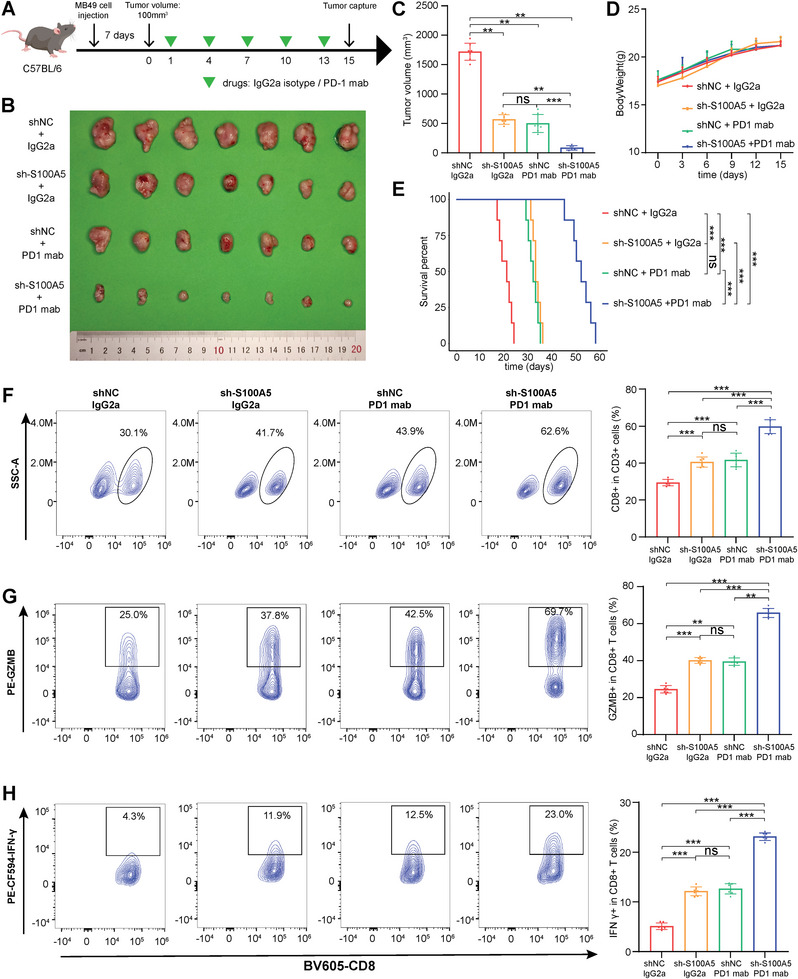
Downregulation of S100A5 enhanced the efficacy of anti‐PD‐1 treatment and CD8^+^ T cells recruitment in vivo. A) In vivo experimental procedure and treatment schedule. B) Tumor images among different experimental groups after mice sacrifice. C) Histogram plot of tumor volumes among different experimental groups after mice sacrifice. D) Scatter diagram plot of body weights among different groups during experimental procedure. E) Kaplan‐Meier plot of survival percent among different experimental groups. F–H) Representative contour plots and the proportion of CD8^+^ T cells in T cells F), GZMB^+^ cells in CD8^+^ T cells G), and IFN‐*γ* ^+^ cells in CD8^+^ T cells H). ns, not statistically significant; **p < 0.01; ***p < 0.001.

Considering that S100A5 could inhibit CD8^+^ T cell infiltration and proliferation in vitro, we further explored whether it could affect the efficacy of anti‐PD‐1 treatment preclinically. We constructed a subcutaneous bladder cancer model by subcutaneously injecting S100A5 KD and negative control MB49 cells (Figure S22A,B, Supporting Information). Consistent with the results for the T24 cell line, we found that the knockdown of S100A5 inhibited BLCA proliferation and invasion using MTT (Figure S22C, Supporting Information), colony formation (Figure S22D, Supporting Information), and wound healing assays (Figure S22E, Supporting Information) on the MB49 cell line. The in vivo experimental procedure and treatment schedule were illustrated in **Figure** **4**A. As shown in Figure 4B,C, S100A5 knockdown or anti‐PD‐1 treatment alone significantly suppressed the tumor burden in vivo. However, S100A5 knockdown plus anti‐PD‐1 treatment showed the highest efficacy in inhibiting tumor burden, indicating that S100A5 downregulation could enhance the efficacy of anti‐PD‐1 treatment. In addition, there was no difference in body weight between the groups of mice (Figure 4D). Moreover, knockdown of S100A5 with anti‐PD‐1 treatment showed the greatest ability to prolong survival in mice (Figure 4E).

**Figure 5:**
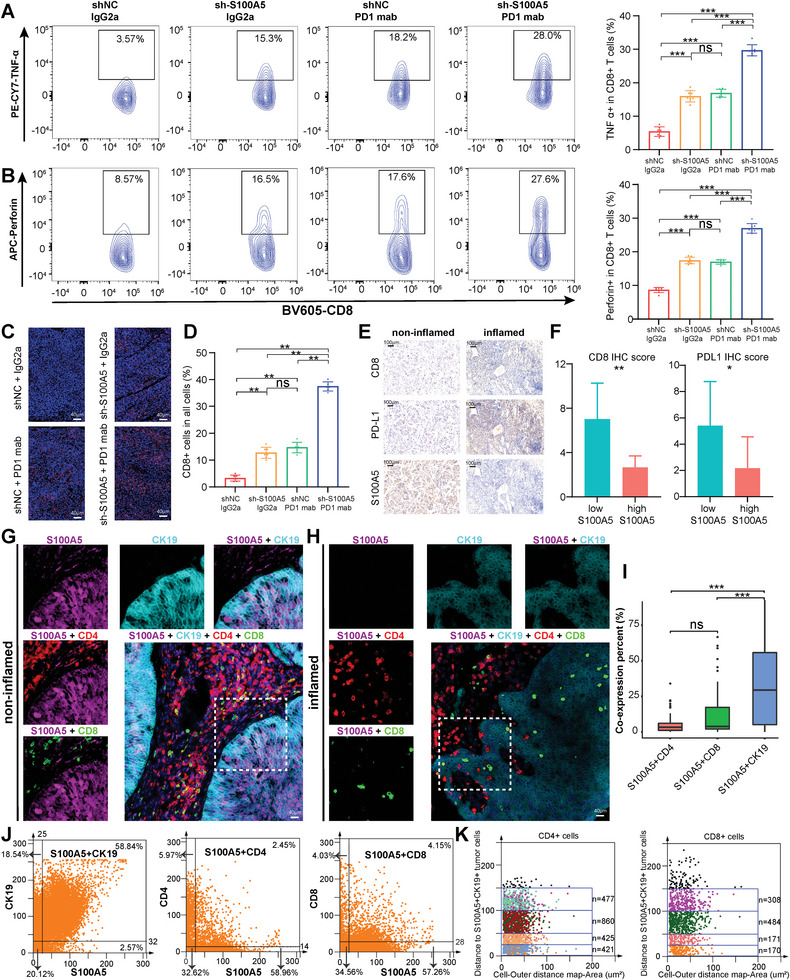
IHC and TissueFAXS Cytometry panoramic tissue quantification assay depicted the spatial exclusive relationship between S100A5 and effector T cells in the TME. (A‐B) Representative contour plots and the proportion of TNF‐*α* ^+^ cells in CD8^+^ T cells A), and perforin^+^ cells in CD8^+^ T cells B). C) Representative immunofluorescence (IF) images of CD8 staining among different experimental groups. Scale bars, 40 µm. D) Percent of CD8^+^ cells in all cells corresponding to IF images. E) Representative images of the expression patterns of CD8, PD‐L1 and S100A5 in non‐inflamed and inflamed TME using IHC. Scale bars, 100 µm. F) CD8 and PD‐L1 IHC scores between high and low S100A5 groups. G,H) Representative multi‐color staining of non‐inflamed G) and inflamed H) phenotypes of patients with bladder cancer: S100A5 (purple), CK19 (azure), CD4 (red), CD8 (green), DAPI (blue). I) The histograms of different S100A5^+^CD4^+^, S100A5^+^CD8^+^, and S100A5^+^CK19^+^ percent cells among the whole TMA. J) The flow‐like cytometer plots show the percent of S100A5^+^CK19^+^, S100A5^+^CD4^+^, and S100A5^+^CD8^+^ cells respectively. K) The spatial distribution of CD4^+^ and CD8^+^ T cells within the distance gradients of S100A5^+^CK19^+^ tumor cells (0–25 µm, 25–50 µm, 50–100 µm, and 100–150 µm). ns, not statistically significant. *p < 0.05; **p < 0.01; ***p < 0.001.

Furthermore, the tumor tissues were digested into single‐cell suspensions, and flow cytometry analysis was applied to explore the composition of immune cells. As shown in Figure S23A–D (Supporting Information), the proportions of leukocytes (CD45^+^), lymphoid cells (CD45^+^CD11b^−^), and T cells (CD3^+^) were generally the same in each group. Then we further explored the proportion of CD8^+^ T cells and their cytotoxicity indicators (including GZMB, IFN‐*γ*, TNF‐*α* and Perforin) among each group. We found that S100A5 knockdown or anti‐PD‐1 treatment alone significantly increased the infiltration of CD8^+^ T cells (Figure 4F) and enhanced their cytotoxicity by upregulating GZMB (Figure 4G), IFN‐*γ* (Figure 4H), TNF‐*α* (**Figure** **5**A) and perforin (Figure 5B). However, S100A5 knockdown with anti‐PD‐1 treatment showed the highest efficacy in recruiting CD8^+^ T cells and upregulating these cytotoxicity indicators (Figure 4F–H and Figure 5A,B). In addition, immunofluorescence (IF) analysis confirmed that both S100A5 knockdown and anti‐PD‐1 treatment promoted CD8^+^ T cell infiltration, whereas the combination of S100A5 knockdown and anti‐PD‐1 treatment showed the highest efficiency (Figure 5C,D).

The above in vivo experiment was still based on the S100A5 knockdown cell line, and we designed a nanomedicine carrying siS100A5 for the direct targeting of S100A5. Liposomes are one of the most widely used carriers of siRNAs because of their high loading efficiency, reliable drug protection, good biocompatibility, and targeted delivery.^[^ ^57^, ^58^, ^59^ ^]^ We successfully synthesized liposome and loaded them with siS100A5. Transmission electron microscopy (TEM) showed the morphology of liposome@siS100A5 (Figure S24A, Supporting Information). The average DLS of the liposome@siS100A5 was 205.6 nm (a little bit larger than that of liposome alone) with polydispersity index (PDI) of 0.16 (Figure S24B, Supporting Information). The surface charge of the liposome was 43.6 mV and turned to be 25.8 mV when siS100A5 (−11.6 mV) was loaded onto it (Figure S24C, Supporting Information). Standard curve shows the relationship between fluorescence intensity and siS100A5 concentration (Figure S24D,E, Supporting Information), and with loading efficiency of 78%.

The anti‐tumor efficiency of liposome@siS100A5 was tested in the following four groups: (1) liposome + IgG2a isotype, (2) liposome@siS100A5 + IgG2a isotype, (3) liposome + anti‐PD‐1, (4) liposome@siS100A5 + anti‐PD‐1. We found that the injection of liposome@siS100A5 alone significantly suppressed the tumor burden, and this effect was synergized when combined with anti‐PD‐1 treatment (Figure S25A,B, Supporting Information). There was no difference in the body weight between the groups of mice during the treatment procedure (Figure S25C, Supporting Information). To test the toxicity of liposome@siS100A5 in the blood system, we detected mouse whole blood and blood biochemistry indices after intravenous injection of liposome@siS100A5 and revealed that all these blood indices were within the normal range (Figure S25D, Supporting Information). In addition, H&E staining of the heart, liver, spleen, lungs, and kidneys revealed that our treatment caused minimal damage to the major organs (Figure S25E, Supporting Information). We found that S100A5 was mainly expressed in malignant epithelial cells, whereas very low expression was observed in immune cells and peripheral blood cells using scRNA‐seq analysis. This could explain why targeting S100A5 had minimal adverse effects on normal tissues and blood system.^[^ ^49^ ^]^ Furthermore, flow cytometry analysis (Figure S25F, Supporting Information) revealed that targeting S100A5 could significantly promote CD8^+^ T cell infiltration, and this effect could be synergized when combined with anti‐PD‐1 treatment (Figure S25G, Supporting Information). In summary, we revealed that the downregulation of S100A5 and targeting S100A5 could enhance the efficacy of anti‐PD‐1 treatment and promote CD8^+^ T cells recruitment in vivo.

### 2.8 The Spatial Exclusive Relationship between S100A5^+^ Malignant Cells and Effector T Cells

Tissue microarrays (TMAs) containing 50 BLCA samples receiving no prior treatment were prepared. We defined the samples as “inflamed phenotype” samples in which CD8^+^ T cells were located in the tumor parenchyma and defined the samples as “non‐inflamed phenotype” samples in which CD8^+^ T cells were located in the stroma (not in parenchyma) or no CD8^+^ T cells either in parenchyma or stroma. The representative image in Figure 5E shows the exclusive role of S100A5 between CD8 and PD‐L1 at the protein level. Moreover, both the CD8 and PD‐L1 IHC scores were significantly higher in the low S100A5 group (Figure 5F). The TissueFAXS cytometry panoramic tissue quantification assay was further applied to reveal the spatially exclusive role of S100A5^+^ bladder cancer cells and CD4^+^ and CD8^+^ T cells. Figure 5G,H shows representative images of multi‐color staining. In non‐inflamed tumors, the wide expression of S100A5 in tumor cells inhibited the infiltration of CD4^+^ and CD8^+^ T cells into the tumor region (Figure 5G, non‐inflamed). In contrast, none of S100A5 expression tumors exhibited an inflamed phenotype, with numerous CD4^+^ and CD8^+^ T cells infiltrating the tumor regions (Figure 5H, inflamed). The co‐expression of S100A5 on tumor cells and the exclusive role of S100A5 between CD4^+^ T and CD8^+^ T cells reached statistical significance in the whole TMA cohort (Figure 5I). In addition to CD4^+^ and CD8^+^ T cells, S100A5 was barely expressed in NK (CD16^+^) cells and macrophages (CD68^+^) (Figure S26A,B, Supporting Information). Moreover, the flow‐like cytometry plots show that S100A5 was positively expressed mainly in CK19^+^ tumor cells (58.84%), but almost not in CD4^+^ T cells (2.45%) or CD8^+^ T cells (4.15%, Figure 5J). For further spatial analysis, we quantified the number of CD4^+^ and CD8^+^ T cells within the distance gradients of S100A5^+^CK19^+^ tumor cells (0‐25 µm, 25–50 µm, 50–100 µm, and 100–150 µm). As expected, the greater the distance from S100A5^+^CK19^+^ cells, the more CD4^+^ and CD8^+^ T cells showed an increasing trend (Figure 5K), which confirmed the spatially exclusive relationship between S100A5 and effector T cells.

### 2.9 S100A5 and Immune Checkpoint Blockade (ICB) Response

**Figure 6:**
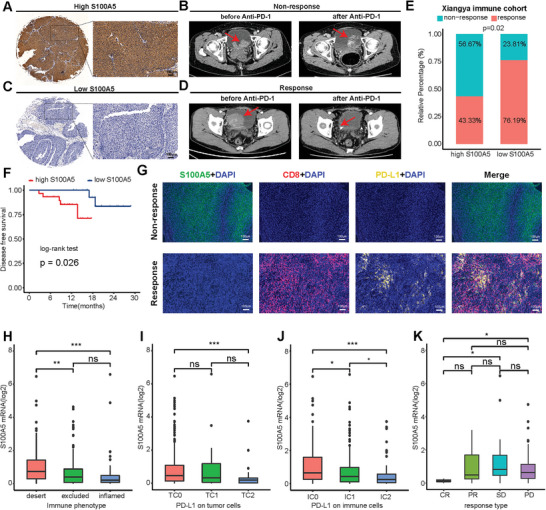
Relationship between S100A5 expression and immune checkpoint blockade (ICB) response. A) Representative immunohistochemical (IHC) image of high S100A5 expression patient. Scale bar, 100 µm. B) Representative CT image for patient with progressive disease after anti‐PD‐1 treatment. C) Representative IHC image of low S100A5 expression patient. Scale bar, 100 µm. D) Representative CT image for patient with complete response after anti‐PD‐1 treatment. E) Relative percentage of patients with clinical response to immunotherapy between different S100A5 expression groups in Xiangya immune cohort. Yellow, immunotherapy response group; Blue, non‐response group. F) Disease‐free survival (DFS) of patients with different S100A5 IHC scores in Xiangya immune cohort. G) Representative multi‐color IF images for S100A5 (green), CD8 (red), PD‐L1 (yellow) and DAPI (blue) in non‐response and response group patients. Scale bars, 100 µm. H) Expression of S100A5 on desert, excluded, and inflamed immune phenotypes in IMvigor210 cohort. I,J) Expression of S100A5 on patients with different PD‐L1 expression on tumor cells I) and immune cells J) in IMvigor210 cohort. K) Correlation between S100A5 expression values and immunotherapy response in the desert phenotype of IMvigor210 cohort. Different color represents different response type. CR: complete response; PR: partial response; SD: stable disease; PD: progressive disease. ns, not statistically significant. *p < 0.05; **p < 0.01; ***p < 0.001.

As S100A5 shaping a non‐inflamed TME, theoretically, lower S100A5 expression could be associated with favorable ICB response rates. Therefore, we determined whether S100A5 expression could predict ICB response. As our previous study reported, we have built Xiangya immune cohort containing 51 BLCA patients treated with ICB from our hospital.^[^ ^19^ ^]^ Patients with higher S100A5 expression (**Figure** **6**A) showed progressive disease after anti‐PD‐1 treatment (Figure 6B). In contrast, patients with lower S100A5 expression (Figure 6C) achieved a complete response after anti‐PD‐1 therapy (Figure 6D). According to the pathological response, these patients were categorized into the complete response (CR), partial response (PR), stable disease (SD), and progressive disease (PD) groups. We further divided CR and PR patients into the response group, whereas SD and PD patients were classified into the non‐response group. Low S100A5 IHC score group (IHC score less than 6) possessed significantly more patients responding to anti‐PD‐1 treatment than high S100A5 IHC score group (IHC score no less than 6) among the whole TMA cohort (Figure 6E). Importantly, the patients with lower S100A5 IHC scores exhibited significantly higher disease‐free survival (DFS) rates than those with higher scores (Figure 6F). Moreover, multi‐color IF images showed a negative correlation between S100A5 expression, CD8^+^ T cells infiltration, and PD‐L1 expression (Figure 6G), revealing the mechanism of response or non‐response to treatment. In a large immunotherapy cohort of BLCA (IMvigor210), we found that S100A5 expression was highest in the desert immune phenotype, TC0 (lowest PD‐L1 expression on tumor cells), and IC0 (lowest PD‐L1 expression on immune cells) (Figure 6H–J). Furthermore, we found that patients with CR expressed significantly lower S100A5 than patients with PD or SD in IMvigor210 (Figure 6K).

## 3 Discussion

S100 family proteins, as a type of small molecular EF‐hand calcium‐binding proteins, play vital roles in the development and progression of numerous types of carcinomas and show prognostic value and are potential novel targets for treatment.^[^ ^60^ ^]^ The vital role of S100 proteins in carcinomas has been widely reported in melanoma, breast carcinoma, and lung carcinoma. S100A4 not only activates the nuclear factor‐kappa B (NF‐*κ*B) pathway and leads to the release of the tumor necrosis factor (TNF)‐*α*,^[^ ^61^ ^]^ but also shapes an inflamed TME by inducing the secretion of IL‐8 and C—C chemokine ligand 2 (CCL2),^[^ ^14^ ^]^ playing an oncogenic role in malignant melanoma. In breast carcinoma, multiple S100 proteins are dysregulated, including S100A2, S100A4, S100A6‐9, and S100A11.^[^ ^60^ ^]^ Among them, S100A8 and S100A9 can recruit MDSCs to maintain an immune suppression state in the TME,^[^ ^12^ ^]^ indicating the key roles of S100 family proteins in modulating the TME. For lung carcinoma, S100A4 expression was associated with worse survival outcomes in NSCLC and promoted cancer invasion through the NF‐*κ*B/MMP9 signaling pathway.^[^ ^62^, ^63^ ^]^ Recently, Liu et al. revealed that S100A7 can shape an immunosuppressive TME and decrease the efficacy of immunotherapy in LUSC.^[^ ^64^ ^]^ However, the expression patterns and functions of S100 proteins are cancer specific.^[^ ^60^ ^]^ Regarding BLCA, only Yao et al. reported that S100A2‐3, S100A5, S100A7‐9, S100A14‐16, and S100P were significantly higher in BLCA tissues using real‐time PCR.^[^ ^16^ ^]^ The comprehensive roles of S100 proteins in the TME and immunotherapy remain unclear, especially in BLCA. In this study, for the first time, we comprehensively analyzed the expression patterns and immunological roles of multiple S100 family proteins in BLCA and found that S100A5 might have the most important value in BCLA. Furthermore, pan‐cancer analyses through 33 types of carcinomas revealed that the immunosuppressive role of S100A5 in the TME was most obvious in BLCA.

As a high TMB tumor, an increasing number of studies have demonstrated the efficacy of ICIs in BLCA. Five ICIs have gained approval from the FDA: atezolizumab, durvalumab, avelumab, pembrolizumab, and nivolumab.^[^ ^2^, ^4^, ^5^ ^]^ However, only a minority of patients respond to ICI treatment, and most patients benefit little from ICIs and suffer from treatment‐related toxicity, suggesting an urgent need to identify biomarkers of response to ICIs.^[^ ^5^, ^6^ ^]^ To date, numerous biomarkers have been found to predict ICI response. PD‐L1 expression in tumor cells has been reported to be associated with higher response rates; however, patients without PD‐L1 expression also show an ICI response.^[^ ^65^ ^]^ Other biomarkers include TMB, alteration of DNA damage repair genes, and interferon‐*γ* associated genes; however, all these biomarkers need additional validation for clinical application.^[^ ^65^ ^]^ As an immune rheostats, the treatment function of ICIs largely depends on pre‐existing anticancer immune responses.^[^ ^50^ ^]^ Therefore, the immunosuppressive state of TME can be a major obstacle to ICI efficacy. The TME is composed of tumor cells, fibroblast cells, vascular endothelial cells, immune cells, extracellular matrix, and extracellular soluble molecules, and plays a vital role in cancer development and evasion of the host immune system.^[^ ^8^ ^]^ Based on the presence or absence of T cells in the tumor parenchyma, the TME can generally be divided into two profiles: non‐inflamed and inflamed tumors.^[^ ^9^ ^]^ In this study, we used systematic bioinformatics analysis (including bulk RNA‐seq and scRNA‐seq) to reveal that S100A5 is associated with a non‐inflamed TME phenotype in BCLA. Inflamed tumors, characterized by CD4^+^ and CD8^+^ T cell infiltration and elevated signatures of immune activation, are sensitive to immunotherapy.^[^ ^7^, ^8^ ^]^ We validated that S100A5 inhibited CD8^+^ T cell infiltration, whereas its knockdown significantly promoted infiltration and enhanced the cytotoxicity of CD8^+^ T cells in vitro and in vivo.

There are two fundamental strategies for cancer immunotherapy: “immune enhancement” strategy, which focuses on increasing immune activation, and “immune normalization”, which focuses on restoring immune cell deficiency in the TME.^[^ ^49^ ^]^ Immune‐related adverse events (irAEs) limit the clinical application of “immune enhancement” strategy, while “immune normalization” strategies, such as ICIs show objective response rates without obvious irAEs. However, ICIs have shown no therapeutic efficacy in some patients, partly because of a lack of pre‐existing anticancer immune responses. The combination of “immune enhancement” and “immune normalization” strategies could be more effective. For example, combined therapy with anti‐PD‐1 and CTLA4 therapy has shown much higher effectiveness.^[^ ^11^ ^]^ The key for developing “immune enhancement” strategy is to avoid irAEs. Using sc‐RNA‐seq data, we found that S100A5 was specifically expressed in tumor cells and barely expressed in other cells, both in the TME and blood. These results could be beneficial for developing anti‐S100A5 drugs that are tumor cell‐specific and less toxic. Niclosamide, an inhibitor of S100A4, which inhibit colorectal cancer (CRC) progression, has entered phase II clinical trials for the treatment of metastatic CRC.^[^ ^66^, ^67^ ^]^ Based on our findings, anti‐S100A5 therapy might have the ability to turn “cold” tumors into “hot” tumors and show higher effectiveness in combination with ICIs. Our work is the first step towards the development of anti‐S100A5 drugs that can reverse the TME without obvious toxicity.

Unsupervised clustering of transcriptome profiling data revealed that BLCA can be divided into molecular subtypes that can be used to predict prognosis and therapeutic options, including chemotherapy and immunotherapy.^[^ ^35^ ^]^ Several molecular subtype models of BLCA have been established, including CIT,^[^ ^37^ ^]^ Lund,^[^ ^38^ ^]^ MDA,^[^ ^39^ ^]^ TCGA,^[^ ^40^ ^]^ Baylor,^[^ ^41^ ^]^ and UNC.^[^ ^42^ ^]^ However, the differences in the number, relative size, and names of the subtypes between these molecular subtype models inhibit their clinical application. Thus, Kamoun et al. reported a consensus molecular classification by conducting a network analysis of these six molecular subtype models.^[^ ^35^ ^]^ Consensus molecular classification has promoted the clinical use of molecular subtypes. However, all of these molecular subtypes are built based on transcriptome profiling data (RNA‐seq or microarray), which can be complex and expensive for clinical applications. We found that S100A5 expression could accurately predict all seven classification systems with high accuracy (AUC > 0.85), and validated this prediction value using other public databases and our own RNA‐seq cohort. Our findings greatly simplify molecular classification systems and promote their clinical applications. Basal subtypes have been reported to possess more cytotoxic lymphocytes and NK cell infiltration, and may be more sensitive to immunotherapy.^[^ ^35^ ^]^ This is consistent with our previous finding that S100A5 shaped a non‐inflamed phenotype of BLCA in terms of the molecular subtypes of BLCA.

There are some limitations of our study. First, our validation cohorts contained RNA‐seq and microarray data, and different cohorts may have led to batch effects and bias in our findings. Second, although S100A5 could mediate tumor immune evasion by regulating chemokine secretion and CD8^+^ T cell cytotoxicity, further detailed mechanisms are required. Third, to turn cold bladder tumors into hot ones, S100A5 small‐molecule inhibitors need to be developed in future research.

## 4 Conclusions

S100A5 shapes a non‐inflamed tumor microenvironment in BLCA by inhibiting the secretion of pro‐inflammatory chemokines and the recruitment and cytotoxicity of CD8^+^ T cells. Targeting S100A5 converts cold tumors into hot tumors, thus enhancing the efficacy of ICB therapy in BLCA.

## 5 Experimental Section

### Sources of Datasets and Preprocessing—Xiangya Cohorts

As reported in the previous studies,^[^ ^17^, ^18^ ^]^ 60 fresh bladder cancer samples was collected (only 57 qualified samples) and 13 paired adjacent normal samples without prior treatment from the Xiangya Hospital and performed RNA sequencing (RNA‐seq) of the qualified samples. Among the 57 patients, 56 were successfully followed up. This cohort was named the Xiangya BLCA cohort (GSE188715). In addition, a tissue microarray (TMA) containing 50 BLCA samples was built and 28 paired adjacent normal samples without prior treatment and another TMA containing 51 BLCA samples with immune checkpoint blockade (ICB) treatment.^[^ ^19^ ^]^

### Sources of Datasets and Preprocessing—Public Databases

For the Cancer Genome Atlas (TCGA) database, the fragments per kilobase per million mapped fragments (FPKM) value of RNA‐seq data of 33 types of cancer from the UCSC Xena data portal (https://xenabrowser.net/) was downloaded.^[^ ^20^ ^]^ Specific to TCGA‐BLCA, the FPKM value was transformed into transcripts per kilobase million (TPM). Duplicate patients and those without complete clinical information were filtered out. A total of 403 patients with detailed clinical information were included in the analysis. Gene Expression Omnibus (GEO) (https://www.ncbi.nlm.nih.gov/geo/) databases with more than 50 BLCA samples were downloaded using “GEOquery” R package, including: GSE87304 (GPL22995), GSE48276 (GPL14951), GSE48075 (GPL6947), GSE120736 (GPL10558), GSE31684 (GPL570), GSE32894 (GPL6947), GSE69795 (GPL14951), and then transformed the gene symbol using the corresponding GPL files. E‐MTAB‐1803, a public database containing 85 BLCA samples, was downloaded from The European Bioinformatics Institute (https://www.ebi.ac.uk/). Moreover, the expression matrix was downloaded and clinical information of the BLCA immunotherapy cohort (IMvigor210 cohort) from http://researchpub.Gene.com/imvigor210corebiologies/.^[^ ^21^ ^]^

### Sources of Datasets and Preprocessing—Calculating of TME Cell Infiltration

Tracking tumor immunophenotype (TIP) was a web‐based analytical platform for analyzing the level of seven‐step cancer immune cycles (http://biocc.hrbmu.edu.cn/TIP/).^[^ ^22^ ^]^ The TPM data was uploaded of the TCGA‐BLCA and Xiangya cohorts onto TIP and downloaded the cancer‐immune cycle. The single‐sample gene set enrichment analysis (ssGSEA) algorithm was used to calculate the infiltration of 28 immune cells into the BLCA TME. The gene set for each immune cell was obtained from a study by Charoentong et al.^[^ ^23^ ^]^ To eliminate the influence of this algorithm, six other algorithms were used to calculate the infiltration of immune cells: CIBERSORT, EPIC, mMCP‐counter, quanTIseq, TIMER, and xCell. Among these algorithms, EPIC, quanTIseq, TIMER, and xCell algorithms were performed using “deconvolute” function of the “immunedeconv” R package. For CIBERSORT, LM22 was downloaded, an annotated gene set, to define the 22 immune cell subtypes from the CIBERSORT web portal (http://cibersort.stanford.edu/). For mMCP‐counter, the genes and probesets files were downloaded from https://github.com/ebecht/MCPcounter and used “MCPcounter.estimate” function of the “MCPcounter” R package.^[^ ^24^ ^]^

### Other Immunological Characteristics of TME

MHC, receptors, chemokines, and immune stimulators (122 immunomodulators) were identified based on a study by Charoentong et al.^[^ ^23^ ^]^ Moreover, 22 ICI genes were identified based on the study by Auslander et al.^[^ ^25^ ^]^ Eighteen genes for calculating the T cell‐inflamed score (TIS) were identified based on the study by Ayers et al.^[^ ^26^ ^]^ Pan‐cancer TIS and the effector genes of CD8^+^ T cells, dendritic cells (DCs), macrophages, natural killer (NK) cells, and type 1 T helper (Th1) cells were collected as previously described.^[^ ^17^ ^]^

### Analysis Process of Single Cell RNA Sequencing (scRNA‐seq)

Three samples from muscle invasive bladder cancers (MIBCs) were obtained from Xiangya hospital and performed scRNA‐seq in OE Biotech Co, Ltd (Shanghai, China), named Xiangya scRNA cohort. The detailed preparation of single‐cell suspensions, sequencing of droplet‐based single‐cells and raw data processing have been reported in previous studies.^[^ ^18^, ^27^ ^]^ After cell ranger processing, the count matrixes were converted into Seurat object by “Seurat” R package (version 4.1.0). Cells were regarded with unique molecular identifier (UMI) numbers less than 1000, gene numbers less than 200, log10GenesPerUMI less than 0.70 and mitochondrial‐derived UMI counts over 20% as low‐quality cells and filtered out these cells. Then the count matrixes were normalized using “NormalizeData” function and regressed out the effect of mitochondrial ratio using “SCTransform” function. “SelectIntegrationFeatures” function was used to find the top 3000 variable features for integration and “PrepSCTIntegration” function was used to convert the Seurat object to SCT list object for integration. Then, “FindIntegrationAnchors” and “IntegrateData” functions were used to integrate the three samples based on the top 3000 variable features selected above. This process regressed the potential batch effect and created a new matrix with 3000 variables. Then principal component analysis (PCA) was conducted using “RunPCA” function and ran *t*‐distributed stochastic neighbor embedding (*t*SNE) based on “pca” reduction using “RunTSNE” function. Finally, the main cells clusters were identified using “FindNeighbors” and “FindClusters” function (res = 0.4) and visualized the cells clusters based on *t*SNE plot. Clusters were annotated combined by “SingleR” package and well‐established marker genes reported previously. In particular, cells annotated as T cells were further clustered into subclusters. All parameters of the functions used above were set to default.

In addition, a public scRNA‐seq dataset (PRJNA662018^[^ ^27^ ^]^) containing eight BLCA and three normal tissue samples was downloaded. Another public scRNA‐seq dataset (GSE135337^[^ ^28^ ^]^) containing seven BLCA tissue samples, was downloaded from the supplementary materials of the GEO database. The analysis processes were similar to those used for Xiangya scRNA cohort. Specifically, out cells were filtered with UMI numbers less than 1000, genes numbers less than 250, log10GenesPerUMI less than 0.80, and mitochondrial‐derived UMI counts over 10%. Moreover, the log2TPM matrix of one primary bladder cancer patient (two other matrices were not available in the GEO database) was downloaded and cell type information from the supplementary file of GSE145137.^[^ ^29^ ^]^ Because all the cluster and cell type information could be downloaded, the above scRNA‐seq analysis processes was omitted and directly used “VlnPlot” function to visualize the expression of S100A5 in the TME of BLCA. Two other scRNA‐seq databases (GSE130001^[^ ^30^ ^]^ and GSE145281^[^ ^31^ ^]^) were analyzed using TISCH (http://tisch.comp‐genomics.org/home/).

### Estimation of Copy Number Variations (CNVs) in Epithelial Cells

Setting the epithelial cells from three normal tissue samples in PRJNA662018 scRNA as references, the “InferCNV” package was used to detect the initial CNVs values of epithelial cells from tumor tissue samples in Xiangya scRNA and PRJNA662018 scRNA. The initial CNVs value of each cell was re‐standardized and made the background value as 0. The mean of the squares of all the initial gene CNVs values was then defined as the final CNVs score for each cell. Epithelial cells from tumor tissue samples with final CNVs scores more than 0.02 were regarded as malignant epithelial cells for downstream analysis. This process was performed as described by Peng et al.^[^ ^32^ ^]^

### Cell Chat Analysis

The “CellChat” R package (version 1.6.1) was used to perform Cell chat analysis.^[^ ^33^ ^]^ According to the official workflow, the normalized expression data of malignant epithelial cells was loaded, T cells, and cell group information into CellChat. Receptor‐ligand interactions were screened using the “CellChatDB.human” database as a reference. “computeCommunProb,” “filterCommunication,” “computeCommunProbPathway,” and “aggregateNet” functions were used to figure out the potential ligand‐receptor interactions between high/low S100A5 expression epithelial cells and T cell subgroups. All parameters of the functions used above were set to default.

### Pathway Enrichment Analysis

For bulk RNA‐seq data, the empirical Bayesian approach of the “limma” R package was used to filter the differentially expressed genes (DEGs). The significance criterion was set as |log2FC| >1 and adj.p.value <0.05, for filtering DEGs. For scRNA‐seq data, DEGs between different S100A5 expression patterns on epithelial cells were recognized using Findmarker function in “Seurat” R package. Gene Set Enrichment Analysis (GSEA) was performed based on genes ordered by fold change (FC). Gene sets from the Gene Ontology (GO) and Kyoto Encyclopedia of Genes and Genomes (KEGG) were downloaded from the Molecular Signatures Database (MSigDB) (https://www.gsea‐msigdb.org/gsea/index.jsp).^[^ ^34^ ^]^ In addition, 12 bladder cancer signatures were collected that could represent different processes of different molecular subtypes of BLCA from Kamoun's study.^[^ ^35^ ^]^ Then the enrichment scores of these signatures were calculated using ssGSEA algorithm in “GSVA” R package.^[^ ^36^ ^]^

### Determining the Molecular Subtypes of BLCA

There were seven established molecular subtypes of BLCA: Cartes d'Identité des Tumeurs‐Curie (CIT),^[^ ^37^ ^]^ Lund,^[^ ^38^ ^]^ MDAnderson Cancer Center (MDA),^[^ ^39^ ^]^ TCGA,^[^ ^40^ ^]^ Baylor,^[^ ^41^ ^]^ University of North Carolina (UNC),^[^ ^42^ ^]^ and consensus subtypes.^[^ ^35^ ^]^ BLCA patients were stratified into all the molecular subtypes above using “ConsensusMIBC” and “BLCAsubtyping” R packages. To unify these seven molecular models, patients with BLCA can generally be divided into basal and luminal subtypes based on the relationships between the different models.^[^ ^35^ ^]^ The predictive accuracy of S100A5 for the molecular subtypes was depicted using receiver operating characteristic (ROC) curves.

### Malignant Bladder Cell Lines

Human malignant bladder cell lines T24 and 5637 were purchased from the American Type Culture Collection (ATCC, Manassas, VA, USA) and cultured in DMEM or RPMI‐1640 medium (Invitrogen), respectively. The murine malignant bladder cell line MB49 was purchased from Meisen CTCC (Jinhua, China) and cultured in DMEM. All the media were supplemented with 10% FBS (Gibco), 1% penicillin and streptomycin (Invitrogen). The condition of incubator was set as 37 °C with 5% CO_2_.

### Stable Cell Transfection

Lentiviral vectors with S100A5‐short hairpin RNA (sh‐S100A5) and S100A5‐cDNA (oe‐S100A5) were purchased from Shanghai Genechem. The targeting sequences were as followings: sh‐S100A5 #Human 1: cgACTTCTTTCTAGAGGACAA; sh‐S100A5 #Human 2: gcAGCATCGATGACTTGATGA; sh‐S100A5 #Human 3: gtGACCACGTTTCACAAATAT; sh‐S100A5 #Mouse 1: gcCTACAATGACTTCTTCCTA; sh‐S100A5 #Mouse 2: gaAGGAGAGCAGCATTGATAA; sh‐S100A5 #Mouse 3: gtCACCACTTTCCATAAATAT. Then, according to the manufacturer's protocol, bladder cancer cell lines were transfected using Turbofect (Thermo Fisher Scientific) in DMEM or RPMI‐1640. After transfection for 48 h, cell lines were filtered by puromycin treatment (1 µg ml^−1^) for three days. Quantitative RT–PCR (qRT–PCR) and western blotting (WB) were applied to determine transfection efficiency at the mRNA and protein levels, respectively.

### qRT–PCR

Total RNAs from stably transfected cell lines were extracted using TRIzol reagent (Invitrogen) and reverse transcribed using the UeIris II RT‐PCR System for First‐Strand cDNA Synthesis (US Everbright, China) according to the manufacturer's protocol. qRT–PCR was conducted using the CFX Connect System (Bio‐Rad, USA), and SYBR Green Mix (US Everbright, China) was applied to determine mRNA expression levels. The mRNA expression of GAPDH was used to normalized and the relative expression of the target mRNAs was determined. Sangon Biotech (Shanghai, China) designed and synthesized the primers: Human CCL2, F‐TCGCGAGCTATAGAAGAATCA, R‐TGTTCAAGTCTTCGGAGTTTG; Human CCL3, F‐ATGCAGGTCTCCACTGCTGC, R‐TCAGGCACTCAGCTCCAGGTC; Human CCL4, F‐CCAAACCAAAAGAAGCAAGC, R‐ACAGTGGACCATCCCCATAG; Human CCL5, F‐GAGTATTTCTACACCAGTGGCAAG, R‐TCCCGAACCCATTTCTTCTCT; Human CXCL9, F‐GAGTGCAAGGAACCCCAGTAG, R‐GGTGGATAGTCCCTTGGTTGG; Human CXCL10, F‐TGGCATTCAAGGAGTACCTCTC, R‐GGACAAAATTGGCTTGCAGGA; Human S100A5, F‐CATATGCCTGCTGCTTGGATTCTC, R‐GGATCCTCACTTGTTGTCCTCTAGAAAG; Mouse S100A5, F‐GCAAGCTGACCCTGAGTAGG, R‐CGCTGTTTTTGTCCAGGCTC; Human GAPDH, F‐CTCAACTACATGGTCTACATGTTCCA, R‐CTTCCCATTCTCAGCCTTGACT; Mouse GAPDH, F‐ACCACAGTCCATGCCATCAC, R‐TCCACCACCCTGTTGCTGTA.

### Western Blotting (WB) Analysis

Cells were lysed with 100 µl RIPA buffer (NCM biotech, China) accompanied with 1 µl Phenylmethanesulfonyl fluoride (PMSF). The protein concentration was quantified using a BCA Protein Assay Kit (NCM Biotech, China). Polyvinylidene fluoride (PVDF) membranes were blocked for 1 h with 5% skim TBST solution at room temperature. PVDF was incubated at 4 °C overnight with primary antibodies: anti‐S100A5 (Cat: 17924‐1‐AP, Proteintech, USA) and anti‐GAPDH (Cat: ab8245, Abcam, USA). After washing with TBST for three times, the PVDF membrane was incubated at room temperature for 1 h with horseradish peroxidase‐conjugated secondary antibodies. Finally, the proteins were visualized with ECL system (Thermo Fisher Scientific).

### ProcartaPlex Multiple Immunoassays

Cell culture supernatants were collected from a 24‐well plate after centrifugation and detected multiple cytokines and chemokines (including: CCL2, CCL3, CCL4, CCL5, CCL11, CTLA‐8, CXCL1, CXCL8, CXCL10, CXCL12, GM‐CSF, IFN‐*α*, IFN‐*γ*, IL‐1 *α*, IL‐1 *β*, IL‐1RA, IL‐2, IL‐4, IL‐5, IL‐6, IL‐7, IL‐9, IL‐10, IL‐12, IL‐13, IL‐15, IL‐18, IL‐21, IL‐22, IL‐23, IL‐27, IL‐31, TNF‐*α*, TNF‐*β*) using EPX340‐12167‐901 kit and Luminex detection platform (ThermoFisher Scientific, Massachusetts, USA) according to manufacturer's instruction. After log2 transformation and scaling, the fluorescence intensity was presented in a heatmap.

### ELISA

Cell culture supernatants were collected from 24‐well plates, and the presence of the cytokine/chemokine proteins CCL2, CCL3, CCL4, CCL5, CXCL9, and CXCL10 was determined using a human ELISA kit (Proteintech, USA) and a Biotech microplate reader (ThermoFisher Scientific, USA) according to the manufacturer's instructions. The concentrations of different cytokine/chemokine proteins were calculated based on Optical Density (OD) values at a detection wavelength of 450 nm.

### T Cell‐Mediated Tumor Cell‐Killing Assay

Human peripheral blood mononuclear cells (PBMCs) were isolated from the peripheral blood of healthy donors by gradient centrifugation using Lymphoprep (Cat: 0 7851; StemCell Technologies, USA). Written informed consent was obtained from each donor. After removing red cells by Red Blood Cell Lysis Buffer (Solarbio, China), PBMCs were cultured in DMEM medium and activated to acquire T cells for one week using ImmunoCult Human CD3/CD28/CD2 T cell activator (25ul ml^−1^, Cat:10 970; STEMCELL Technologies, USA) and recombinant human IL‐2 (10 ng ml^−1^, Cat: 202‐IL‐050, R&D, USA). Transfected T24, 5637, and their negative control cell lines were cultured in 12‐well plates overnight for adhesion and then co‐cultured with activated T cells for 72 h in the presence of IL‐2 (10 ng mL^−1^) and anti‐CD3 antibody (100 ng ml^−1^, Thermo Scientific, USA). After washing out T and dead cells with PBS, crystal violet was used to stain the remaining adhered cancer cells, and a microplate reader was used to detect OD values at a detection wavelength of 570 nm. This study was approved by the Ethics Committee of Xiangya Hospital, Central South University (202104145).

### CD8^+^ T Cell Migration and Inhibition Assay

First, human CD8^+^ T cells were isolated from human PBMCs using Human CD8^+^ T Cell Isolation Kit (Cat: 480 012, Biolegend, USA) by magnetic bead separation and activated them with ImmunoCult Human CD3/CD28/CD2 T cell activator and recombinant human IL‐2. Then, CD8^+^ T cell migration assay was conducted using a 24‐well transwell system with 6.5 mm diameter and 3 µm pore size polycarbonate membrane (Corning, USA).^[^ ^43^ ^]^ 600 µL supernatant from different transfected T24 and 5637 cell lines was added to the lower chamber, while 1 × 10^5^ isolated CD8^+^ T cells were added to the upper chamber. The T cells migrated into the lower chamber were collected and counted using flow cytometry after 6 h incubation at 37 °C.

Murine CD8^+^ T cells were isolated from the spleens of C57BL/6 mice using the Mouse CD8 T Cell Isolation Kit (Cat: 480 035, Biolegend, USA) by magnetic bead separation and then activated with anti‐CD3 (plate‐coated, 5 µg mL^−1^) and anti‐CD28 (soluble, 5 µg mL^−1^). After activation, 5 × 10^5^ isolated murine CD8^+^ T cells were cultured with S100A5 Mouse Recombinant (15 µg mL^−1^; Cat: PRO‐1084, ProSpec‐Tany TechnoGene Ltd) or placebo for 48 h. The cells were blocked with a Monensin Solution (Cat: S1753, Beyotime, China) for 4 h before harvesting for flow cytometry.

### MTT Assay

Knockdown and overexpression of S100A5 and their negative control cells (5 × 10^3^ cells well^−1^) were seeded into 96‐well culture plate. 20 µl methylthiazolyldiphenyl‐tetrazolium bromide (MTT, 5 mg ml^−1^, Sigma‐Aldrich) was added to each well at 0, 24, 48 and 72 h after cell adhesion, and incubated for 4 h at 37 °C. Then 150 µl of dimethyl sulfoxide (DMSO) was added to each well for 5 min at room temperature. A microplate reader was used to detect the OD values at a detection wavelength of 490 nm.

### Colony Formation Assay

Knockdown and overexpression of S100A5 and their negative control cells (1 × 10^3^ cells well^−1^) were seeded in 6‐well culture plates. Then cells were cultured in an incubator at 37 °C for 8 days, and the medium was replaced every three days. Cells were washed with PBS for three times, fixed with formalin, and then stained with 0.1% crystal violet.

### Wound Healing Assay

Lines were drawn (width 1 cm) on the back of 6‐well culture plate, and 5 × 10^5^ cells were added to each well. When the cell confluency reached more than 80%, the back line was drawn perpendicular to the hole using the tip of the pipe. The scratched cells were washed three times with PBS. Fresh serum‐free media were added and incubated for 24 h at 37 °C. Images were captured using a microscope after three washes with PBS.

### Preparation and Characterization of Liposome@siS100A5

Sangon Biotech (Shanghai, China) designed and synthesized the siS100A5: sense‐ GUCACCACUUUCCAUAAAUAUTT, and antisense‐ AUAUUUAUGGAAAGUGGUGACTT. SPC, DOTAP, DSPE‐PEG‐2K, and cholesterol were dissolved in 2 mL absolute ethanol. siS100A5 was first dissolved in sodium citrate buffer solution (containing 25% ethanol at pH 4) and then slowly added to the lipids. siS100A5 and the lipids were mixed and incubated for 20 min. Finally, the unloaded siS100A5 was removed using a polycarbonate membrane with a 50 nm diameter to obtain liposome@siS100A5.

The morphology of the obtained liposome@siS100A5 was examined using a transmission electron microscope (Hitachi, HT7820, 120 kV, Japan). A Zetasizer Nano‐ZS instrument (ZEN3600 Malvern, UK) was used to measure the hydrodynamic diameter (DLS) and zeta potential of the liposome@siS100A5. Loading efficiency was calculated from the fluorescence spectra obtained using a fluorescence spectrometer (Hitachi F‐4600, Japan). All experiments were conducted according to the previous studies.^[^ ^44^, ^45^ ^]^

### In Vivo Experiments

Purchased from the Department of Laboratory Animals, Central South University, 28 C57BL/6 mice (female, 6 weeks) were randomly divided into four groups (n = 7 per group). Next, 5 × 10^5^ S100A5 KD (sh‐S100A5 group) and negative control (shNC group) MB49 cells were injected subcutaneously into the right flank of two groups of mice respectively. When tumor volume reached 100 mm^3^, 100 µg anti‐mouse PD‐1 (Cat: BE0146, Bioxcell, USA) and IgG2a isotype (Cat: BE0089, Bioxcell, USA) were injected intraperitoneally into sh‐S100A5 groups respectively (sh‐S100A5 + anti‐PD‐1 group and sh‐S100A5 + IgG2a isotype group). The same for shNC group, namely, the shNC + anti‐PD‐1 and shNC + IgG2a isotypes. The drugs were administered every three days for five cycles. The mice was killed by euthanasia and collected tumors two days after the last treatment cycle or on the day of tumor volume ≥ 2000 mm^3^, tumor diameter ≥ 2 cm, or on appearance of tumor ulceration. After tumor volume measurement, the tumors were ground physically, digested, and filtered by 70 µm cell strainers (BIOFIL, China) to obtain single cell suspension for flow cytometry analysis.

To determine the anti‐tumor efficiency of liposome@siS100A5, 5 × 10^5^ MB49 cells were injected subcutaneously into the right flank of the mice. When the tumor volume reached 100 mm^3^, the mice were administered drugs intravenously every three days for five cycles as follow: (1) liposome + IgG2a isotype, (2) liposome@siS100A5 + IgG2a isotype, (3) liposome + anti‐PD‐1, and (4) liposome@siS100A5 + anti‐PD‐1. The injection dose was 100 µg for IgG2a isotype or anti‐PD‐1 and 3.4 µg for siS100A5 in each mouse. On days 3, 7, and 14 after the first injection, an automated hematology analyzer (HF‐3800, HLife, China) and hematology chemistry analyzer (PointCare V2, MNChip, China) were used to test the mouse whole blood and blood biochemistry indexes, respectively. Two days after the last treatment cycle, mice were euthanized. Tumors were collected for flow cytometry analysis, and the heart, liver, spleen, lungs, and kidneys were collected for hematoxylin and eosin (H&E) staining. All experiments were approved by the Animal Care and Use Committee of Xiangya Hospital, Central South University (202104145).

### Flow Cytometry Analysis

The Zombie Aqua Fixable Viability Kit (Cat:423 102, BioLegend, USA) was used to remove dead cells, and single cells were blocked with anti‐mouse CD16/CD32 antibody (Cat:156 603, BioLegend, USA). Then, APC‐Fire 750 anti‐CD45 (Cat:103 153, BioLegend, USA), BV785 anti‐CD11b (Cat:101 243, BioLegend, USA), BV421 anti‐CD3 (Cat:100 227, BioLegend, USA), BV605 anti‐CD8a (Cat:100 743, BioLegend, USA), Super Bright 436 anti‐CD279 (PD‐1, Cat:62‐9985‐82, Thermo, USA) were used to stain cell members for 30 minutes. A transcription factor Staining Buffer Set (Cat:424 401, BioLegend, USA) was used to fix and permeabilize the cells. Intracellular markers were stained for 50 minutes with PE anti‐GZMB (Cat:372 207, BioLegend, USA), PE‐Dazzle 594 anti‐IFN‐*γ* (Cat:505 845, BioLegend, USA), PE‐CY7 anti‐TNF‐*α* (Cat:506 324, BioLegend, USA), and APC anti‐Perforin (Cat:154 303, BioLegend, USA). Cytek DxpAthena Flow cytometer (Cytek Biosciences, USA) was applied to detect stained cells, and FlowJo software (version 10.8.1) was used to analyze the data.

### Immunohistochemistry (IHC) and Immunofluorescence (IF)

TMAs were prepared according to the method reported by Matthew et al.^[^ ^46^ ^]^ and IHC was performed previously described.^[^ ^47^ ^]^ Multi‐color IF of the Xiangya immune cohort was performed using a multiple fluorescent IHC staining kit (Absin, China). Anti‐S100A5 (Cat: 17924‐1‐AP, Proteintech, USA) was used, anti‐CD8 (Cat: ab4055, Abcam, USA), and anti‐PD‐L1 (Cat: ab213524, Abcam, USA) antibodies. In addition, the IHC scoring of CD8, PD‐L1 was determined, and S100A5 using a combined system of 4‐point scale and the percentage of stained cells. No staining or absence of any stained cells was assigned a score of 0; weak staining (faint yellow), a score of 1; moderate staining (pale brown), a score of 2; and strong staining (brown), a score of 3. For the percentage of stained cells, samples with < 25% of stained cells were given a score of 1, those with 25–49% of stained cells were given a score of 2, those with 50–74% of stained cells were given a score of 3, and those with ≥ 75% of stained cells were given a score of 4. The intensity and percentage scores were then multiplied to obtain the protein expression score, which was termed the IHC score. The samples were divided into high S100A5 and low S100A5 groups based on the expression of S100A5 protein, with a median cutoff value of 6. Moreover, according to the previous study,^[^ ^17^ ^]^ the samples were defined as “inflamed phenotype” samples in which CD8^+^ T cells were located in the tumor parenchyma and defined the samples as “non‐inflamed phenotype” samples in which CD8^+^ T cells were located in the stroma (not in parenchyma) or no CD8^+^ T cells either in parenchyma or stroma. Two independent pathologists reviewed the TMAs. For conducting immunofluorescence (IF) of mouse tissues, an anti‐CD8 antibody (Cat: ab217344, Abcam, USA) was used.

### TissueFAXS Cytometry Panoramic Tissue Quantification Assay

The TissueFAXS Cytometry panoramic tissue quantification assay was conducted on the TMAs of 50 BLCA samples without prior treatment, as previously reported.^[^ ^48^ ^]^ One TMA sample was stained with anti‐S100A5 (Cat: 17924‐1‐AP, Proteintech, USA), anti‐CK19 (Cat: ab52625, Abcam, USA), anti‐CD8 (Cat: ab237709, Abcam), and anti‐CD4 (Cat: ab133616, Abcam) antibodies. Another TMA was stained with anti‐S100A5 (Cat: 17924‐1‐AP, Proteintech), anti‐CK19 (Cat: ab52625, Abcam), anti‐CD16 (Cat: 16559‐1‐AP, Proteintech), and anti‐CD68 (Cat: ab213363, Abcam) antibodies. Briefly, TMAs were removed residual paraffin and rehydrated using xylene and alcohol. Then TMAs were washed with ddH_2_O for 5 min, followed by microwave treatment. Afterward, the diluted primary antibody was applied to incubate at 37 °C for 2 h and then washed by PBS twice. TMAs were then incubated with the corresponding secondary antibody at room temperature for 30 min and washed again with PBS. These steps were repeated to complete the staining of the other three markers. Finally, the nuclear dye (SN470) was applied at room temperature for 5 min. TissueFAXS (TissueGnostics) with a Zeiss Axio Imager Z2 Microscope System at ×20 magnification was applied to acquire the images. For quantitative analysis, StrataQuest software (TissueGnostics) was applied to quantify the cell density of nucleus area per cell, expression per cell and area per cell. For spatial analysis, the numbers of CD4^+^ T and CD8^+^ T cells were quantified according to the distance gradients (0‐25 µm, 25–50 µm, 50–100 µm, and 100–150 µm) from S100A5^+^CK^+^ cells using StrataQuest software.

### Statistical Analysis

Pearson's or Spearman's coefficients were used to explore the correlations between variables. If the variables fit a normal distribution, a *t*‐test was used to analyze the differences between groups. Otherwise, the Mann‐Whitney U test was used. Differences between categorical variables were determined using Pearson's chi‐squared test or Fisher's exact test. K‐M analysis was used to plot the survival curves and the log‐rank test to determine the significance. *p* < 0.05 was set as a significant criterion. All statistical tests were two‐sided. All analyses were conducted using the R software (version 4.1.3) and GraphPad Prism (version 9.4.0).

## Conflict of Interest

The authors declare no conflict of interest.

## Author Contributions

LHH, LZH, CZY, CMF, OZY, and CJB performed main analyses and experiments and drafted the manuscript. LHH, ZCY, LZ, MM, TSY, WRZ, DDS and LJH searched and downloaded the original datasets from public databases and assisted performing experiments. LHH, LZH, CCL, LPH, CMF, OZY and HJ contributed to data collecting and statistical analyses. LHH, LZH, CCL, DDS, and LZ edited the pictures. HJ and ZXB conceived and supervised the study. All authors contributed to writing the manuscript. All authors reviewed and approved the final manuscript.

## Supporting information

Supporting Information

Supplemental Table 1

Supplemental Table 2

Supplemental Table 3

Supplemental Table 4

Supplemental Table 5

Supplemental Table 6

Supplemental Table 7

Supplemental Table 8

Supplemental Table 9

Supplemental Table 10

## Data Availability

The data that support the findings of this study are available from the corresponding author upon reasonable request.

## References

[1] H. Sung , J. Ferlay , R. L. Siegel , M. Laversanne , I. Soerjomataram , A. Jemal , F. Bray , Ca‐Cancer J. Clin. 2021, 71, 209. 33538338 10.3322/caac.21660

[2] V. G. Patel , W. K. Oh , M. D. Galsky , Ca‐Cancer J. Clin. 2020, 70, 404. 32767764 10.3322/caac.21631

[3] R. L. Siegel , K. D. Miller , A. Jemal , Cancer statistics 2018, 68, 7. 10.3322/caac.21442 29313949

[4] Nature 2014, 507, 315. 24476821 10.1038/nature12965 PMC3962515

[5] J. E. Rosenberg , J. Hoffman‐Censits , T. Powles , M. S. Van Der Heijden , A. V. Balar , A. Necchi , N. Dawson , P. H. O'donnell , A. Balmanoukian , Y. Loriot , S. Srinivas , M. M. Retz , P. Grivas , R. W. Joseph , M. D. Galsky , M. T. Fleming , D. P. Petrylak , J. L. Perez‐Gracia , H. A. Burris , D. Castellano , C. Canil , J. Bellmunt , D. Bajorin , D. Nickles , R. Bourgon , G. M. Frampton , N. Cui , S. Mariathasan , O. Abidoye , G. D. Fine , et al., Lancet (London, England) 2016, 387, 1909. 26952546 10.1016/S0140-6736(16)00561-4 PMC5480242

[6] P. Sharma , M. Retz , A. Siefker‐Radtke , A. Baron , A. Necchi , J. Bedke , E. R. Plimack , D. Vaena , M.‐O. Grimm , S. Bracarda , J. Á. Arranz , S. Pal , C. Ohyama , A. Saci , X. Qu , A. Lambert , S. Krishnan , A. Azrilevich , M. D. Galsky , Lancet Oncol. 2017, 18, 312. 28131785 10.1016/S1470-2045(17)30065-7

[7] D. S. Chen , I. Mellman , Nature 2017, 541, 321. 28102259 10.1038/nature21349

[8] Q. Duan , H. Zhang , J. Zheng , L. Zhang , Trends in cancer 2020, 6, 605. 32610070 10.1016/j.trecan.2020.02.022

[9] T. F. Gajewski , Semin. Oncol. 2015, 42, 663. 26320069 10.1053/j.seminoncol.2015.05.011 PMC4555998

[10] R. M. Zemek , E. De Jong , W. L. Chin , I. S. Schuster , V. S. Fear , T. H. Casey , C. Forbes , S. J. Dart , C. Leslie , A. Zaitouny , M. Small , L. Boon , A. R. R. Forrest , D. O. Muiri , M. A. Degli‐Esposti , M. J. Millward , A. K. Nowak , T. Lassmann , A. Bosco , R. A. Lake , W. J. Lesterhuis , Sci. Transl. Med. 2019, 11. 10.1126/scitranslmed.aav7816 31316010

[11] J. Larkin , V. Chiarion‐Sileni , R. Gonzalez , J. J. Grob , C. L. Cowey , C. D. Lao , D. Schadendorf , R. Dummer , M. Smylie , P. Rutkowski , P. F. Ferrucci , A. Hill , J. Wagstaff , M. S. Carlino , J. B. Haanen , M. Maio , I. Marquez‐Rodas , G. A. McArthur , P. A. Ascierto , G. V. Long , M. K. Callahan , M. A. Postow , K. Grossmann , M. Sznol , B. Dreno , L. Bastholt , A. Yang , L. M. Rollin , C. Horak , F. S. Hodi , et al., The New England journal of medicine 2015, 373, 23. 26027431 10.1056/NEJMoa1504030 PMC5698905

[12] A. R. Bresnick , D. J. Weber , D. B. Zimmer , Nat. Rev. Cancer 2015, 15, 96. 25614008 10.1038/nrc3893 PMC4369764

[13] M. W. Nasser , Z. Qamri , Y. S. Deol , J. Ravi , C. A. Powell , P. Trikha , R. A. Schwendener , X. F. Bai , K. Shilo , X. Zou , G. Leone , R. Wolf , S. H. Yuspa , R. K. Ganju , Cancer Res. 2012, 72, 604. 22158945 10.1158/0008-5472.CAN-11-0669 PMC3271140

[14] I. J. Bettum , K. Vasiliauskaite , V. Nygaard , T. Clancy , S. J. Pettersen , E. Tenstad , G. M. Mælandsmo , L. Prasmickaite , Cancer letters 2014, 344, 28. 24215866 10.1016/j.canlet.2013.10.036

[15] S. Hancq , I. Salmon , J. Brotchi , O. De Witte , H.‐J. Gabius , C. W. Heizmann , R. Kiss , C. Decaestecker , Neuropathology and applied neurobiology 2004, 30, 178. 15043715 10.1046/j.0305-1846.2003.00525.x

[16] R. Yao , A. Lopez‐Beltran , G. T. Maclennan , R. Montironi , J. N. Eble , L. Cheng , Anticancer Res. 2007, 27, 3051. 17970044

[17] J. Hu , A. Yu , B. Othmane , D. Qiu , H. Li , C. Li , P. Liu , W. Ren , M. Chen , G. Gong , X. Guo , H. Zhang , J. Chen , X. Zu , Theranostics 2021, 11, 3089. 33537076 10.7150/thno.53649 PMC7847675

[18] J. Hu , B. Othmane , A. Yu , H. Li , Z. Cai , X. Chen , W. Ren , J. Chen , X. Zu , BMC Med. 2021, 19, 289. 34836536 10.1186/s12916-021-02163-6 PMC8627095

[19] J. Hu , J. Chen , Z. Ou , H. Chen , Z. Liu , M. Chen , R. Zhang , A. Yu , R. Cao , E. Zhang , X. Guo , B. Peng , D. Deng , C. Cheng , J. Liu , H. Li , Y. Zou , R. Deng , G. Qin , W. Li , L. Wang , T. Chen , X. Pei , G. Gong , J. Tang , B. Othmane , Z. Cai , C. Zhang , Z. Liu , X. Zu , Cell reports Medicine 2022, 3, 100785. 36265483 10.1016/j.xcrm.2022.100785 PMC9729796

[20] M. J. Goldman , B. Craft , M. Hastie , K. Repečka , F. McDade , A. Kamath , A. Banerjee , Y. Luo , D. Rogers , A. N. Brooks , J. Zhu , D. Haussler , Nat. Biotechnol. 2020, 38, 675. 32444850 10.1038/s41587-020-0546-8 PMC7386072

[21] S. Mariathasan , S. J. Turley , D. Nickles , A. Castiglioni , K. Yuen , Y. Wang , E. E. Kadel III , H. Koeppen , J. L. Astarita , R. Cubas , S. Jhunjhunwala , R. Banchereau , Y. Yang , Y. Guan , C. Chalouni , J. Ziai , Y. Şenbabaoğlu , S. Santoro , D. Sheinson , J. Hung , J. M. Giltnane , A. A. Pierce , K. Mesh , S. Lianoglou , J. Riegler , R. A. D. Carano , P. Eriksson , M. Höglund , L. Somarriba , D. L. Halligan , et al., Nature 2018, 554, 544. 29443960 10.1038/nature25501 PMC6028240

[22] L. Xu , C. Deng , B. Pang , X. Zhang , W. Liu , G. Liao , H. Yuan , P. Cheng , F. Li , Z. Long , M. Yan , T. Zhao , Y. Xiao , X. Li , Cancer Res. 2018, 78, 6575. 30154154 10.1158/0008-5472.CAN-18-0689

[23] P. Charoentong , F. Finotello , M. Angelova , C. Mayer , M. Efremova , D. Rieder , H. Hackl , Z. Trajanoski , Cell Rep. 2017, 18, 248. 28052254 10.1016/j.celrep.2016.12.019

[24] E. Becht , N. A. Giraldo , L. Lacroix , B. Buttard , N. Elarouci , F. Petitprez , J. Selves , P. Laurent‐Puig , C. Sautès‐Fridman , W. H. Fridman , A. De Reyniès , Genome Biol. 2016, 17, 218. 27765066 10.1186/s13059-016-1070-5 PMC5073889

[25] N. Auslander , G. Zhang , J. S. Lee , D. T. Frederick , B. Miao , T. Moll , T. Tian , Z. Wei , S. Madan , R. J. Sullivan , G. Boland , K. Flaherty , M. Herlyn , E. Ruppin , Nat. Med. 2018, 24, 1545. 30127394 10.1038/s41591-018-0157-9 PMC6693632

[26] M. Ayers , J. Lunceford , M. Nebozhyn , E. Murphy , A. Loboda , D. R. Kaufman , A. Albright , J. D. Cheng , S. P. Kang , V. Shankaran , S. A. Piha‐Paul , J. Yearley , T. Y. Seiwert , A. Ribas , T. K. Mcclanahan , J. Clin. Invest. 2017, 127, 2930. 28650338 10.1172/JCI91190 PMC5531419

[27] Z. Chen , L. Zhou , L. Liu , Y. Hou , M. Xiong , Y. Yang , J. Hu , K. Chen , Nat. Commun. 2020, 11, 5077. 33033240 10.1038/s41467-020-18916-5 PMC7545162

[28] H. Lai , X. Cheng , Q. Liu , W. Luo , M. Liu , M. Zhang , J. Miao , Z. Ji , G. N. Lin , W. Song , L. Zhang , J. Bo , G. Yang , J. Wang , W.‐Q. Gao , Int. J. Cancer 2021, 149, 2099. 34480339 10.1002/ijc.33794

[29] H. W. Lee , W. Chung , H.‐O. Lee , D. E. Jeong , A. Jo , J. E. Lim , J. H. Hong , D.‐H. Nam , B. C. Jeong , S. H. Park , K.‐M. Joo , W.‐Y. Park , Genome Med 2020, 12, 47. 32460812 10.1186/s13073-020-00741-6 PMC7251908

[30] L. Wang , R. P. Sebra , J. P. Sfakianos , K. Allette , W. Wang , S. Yoo , N. Bhardwaj , E. E. Schadt , X. Yao , M. D. Galsky , J. Zhu , Genome Med 2020, 12, 24. 32111252 10.1186/s13073-020-0720-0 PMC7049190

[31] K. C. Yuen , L. F. Liu , V. Gupta , S. Madireddi , S. Keerthivasan , C. Li , D. Rishipathak , P. Williams , E. E. 3rd Kadel , H. Koeppen , Y. J. Chen , Z. Modrusan , J. L. Grogan , R. Banchereau , N. Leng , A. Thastrom , X. Shen , K. Hashimoto , D. Tayama , M. S. van der Heijden , J. E. Rosenberg , D. F. McDermott , T. Powles , P. S. Hegde , M. A. Huseni , S. Mariathasan , Nat. Med. 2020, 26, 693. 32405063 10.1038/s41591-020-0860-1 PMC8286544

[32] J. Peng , B.‐F. Sun , C.‐Y. Chen , J.‐Y. Zhou , Y.‐S. Chen , H. Chen , L. Liu , D. Huang , J. Jiang , G.‐S. Cui , Y. Yang , W. Wang , D. Guo , M. Dai , J. Guo , T. Zhang , Q. Liao , Y. Liu , Y.‐L. Zhao , D.‐L. Han , Y. Zhao , Y.‐G. Yang , W. Wu , Cell Res. 2019, 29, 725. 31273297 10.1038/s41422-019-0195-y PMC6796938

[33] S. Jin , C. F. Guerrero‐Juarez , L. Zhang , I. Chang , R. Ramos , C.‐H. Kuan , P. Myung , M. V. Plikus , Q. Nie , Nat. Commun. 2021, 12, 1088. 33597522 10.1038/s41467-021-21246-9 PMC7889871

[34] A. Liberzon , C. Birger , H. Thorvaldsdóttir , M. Ghandi , J. P. Mesirov , P. Tamayo , Cell systems 2015, 1, 417. 26771021 10.1016/j.cels.2015.12.004 PMC4707969

[35] A. Kamoun , A. de Reyniès , Y. Allory , G. Sjödahl , A. G. Robertson , R. Seiler , K. A. Hoadley , C. S. Groeneveld , H. Al‐Ahmadie , W. Choi , M. A. A. Castro , J. Fontugne , P. Eriksson , Q. Mo , J. Kardos , A. Zlotta , A. Hartmann , C. P. Dinney , J. Bellmunt , T. Powles , N. Malats , K. S. Chan , W. Y. Kim , D. J. McConkey , P. C. Black , L. Dyrskjøt , M. Höglund , S. P. Lerner , F. X. Real , F. Radvanyi , European urology 2020, 77, 420. 31563503 10.1016/j.eururo.2019.09.006 PMC7690647

[36] S. Hänzelmann , R. Castelo , J. Guinney , BMC Bioinformatics 2013, 14, 7. 23323831 10.1186/1471-2105-14-7 PMC3618321

[37] S. Rebouissou , I. Bernard‐Pierrot , A. De Reyniès , M.‐L. Lepage , C. Krucker , E. Chapeaublanc , A. Hérault , A. Kamoun , A. Caillault , E. Letouzé , N. Elarouci , Y. Neuzillet , Y. Denoux , V. Molinié , D. Vordos , A. Laplanche , P. Maillé , P. Soyeux , K. Ofualuka , F. Reyal , A. Biton , M. Sibony , X. Paoletti , J. Southgate , S. Benhamou , T. Lebret , Y. Allory , F. Radvanyi , Sci. Transl. Med. 2014, 6, 244ra291. 10.1126/scitranslmed.3008970 25009231

[38] N.‐A.‐D. Marzouka , P. Eriksson , C. Rovira , F. Liedberg , G. Sjödahl , M. Höglund , Sci. Rep. 2018, 8, 3737. 29487377 10.1038/s41598-018-22126-x PMC5829240

[39] W. Choi , S. Porten , S. Kim , D. Willis , E. R. Plimack , J. Hoffman‐Censits , B. Roth , T. Cheng , M. Tran , I.‐L. Lee , J. Melquist , J. Bondaruk , T. Majewski , S. Zhang , S. Pretzsch , K. Baggerly , A. Siefker‐Radtke , B. Czerniak , C. P. N. Dinney , D. J. Mcconkey , Cancer Cell 2014, 25, 152. 24525232 10.1016/j.ccr.2014.01.009 PMC4011497

[40] A. G. Robertson , J. Kim , H. Al‐Ahmadie , J. Bellmunt , G. Guo , A. D. Cherniack , T. Hinoue , P. W. Laird , K. A. Hoadley , R. Akbani , M. A. A. Castro , E. A. Gibb , R. S. Kanchi , D. A. Gordenin , S. A. Shukla , F. Sanchez‐Vega , D. E. Hansel , B. A. Czerniak , V. E. Reuter , X. Su , B. de Sa Carvalho , V. S. Chagas , K. L. Mungall , S. Sadeghi , C. S. Pedamallu , Y. Lu , L. J. Klimczak , J. Zhang , C. Choo , A. I. Ojesina , et al., Cell 2017, 171, 540.e525.28988769

[41] Q. Mo , F. Nikolos , F. Chen , Z. Tramel , Y.‐C. Lee , K. Hayashi , J. Xiao , J. Shen , K. S. Chan , Journal of the National Cancer Institute 2018, 110, 448. 29342309 10.1093/jnci/djx243 PMC6279371

[42] J. S. Damrauer , K. A. Hoadley , D. D. Chism , C. Fan , C. J. Tiganelli , S. E. Wobker , J. J. Yeh , M. I. Milowsky , G. Iyer , J. S. Parker , W. Y. Kim , Intrinsic subtypes of high‐grade bladder cancer reflect the hallmarks of breast cancer biology , Proceedings of the National Academy of Sciences of the United States of America, 2014, 111, pp. 3110–3115. 10.1073/pnas.1318376111 PMC3939870 24520177

[43] N. Nagarsheth , D. Peng , I. Kryczek , K. Wu , W. Li , E. Zhao , L. Zhao , S. Wei , T. Frankel , L. Vatan , W. Szeliga , Y. Dou , S. Owens , V. Marquez , K. Tao , E. Huang , G. Wang , W. Zou , Cancer Res. 2016, 76, 275. 26567139 10.1158/0008-5472.CAN-15-1938 PMC4715964

[44] S. Zhao , H. Li , R. Liu , N. Tao , L. Deng , Q. Xu , J. Hou , J. Sheng , J. Zheng , L. Wang , W. Chen , S. Guo , Y.‐N. Liu , J. Am. Chem. Soc. 2023, 145, 10322. 37097216 10.1021/jacs.3c02005

[45] X. Wu , M. Wen , Y. Zou , X. Gao , C. Wei , R. Liu , J. Li , L. Wang , X. Li , Y.‐N. Liu , W. Chen , Chem. Sci. 2022, 13, 6842. 35774154 10.1039/d2sc01894b PMC9200116

[46] M. Koo , J. M. Squires , D. Ying , J. Huang , Methods in molecular biology (Clifton, NJ) 2019, 1897, 313. 10.1007/978-1-4939-8935-5_27 30539455

[47] J. Hu , H. Li , T. He , H. Deng , G. Gong , Y. Cui , P. Liu , W. Ren , C. Li , J. Chen , X. Zu , Urologic oncology 2020, 38, 641.e619. 10.1016/j.urolonc.2020.04.015 32389428

[48] P. T. Nghiem , S. Bhatia , E. J. Lipson , R. R. Kudchadkar , N. J. Miller , L. Annamalai , S. Berry , E. K. Chartash , A. Daud , S. P. Fling , P. A. Friedlander , H. M. Kluger , H. E. Kohrt , L. Lundgren , K. Margolin , A. Mitchell , T. Olencki , D. M. Pardoll , S. A. Reddy , E. M. Shantha , W. H. Sharfman , E. Sharon , L. R. Shemanski , M. M. Shinohara , J. C. Sunshine , J. M. Taube , J. A. Thompson , S. M. Townson , J. H. Yearley , S. L. Topalian , et al., The New England journal of medicine 2016, 374, 2542. 27093365 10.1056/NEJMoa1603702 PMC4927341

[49] M. F. Sanmamed , L. Chen , Cell 2018, 175, 313. 30290139 10.1016/j.cell.2018.09.035 PMC6538253

[50] D. S. Chen , I. Mellman , Immunity 2013, 39, 1. 23890059 10.1016/j.immuni.2013.07.012

[51] Y. Zhu , X. An , X. Zhang , Y. Qiao , T. Zheng , X. Li , Molecular cancer 2019, 18, 152. 31679519 10.1186/s12943-019-1087-y PMC6827255

[52] T. F. Gajewski , L. Corrales , J. Williams , B. Horton , A. Sivan , S. Spranger , Adv. Exp. Med. Biol. 2017, 1036, 19. 29275462 10.1007/978-3-319-67577-0_2 PMC6693322

[53] R. Reschke , T. F. Gajewski , Science immunology 2022, 7, eabq6509. 35867802 10.1126/sciimmunol.abq6509

[54] Q. Gao , S. Wang , X. Chen , S. Cheng , Z. Zhang , F. Li , L. Huang , Y. Yang , B. Zhou , D. Yue , D. Wang , L. Cao , N. R. Maimela , B. Zhang , J. Yu , L. Wang , Y. Zhang , Journal for immunotherapy of cancer 2019, 7, 42. 30744691 10.1186/s40425-019-0511-6 PMC6371476

[55] Z. Long , C. Sun , M. Tang , Y. Wang , J. Ma , J. Yu , J. Wei , J. Ma , B. Wang , Q. Xie , J. Wen , Cell Discov. 2022, 8, 68. 35853872 10.1038/s41421-022-00415-0 PMC9296597

[56] M. Sade‐Feldman , K. Yizhak , S. L. Bjorgaard , J. P. Ray , C. G. de Boer , R. W. Jenkins , D. J. Lieb , J. H. Chen , D. T. Frederick , M. Barzily‐Rokni , S. S. Freeman , A. Reuben , P. J. Hoover , A. C. Villani , E. Ivanova , A. Portell , P. H. Lizotte , A. R. Aref , J. P. Eliane , M. R. Hammond , H. Vitzthum , S. M. Blackmon , B. Li , V. Gopalakrishnan , S. M. Reddy , Z. A. Cooper , C. P. Paweletz , D. A. Barbie , A. Stemmer‐Rachamimov , K. T. Flaherty , et al., Cell 2018, 175, 998.e1020. 30388456 10.1016/j.cell.2018.10.038 PMC6641984

[57] N. Wang , M. Chen , T. Wang , Journal of controlled release : official journal of the Controlled Release Society 2019, 303, 130. 31022431 10.1016/j.jconrel.2019.04.025 PMC7111479

[58] X. Cheng , J. Gao , Y. Ding , Y. Lu , Q. Wei , D. Cui , J. Fan , X. Li , E. Zhu , Y. Lu , Q. Wu , L. Li , W. Huang , Adv. Sci. (Weinh) 2021, 8, e2100876. 34085415 10.1002/advs.202100876 PMC8373168

[59] B. Kim , J.‐H. Park , M. J. Sailor , Adv. Mater. (Deerfield Beach, Fla) 2019, 31, 1903637. 10.1002/adma.201903637 PMC6891135 31566258

[60] C. Allgöwer , A.‐L. Kretz , S. Von Karstedt , M. Wittau , D. Henne‐Bruns , J. Lemke , Cancers 2020, 12, 2037. 32722137 10.3390/cancers12082037 PMC7465620

[61] C. Haase‐Kohn , S. Wolf , J. Lenk , J. Pietzsch , Biochem. Biophys. Res. Commun. 2011, 413, 494. 21924240 10.1016/j.bbrc.2011.08.132

[62] R. L. Stewart , B. L. Carpenter , D. S. West , T. Knifley , L. Liu , C. Wang , H. L. Weiss , T. S. Gal , E. B. Durbin , S. M. Arnold , K. L. O'connor , M. Chen , Oncotarget 2016, 7, 34630. 27127879 10.18632/oncotarget.8969 PMC5085181

[63] S. Hou , T. Tian , D. Qi , K. Sun , Q. Yuan , Z. Wang , Z. Qin , Z. Wu , Z. Chen , J. Zhang , Cell Death Dis. 2018, 9, 277. 29449540 10.1038/s41419-018-0319-1 PMC5833421

[64] C. Liu , S. Zheng , Z. Lu , Z. Wang , S. Wang , X. Feng , Y. Wang , N. Sun , J. He , Signal transduction and targeted therapy 2022, 7, 368. 36266269 10.1038/s41392-022-01196-4 PMC9584899

[65] P. L. Crispen , S. Kusmartsev , Cancer immunology, immunotherapy : CII 2020, 69, 3. 31811337 10.1007/s00262-019-02443-4 PMC6949323

[66] U. Sack , W. Walther , D. Scudiero , M. Selby , D. Kobelt , M. Lemm , I. Fichtner , P. M. Schlag , R. H. Shoemaker , U. Stein , Journal of the National Cancer Institute 2011, 103, 1018. 21685359 10.1093/jnci/djr190

[67] S. Burock , S. Daum , U. Keilholz , K. Neumann , W. Walther , U. Stein , BMC Cancer 2018, 18, 297. 29544454 10.1186/s12885-018-4197-9 PMC5856000

