# Supplementary material for: S100A5 Attenuates Efficiency of Anti‐PD‐L1/PD‐1 Immunotherapy by Inhibiting CD8+ T Cell‐Mediated Anti‐Cancer Immunity in Bladder Carcinoma
Source: Adv Sci (Weinh). 2023 Jul 6;10(25):2300110. doi: 10.1002/advs.202300110 (PMC10477882; doi:10.1002/advs.202300110)
Supplement: Supplementary file 1 — Supporting Information [file ADVS-10-2300110-s004.pdf]

ADVANCED SCIENCE
Open Access

Supporting Information

for *Adv. Sci.*, DOI 10.1002/advs.202300110

S100A5 Attenuates Efficiency of Anti-PD-L1/PD-1 Immunotherapy by Inhibiting $CD8^+$ T Cell-Mediated Anti-Cancer Immunity in Bladder Carcinoma

Huihuang Li, Jinbo Chen, Zhenghao Li, Minfeng Chen, Zhenyu Ou, Miao Mo, Ruizhe Wang, Shiyu Tong, Peihua Liu, Zhiyong Cai, Chunyu Zhang, Zhi Liu, Dingshan Deng, Jinhui Liu, Chunliang Cheng, Jiao Hu and Xiongbing Zu**

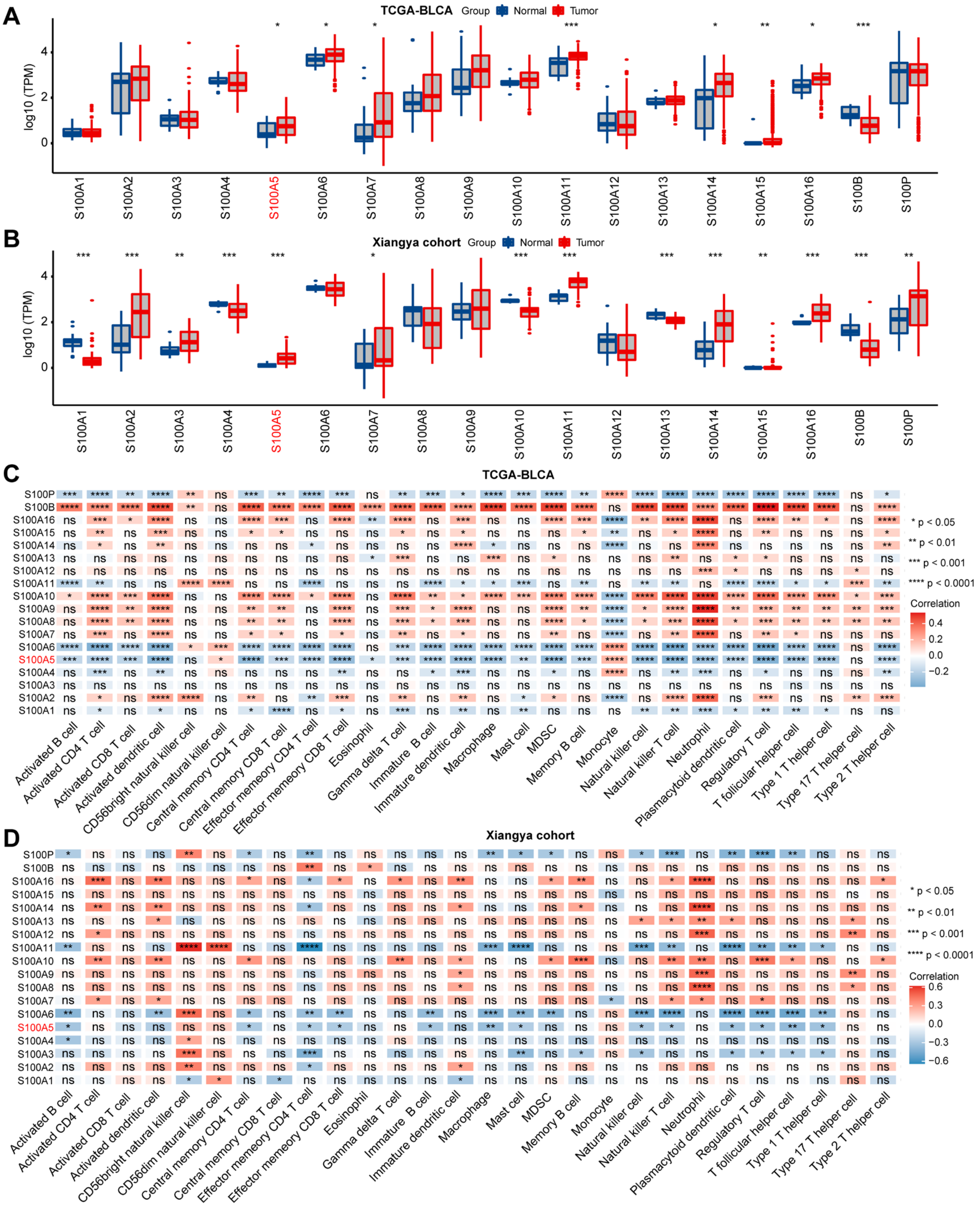

Figure S1. The expression pattern and immunological role of multiple S100 proteins in bladder carcinoma (BLCA). (A-B) The expression patterns of multiple S100 proteins in BLCA based on TCGA-BLCA (A) and Xiangya BLCA (B) cohorts respectively. (C-D) Correlation between multiple S100 proteins and 28 immune cells infiltration in BLCA based on TCGA-BLCA (C) and Xiangya BLCA (D) cohorts respectively. ns, not statistically significant; **P* < 0.05; ***P* < 0.01; ****P* < 0.001.

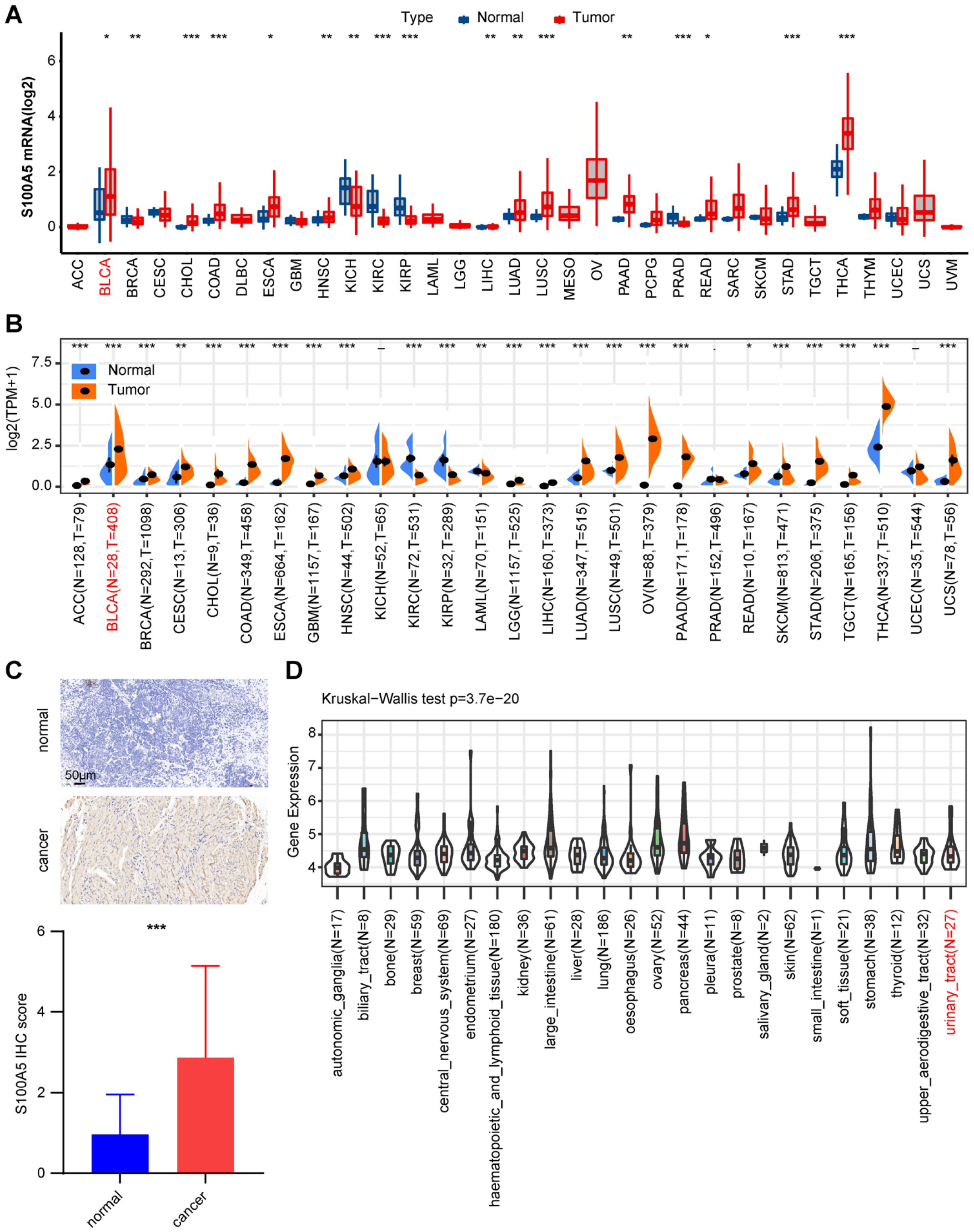

Figure S2. The expression pattern of S100A5 in multiple carcinoma types. (A-B) The expression patterns of S100A5 in TCGA (A) and TCGA combined with GTEx databases (B) across 33 types of carcinomas. (C) Representative images and boxplot of S100A5 IHC score in normal and tumor tissues. (D) The expression patterns of S100A5 in multiple kinds of normal tissues. $*P < 0.05$; $**P < 0.01$; $***P < 0.001$.

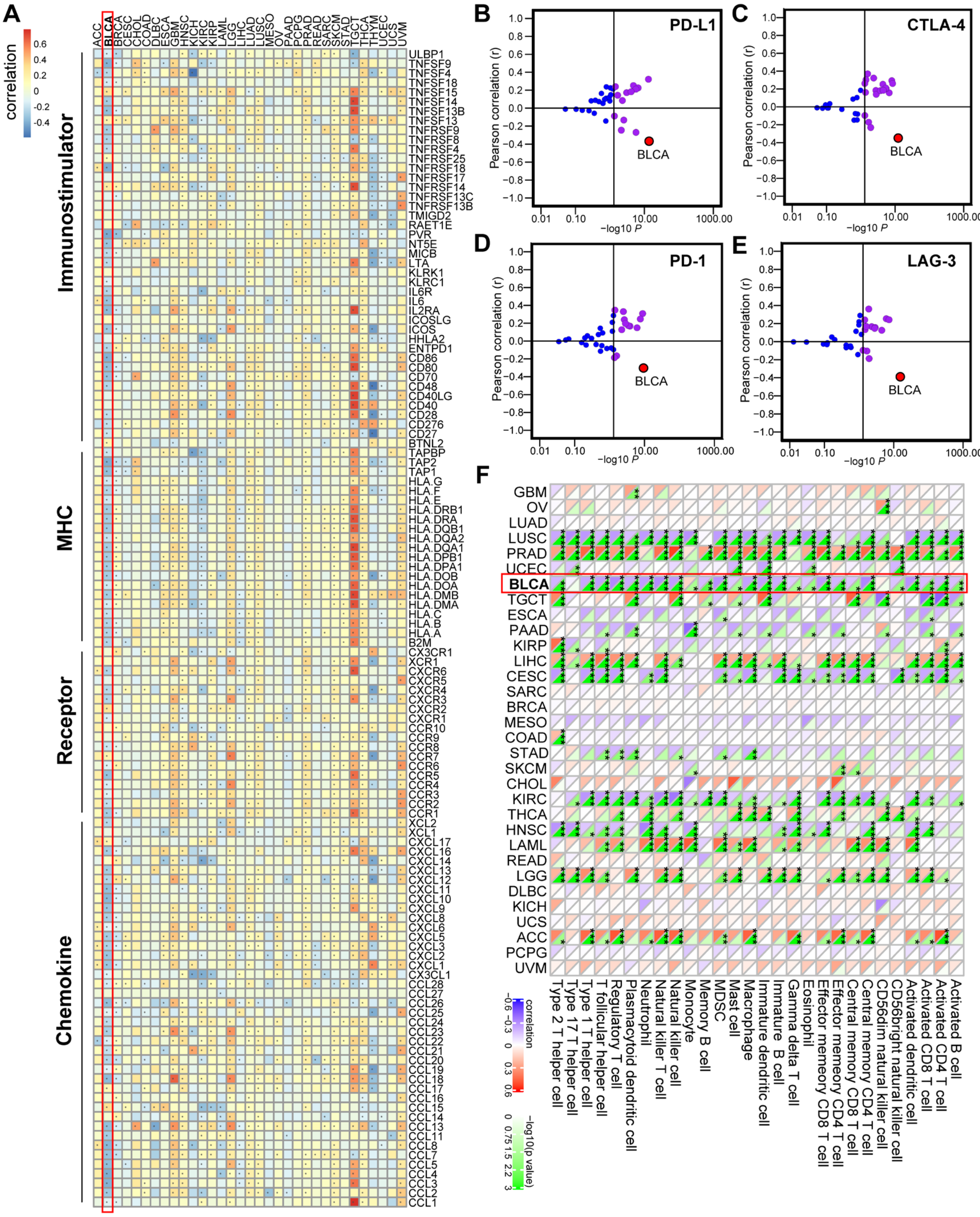

Figure S3. Immunological role of S100A5 in multiple carcinomas. (A) Different expression patterns of immune-stimulators, MHC, receptor, and chemokine between high and low S100A5 group; Red represents higher expressed genes, while blue represents lower expressed genes. (B-E) Correlation between S100A5 and programmed death-ligand 1 (PD-L1), cytotoxic T lymphocyte antigen (CTLA)-4, programmed death 1 (PD-1), and lymphocyte activation gene-3 (LAG-3). (F) Correlation between S100A5 and tumor-infiltrating immune cells in multiple carcinomas using the single-sample gene set enrichment analysis (ssGSEA) algorithm. Red, positive correlation; Blue,

negative correlation. *$P < 0.05$; **$P < 0.01$; ***$P < 0.001$.

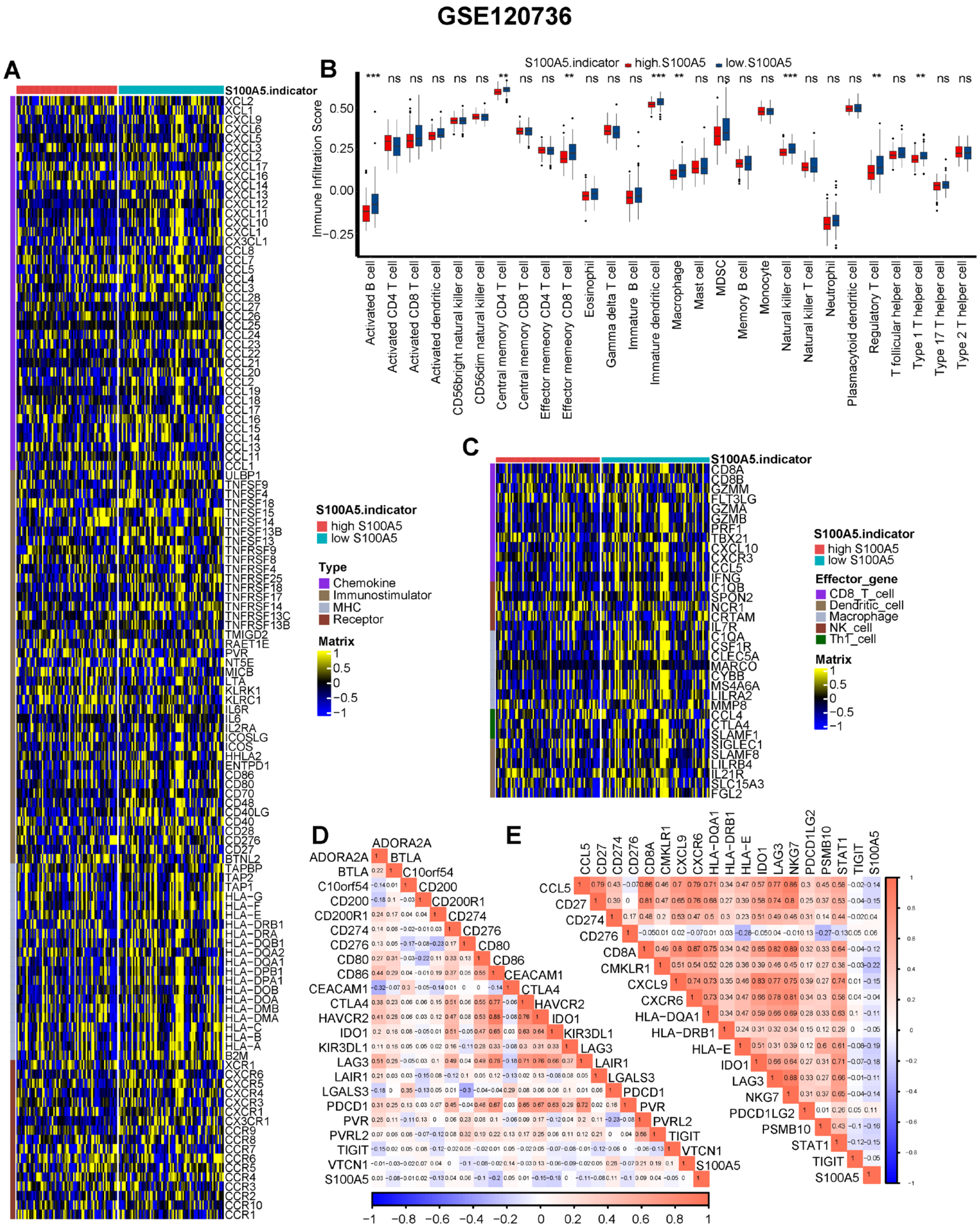

GSE31684

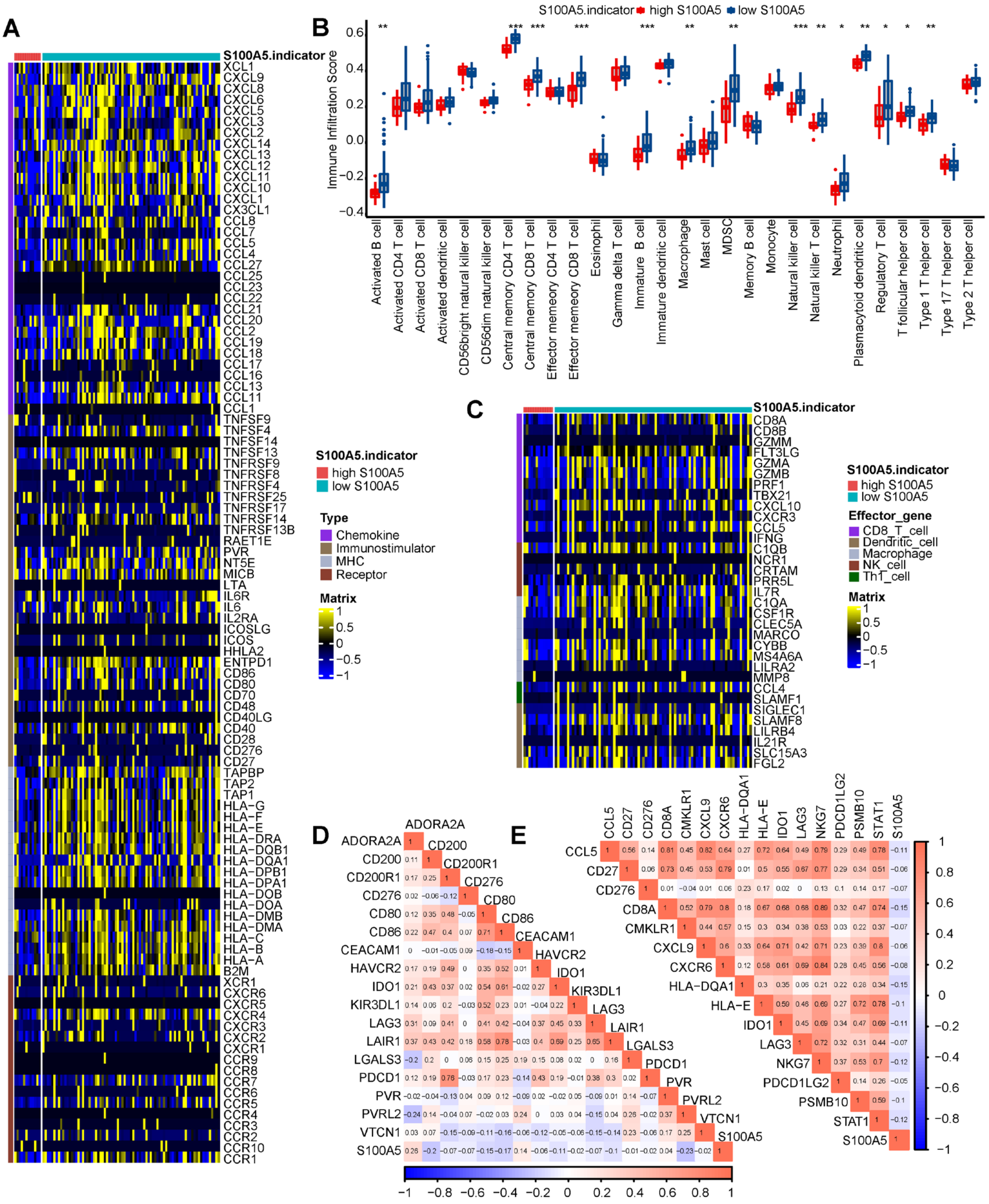
A
S100A5.indicator
XCL1
CXCL9
CXCL8
CXCL6
CXCL5
CXCL3
CXCL2
CXCL14
CXCL13
CXCL12
CXCL11
CXCL10
CXCL1
CX3CL1
CCL8
CCL7
CCL5
CCL4
CCL27
CCL25
CCL23
CCL22
CCL21
CCL20
CCL2
CCL19
CCL18
CCL17
CCL16
CCL13
CCL11
CCL1
TNFSF9
TNFSF4
TNFSF14
TNFSF13
TNFRSF9
TNFRSF8
TNFRSF4
TNFRSF25
TNFRSF17
TNFRSF14
TNFRSF13B
RAET1E
PVR
NT5E
MICB
LTA
IL6R
IL6
IL2RA
ICOSLG
ICOS
HHLA2
ENTPD1
CD86
CD80
CD70
CD48
CD40LG
CD40
CD28
CD276
CD27
TAPBP
TAP2
TAP1
HLA-G
HLA-F
HLA-E
HLA-DRA
HLA-DQB1
HLA-DQA1
HLA-DPB1
HLA-DPA1
HLA-DOB
HLA-DOA
HLA-DMB
HLA-DMA
HLA-C
HLA-B
HLA-A
B2M
XCR1
CXCR6
CXCR5
CXCR4
CXCR3
CXCR2
CXCR1
CCR9
CCR8
CCR7
CCR6
CCR5
CCR4
CCR3
CCR2
CCR10
CCR1
S100A5.indicator
high S100A5
low S100A5
Type
Chemokine
Immunostimulator
MHC
Receptor
Matrix
1
0.5
0
−0.5
−1
B
S100A5.indicator high S100A5 low S100A5
Immune Infiltration Score
0.6
0.4
0.2
0.0
−0.2
−0.4
Activated B cell
Activated CD4 T cell
Activated CD8 T cell
Activated dendritic cell
CD56bright natural killer cell
CD56dim natural killer cell
Central memory CD4 T cell
Central memory CD8 T cell
Effector memeory CD4 T cell
Effector memeory CD8 T cell
Eosinophil
Gamma delta T cell
Immature B cell
Immature dendritic cell
Macrophage
Mast cell
MDSC
Memory B cell
Monocyte
Natural killer cell
Natural killer T cell
Neutrophil
Plasmacytoid dendritic cell
Regulatory T cell
T follicular helper cell
Type 1 T helper cell
Type 17 T helper cell
Type 2 T helper cell
C
S100A5.indicator
CD8A
CD8B
GZMM
FLT3LG
GZMA
GZMB
PRF1
TBX21
CXCL10
CXCR3
CCL5
IFNG
C1QB
NCR1
CRTAM
PRR5L
IL7R
C1QA
CSF1R
CLEC5A
MARCO
CYBB
MS4A6A
LILRA2
MMP8
CCL4
SLAMF1
SIGLEC1
SLAMF8
LILRB4
IL21R
SLC15A3
FGL2
S100A5.indicator
high S100A5
low S100A5
Effector_gene
CD8_T_cell
Dendritic_cell
Macrophage
NK_cell
Th1_cell
Matrix
1
0.5
0
−0.5
−1
D
ADORA2A
CD200
CD200R1
CD276
CD80
CD86
CEACAM1
HAVCR2
IDO1
KIR3DL1
LAG3
LAIR1
LGALS3
PDCD1
PVR
PVRL2
VTCN1
S100A5
−1 −0.8 −0.6 −0.4 −0.2 0 0.2 0.4 0.6 0.8 1
E
CCL5
CD27
CD276
CD8A
CMKLR1
CXCL9
CXCR6
HLA−DQA1
HLA−E
IDO1
LAG3
NKG7
PDCD1LG2
PSMB10
STAT1
S100A5
1
0.8
0.6
0.4
0.2
0
−0.2
−0.4
−0.6
−0.8
−1

GSE32894

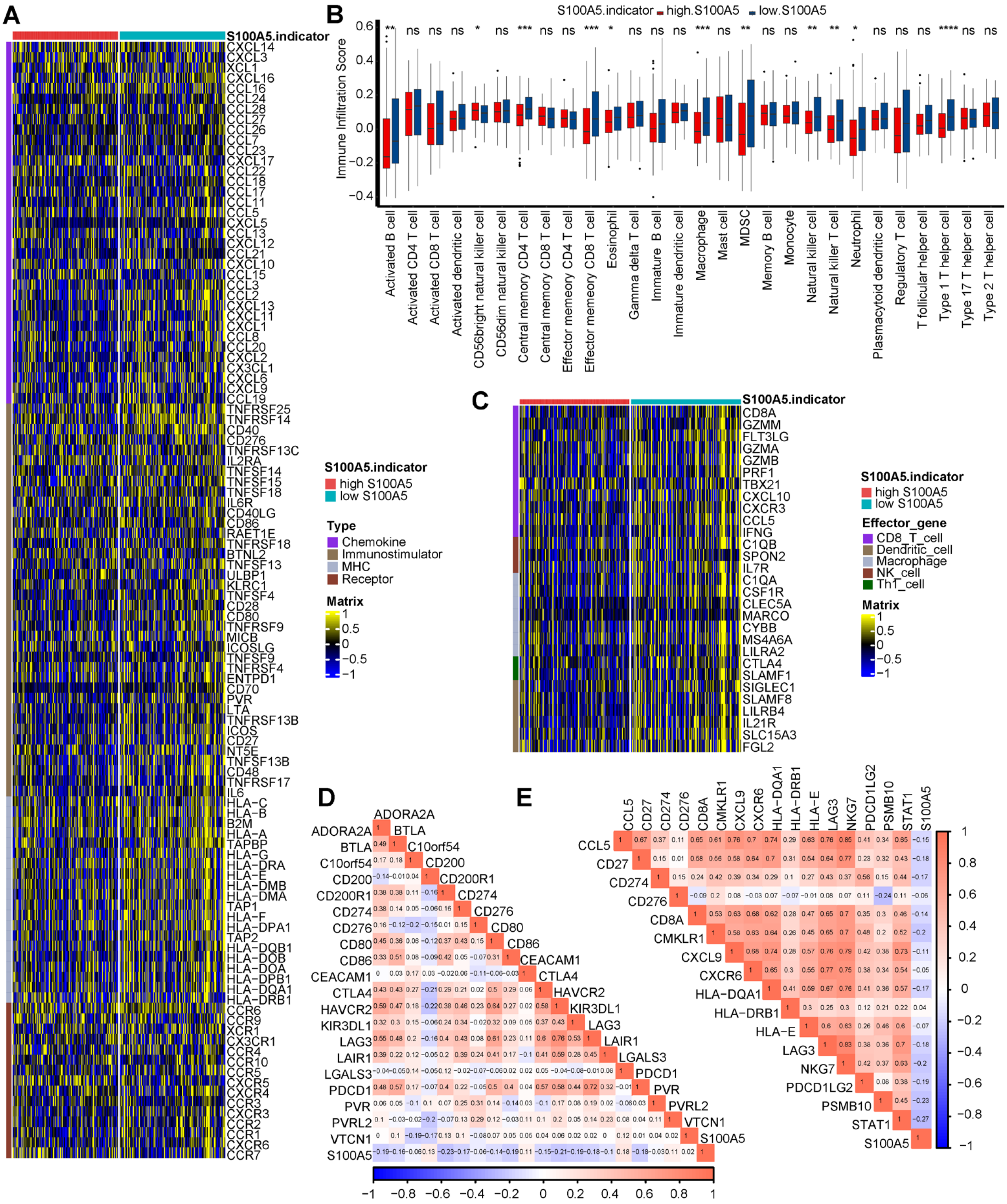
A
S100A5.indicator
CXCL14
CXCL3
XCL1
CXCL16
CCL16
CCL24
CCL28
CCL27
CCL26
CCL7
CCL23
CXCL17
CCL22
CCL18
CCL17
CCL11
CCL5
CXCL5
CCL13
CXCL12
CCL21
CXCL10
CCL15
CCL3
CCL2
CXCL13
CXCL11
CXCL1
CCL8
CCL20
CXCL2
CX3CL1
CXCL6
CXCL9
CCL19
TNFRSF25
TNFRSF14
CD40
CD276
TNFRSF13C
IL2RA
TNFSF14
TNFSF15
TNFSF18
IL6R
CD40LG
CD86
RAET1E
TNFRSF18
BTNL2
TNFSF13
ULBP1
KLRC1
TNFSF4
CD28
CD80
TNFRSF9
MICB
ICOSLG
TNFSF9
TNFRSF4
ENTPD1
CD70
PVR
LTA
TNFRSF13B
ICOS
CD27
NT5E
TNFSF13B
CD48
TNFRSF17
IL6
HLA-C
HLA-B
B2M
HLA-A
TAPBP
HLA-G
HLA-DRA
HLA-E
HLA-DMB
HLA-DMA
TAP1
HLA-F
HLA-DPA1
TAP2
HLA-DQB1
HLA-DOB
HLA-DOA
HLA-DPB1
HLA-DQA1
HLA-DRB1
CCR6
CCR9
XCR1
CX3CR1
CCR4
CCR10
CCR5
CXCR5
CXCR4
CCR3
CXCR3
CCR2
CCR1
CXCR6
CCR7
S100A5.indicator
high S100A5
low S100A5
Type
Chemokine
Immunostimulator
MHC
Receptor
Matrix
1
0.5
0
−0.5
−1
B
S100A5.indicator high.S100A5 low.S100A5
Immune Infiltration Score
0.6
0.4
0.2
0.0
−0.2
−0.4
** ns ns ns * ns *** ns ns *** * ns ns ns *** ns ** ns ns ** ** * ns ns ns **** ns ns
Activated B cell
Activated CD4 T cell
Activated CD8 T cell
Activated dendritic cell
CD56bright natural killer cell
CD56dim natural killer cell
Central memory CD4 T cell
Central memory CD8 T cell
Effector memeory CD4 T cell
Effector memeory CD8 T cell
Eosinophil
Gamma delta T cell
Immature B cell
Immature dendritic cell
Macrophage
Mast cell
MDSC
Memory B cell
Monocyte
Natural killer cell
Natural killer T cell
Neutrophil
Plasmacytoid dendritic cell
Regulatory T cell
T follicular helper cell
Type 1 T helper cell
Type 17 T helper cell
Type 2 T helper cell
C
S100A5.indicator
CD8A
GZMM
FLT3LG
GZMA
GZMB
PRF1
TBX21
CXCL10
CXCR3
CCL5
IFNG
C1QB
SPON2
IL7R
C1QA
CSF1R
CLEC5A
MARCO
CYBB
MS4A6A
LILRA2
CTLA4
SLAMF1
SIGLEC1
SLAMF8
LILRB4
IL21R
SLC15A3
FGL2
S100A5.indicator
high S100A5
low S100A5
Effector_gene
CD8_T_cell
Dendritic_cell
Macrophage
NK_cell
Th1_cell
Matrix
1
0.5
0
−0.5
−1
D
ADORA2A
BTLA
C10orf54
CD200
CD200R1
CD274
CD276
CD80
CD86
CEACAM1
CTLA4
HAVCR2
KIR3DL1
LAG3
LAIR1
LGALS3
PDCD1
PVR
PVRL2
VTCN1
S100A5
−1 −0.8 −0.6 −0.4 −0.2 0 0.2 0.4 0.6 0.8 1
E
CCL5
CD27
CD274
CD276
CD8A
CMKLR1
CXCL9
CXCR6
HLA-DQA1
HLA-DRB1
HLA-E
LAG3
NKG7
PDCD1LG2
PSMB10
STAT1
S100A5
1
0.8
0.6
0.4
0.2
0
−0.2
−0.4
−0.6
−0.8
−1

GSE69795

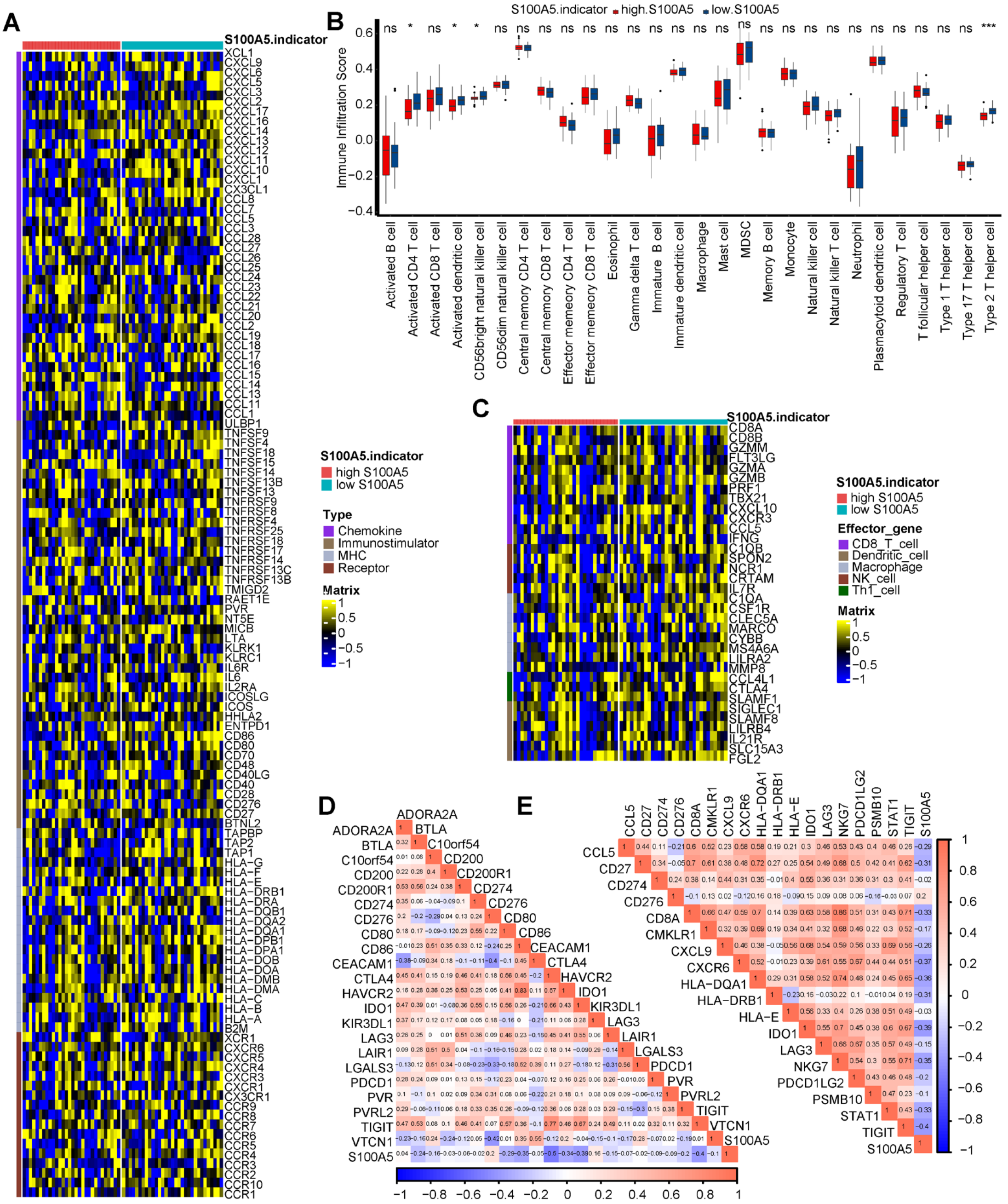
A
S100A5.indicator
XCL1
CXCL9
CXCL6
CXCL5
CXCL3
CXCL2
CXCL17
CXCL16
CXCL14
CXCL13
CXCL12
CXCL11
CXCL10
CXCL1
CX3CL1
CCL8
CCL7
CCL5
CCL3
CCL28
CCL27
CCL26
CCL25
CCL24
CCL23
CCL22
CCL21
CCL20
CCL2
CCL19
CCL18
CCL17
CCL16
CCL15
CCL14
CCL13
CCL11
CCL1
ULBP1
TNFSF9
TNFSF4
TNFSF18
TNFSF15
TNFSF14
TNFSF13B
TNFSF13
TNFRSF9
TNFRSF8
TNFRSF4
TNFRSF25
TNFRSF18
TNFRSF17
TNFRSF14
TNFRSF13C
TNFRSF13B
TMIGD2
RAET1E
PVR
NT5E
MICB
LTA
KLRK1
KLRC1
IL6R
IL6
IL2RA
ICOSLG
ICOS
HHLA2
ENTPD1
CD86
CD80
CD70
CD48
CD40LG
CD40
CD28
CD276
CD27
BTNL2
TAPBP
TAP2
TAP1
HLA-G
HLA-F
HLA-E
HLA-DRB1
HLA-DRA
HLA-DQB1
HLA-DQA2
HLA-DQA1
HLA-DPB1
HLA-DPA1
HLA-DOB
HLA-DOA
HLA-DMB
HLA-DMA
HLA-C
HLA-B
HLA-A
B2M
XCR1
CXCR6
CXCR5
CXCR4
CXCR3
CXCR1
CX3CR1
CCR9
CCR8
CCR7
CCR6
CCR5
CCR4
CCR3
CCR2
CCR10
CCR1
S100A5.indicator
high S100A5
low S100A5
Type
Chemokine
Immunostimulator
MHC
Receptor
Matrix
1
0.5
0
−0.5
−1
B
S100A5.indicator
high.S100A5
low.S100A5
Immune Infiltration Score
0.6
0.4
0.2
0.0
−0.2
−0.4
ns * ns * * ns ns ns ns ns ns ns ns ns ns ns ns ns ns ns ns ns ns ns ns ns ns ***
Activated B cell
Activated CD4 T cell
Activated CD8 T cell
Activated dendritic cell
CD56bright natural killer cell
CD56dim natural killer cell
Central memory CD4 T cell
Central memory CD8 T cell
Effector memeory CD4 T cell
Effector memeory CD8 T cell
Eosinophil
Gamma delta T cell
Immature B cell
Immature dendritic cell
Macrophage
Mast cell
MDSC
Memory B cell
Monocyte
Natural killer cell
Natural killer T cell
Neutrophil
Plasmacytoid dendritic cell
Regulatory T cell
T follicular helper cell
Type 1 T helper cell
Type 17 T helper cell
Type 2 T helper cell
C
S100A5.indicator
CD8A
CD8B
GZMM
FLT3LG
GZMA
GZMB
PRF1
TBX21
CXCL10
CXCR3
CCL5
IFNG
C1QB
SPON2
NCR1
CRTAM
IL7R
C1QA
CSF1R
CLEC5A
MARCO
CYBB
MS4A6A
LILRA2
MMP8
CCL4L1
CTLA4
SLAMF1
SIGLEC1
SLAMF8
LILRB4
IL21R
SLC15A3
FGL2
S100A5.indicator
high S100A5
low S100A5
Effector_gene
CD8_T_cell
Dendritic_cell
Macrophage
NK_cell
Th1_cell
Matrix
1
0.5
0
−0.5
−1
D
ADORA2A
BTLA
C10orf54
CD200
CD200R1
CD274
CD276
CD80
CD86
CEACAM1
CTLA4
HAVCR2
IDO1
KIR3DL1
LAG3
LAIR1
LGALS3
PDCD1
PVR
PVRL2
TIGIT
VTCN1
S100A5
−1 −0.8 −0.6 −0.4 −0.2 0 0.2 0.4 0.6 0.8 1
E
CCL5
CD27
CD274
CD276
CD8A
CMKLR1
CXCL9
CXCR6
HLA−DQA1
HLA−DRB1
HLA−E
IDO1
LAG3
NKG7
PDCD1LG2
PSMB10
STAT1
TIGIT
S100A5
1
0.8
0.6
0.4
0.2
0
−0.2
−0.4
−0.6
−0.8
−1

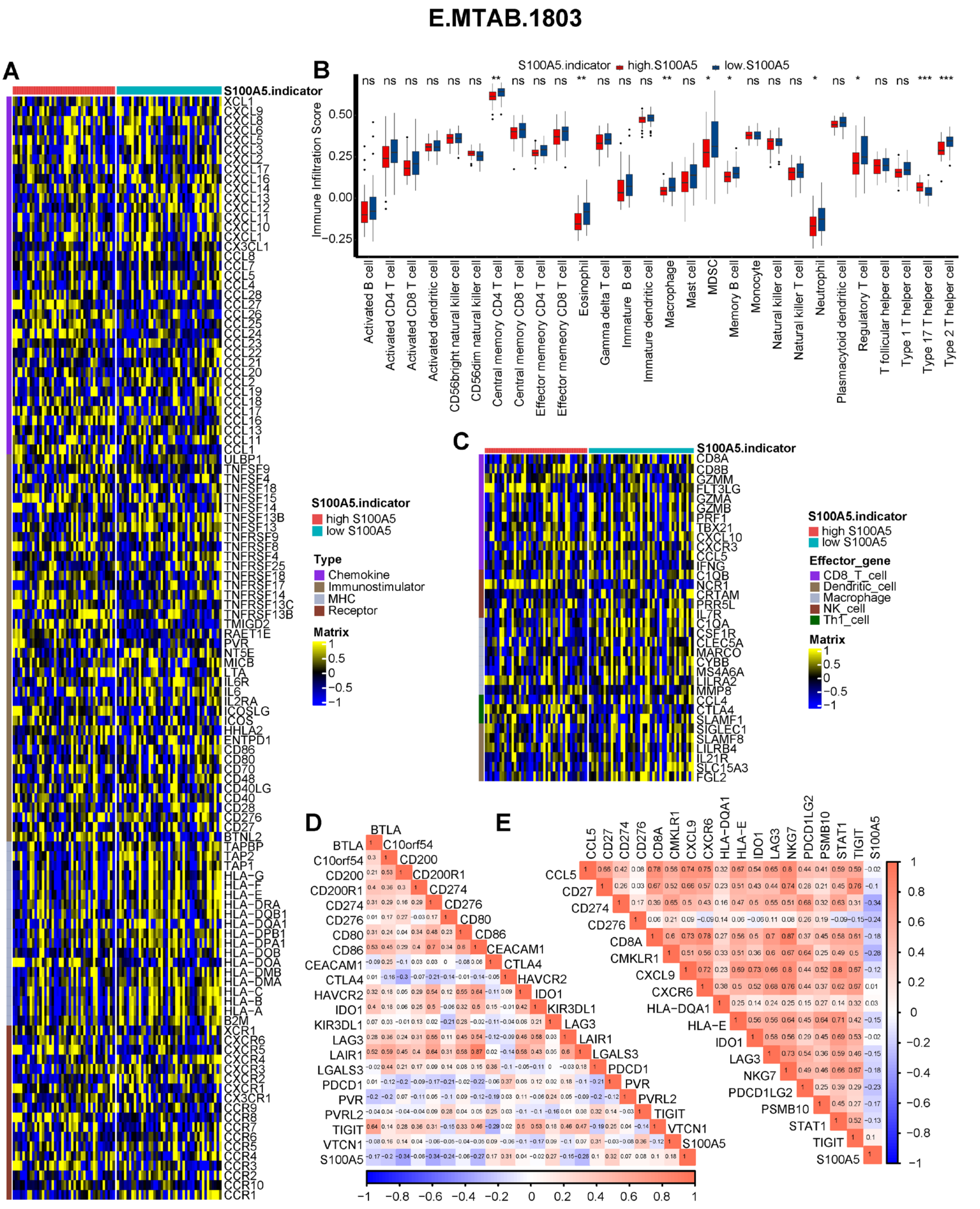

Figure S4-8. Validation of the role of S100A5 in predicting microenvironment infiltration characterization in GSE120736, GSE31684, GSE32894, GSE69795 and E-MTAB-1803 databases. (A) Different expression patterns of immunostimulators, MHC, receptor, and chemokine between high and low S100A5 group; Yellow represents higher expressed genes, while blue represents lower expressed genes. (B) The histogram of tumor-infiltrating immune cells using ssGSEA algorithm between high and low S100A5 groups; Red, high S100A5 group; Blue, low S100A5 group. (C) Different expression patterns of effector genes of CD8 T cells, dendritic cells,

macrophage cells, NK cells and Th1 cells between high and low S100A5 groups; Yellow represents higher expressed genes, while blue represents lower expressed genes. (D-E) Correlation between S100A5 and immune checkpoint inhibitor genes and T cell-inflamed genes respectively; Positive correlation was marked in orange, while negative correlation was marked in blue. *$P < 0.05$; **$P < 0.01$; ***$P < 0.001$; ****$P < 0.0001$; ns, not statistically significant.

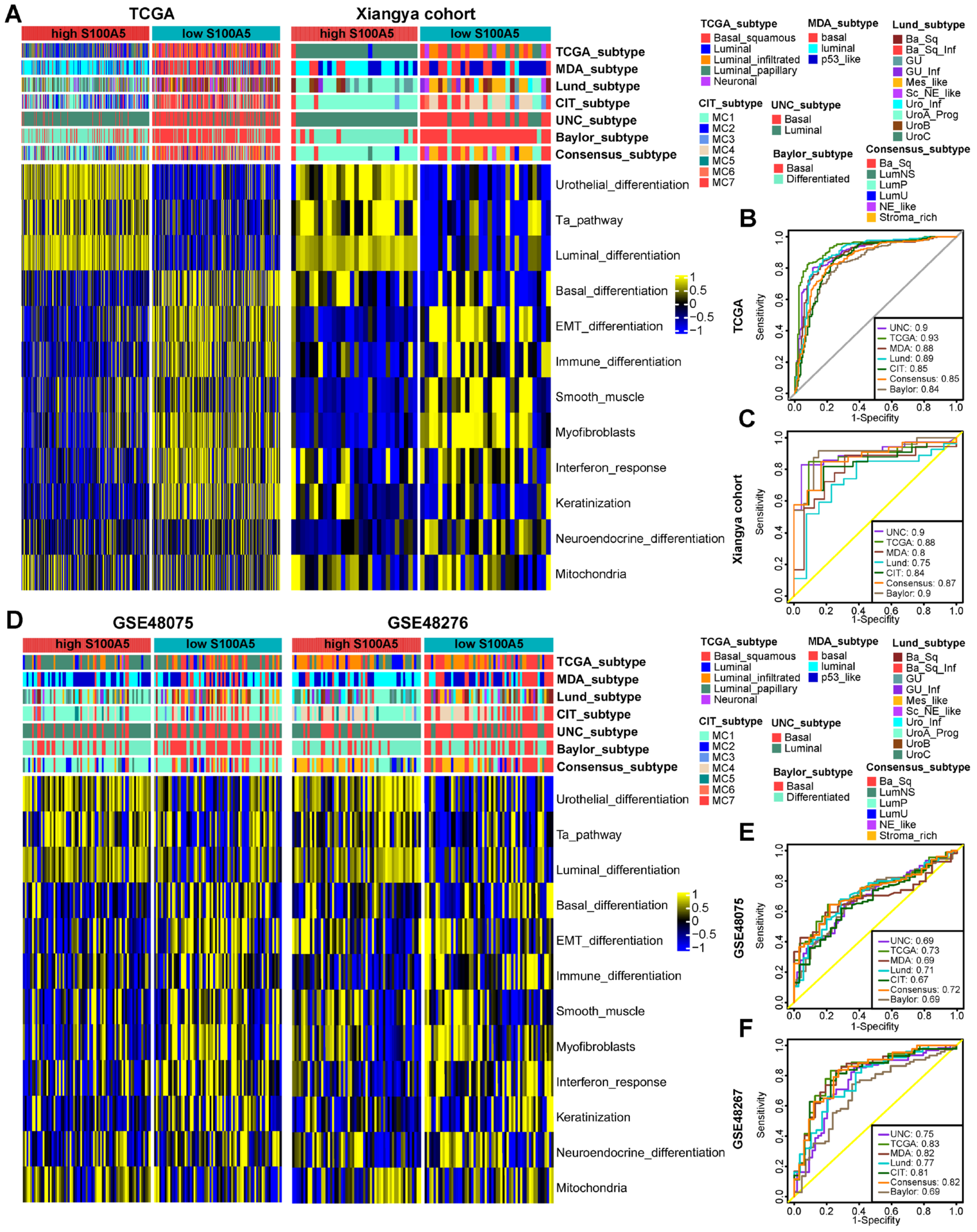

Figure S9. The role of S100A5 in predicting molecular subtype of BLCA. (A) Correlation between S100A5, molecular subtypes of BLCA, and twelve bladder cancer

associated signatures in TCGA-BLCA cohort and Xiangya cohort respectively. Activated pathways were marked in yellow, while the inhibited pathways were marked in blue. (B-C) Predictive accuracy of S100A5 for molecular subtypes of seven algorithms in TCGA-BLCA cohort (B) and Xiangya cohort (C) respectively. (D) Correlation between S100A5, molecular subtypes of BLCA, and twelve bladder cancer associated signatures in GSE48075 and GSE48276 respectively. (E-F) Predictive accuracy of S100A5 for molecular subtypes of seven algorithms in GSE48075 (E) and GSE48276 (F) respectively.

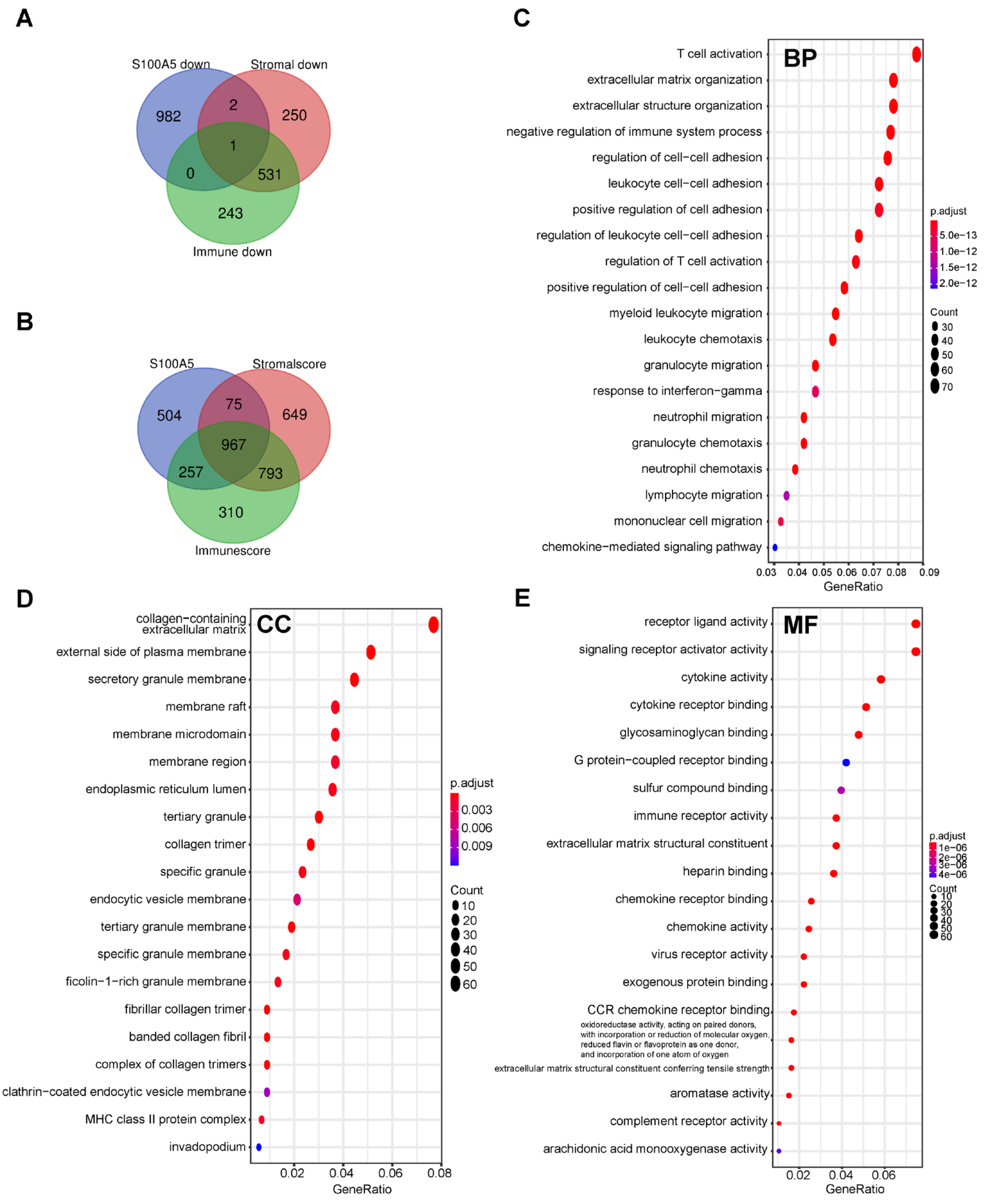

Figure S10. Common differentially expressed genes (DEGs) and GO enrichment results between S100A5 expression, stromal and immune scores. (A) Common DEGs between low S100A5 group, low stromal score group and low immune score group. (B) Common DEGs between S100A5 expression, stromal score and immune score. (C-E) Biological process (BP) (C), cellular component (CC) (D) and molecular function (MF) (E) of common DEGs.

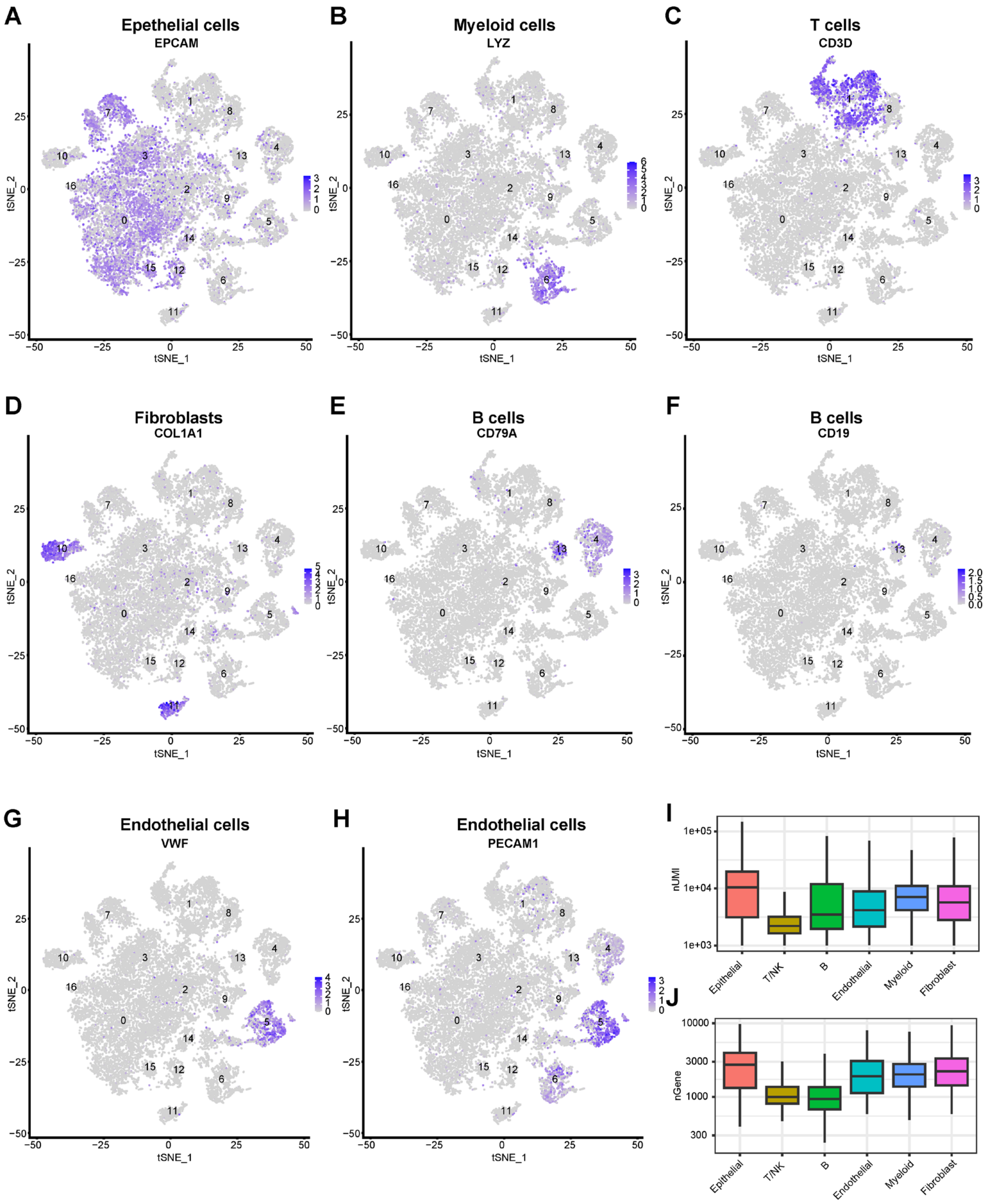

Figure S11. Expression of marker genes for identified cell types in Xiangya scRNA-seq. (A-H) Six major clusters were identified: epithelial cells (EPCAM), myeloid cells (LYZ), T cells (CD3D), fibroblasts (COL1A1), B cells (CD79A and CD19), and endothelial cells (PECAM1 and VWF). (I-J) Boxplots of nUMIs (I) and nGenes (J) among epithelial cells and immune cells.

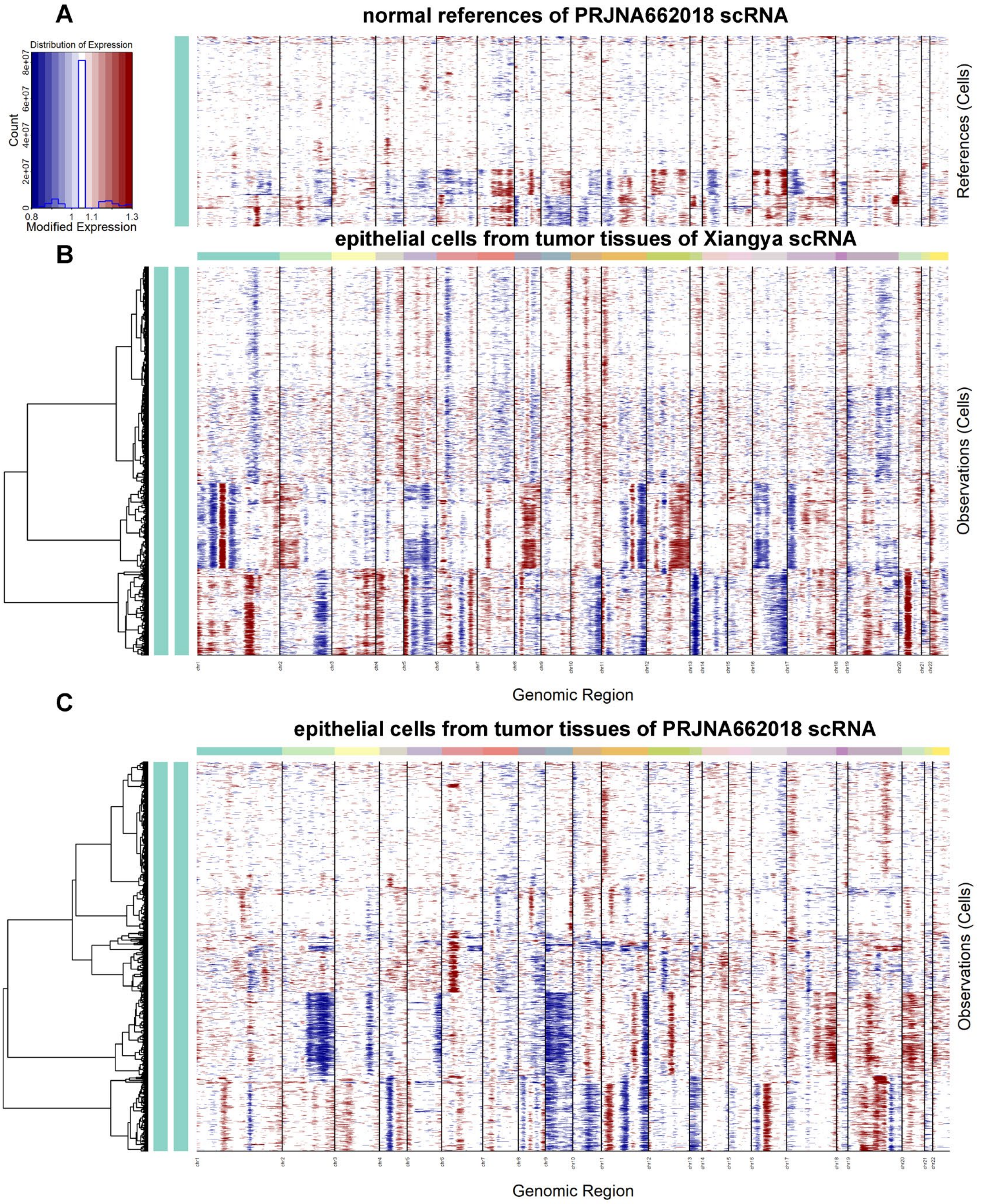

Figure S12. Copy number variations (CNVs) performed on each cell using InferCNV R package in Xiangya scRNA-seq and PRJNA662018 scRNA-seq.

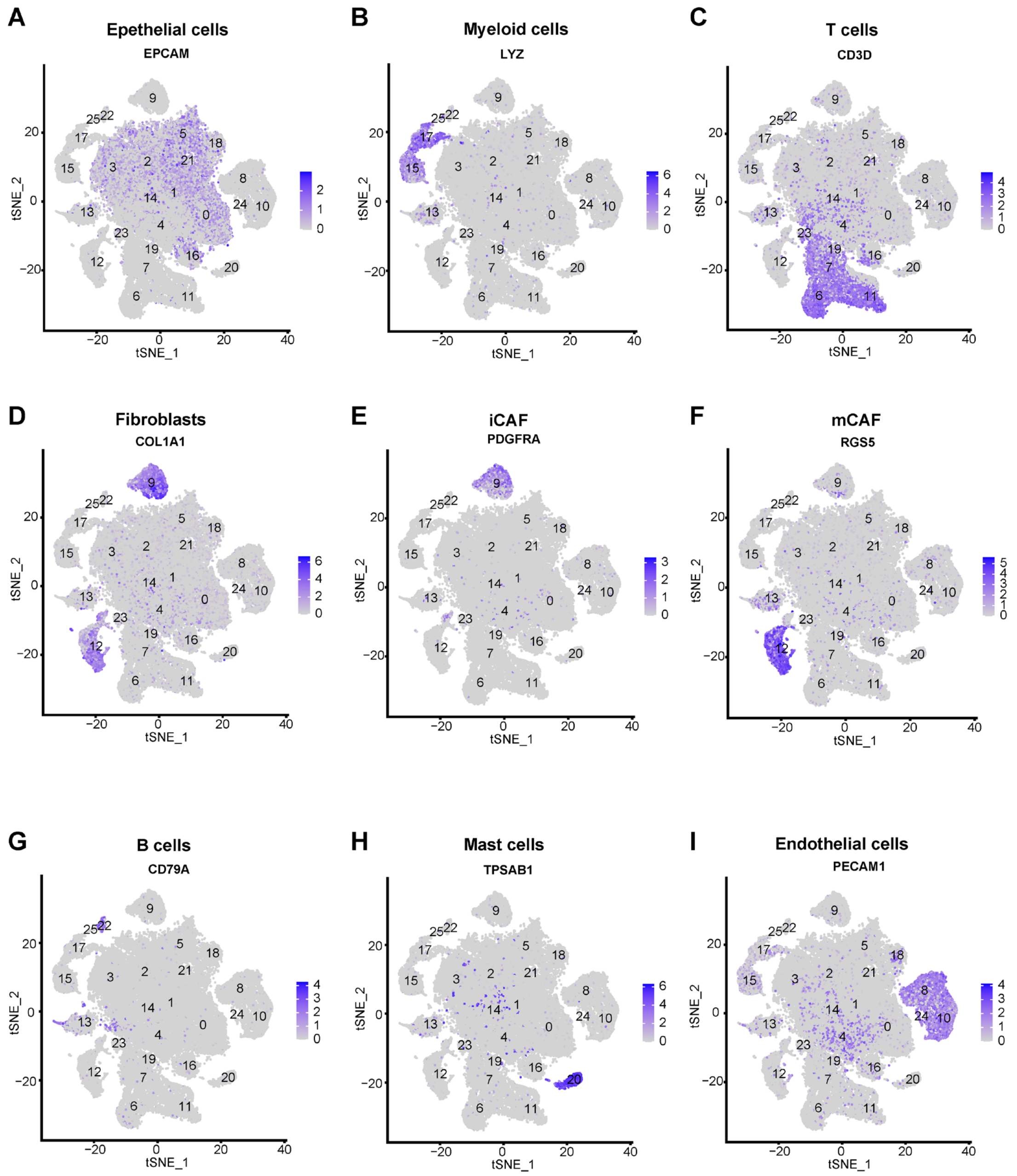

Figure S13. Expression of marker genes for identified cell types in PRJNA662018 scRNA cohort. Eight major clusters were identified: epithelial cells (EPCAM), myeloid cells (LYZ), T cells (CD3D), iCAF (COL1A1 and PDGFRA), mCAF (COL1A1 and RGS5), B cells (CD79A), Mast cells (TPSAB1) and endothelial cells (PECAM1).

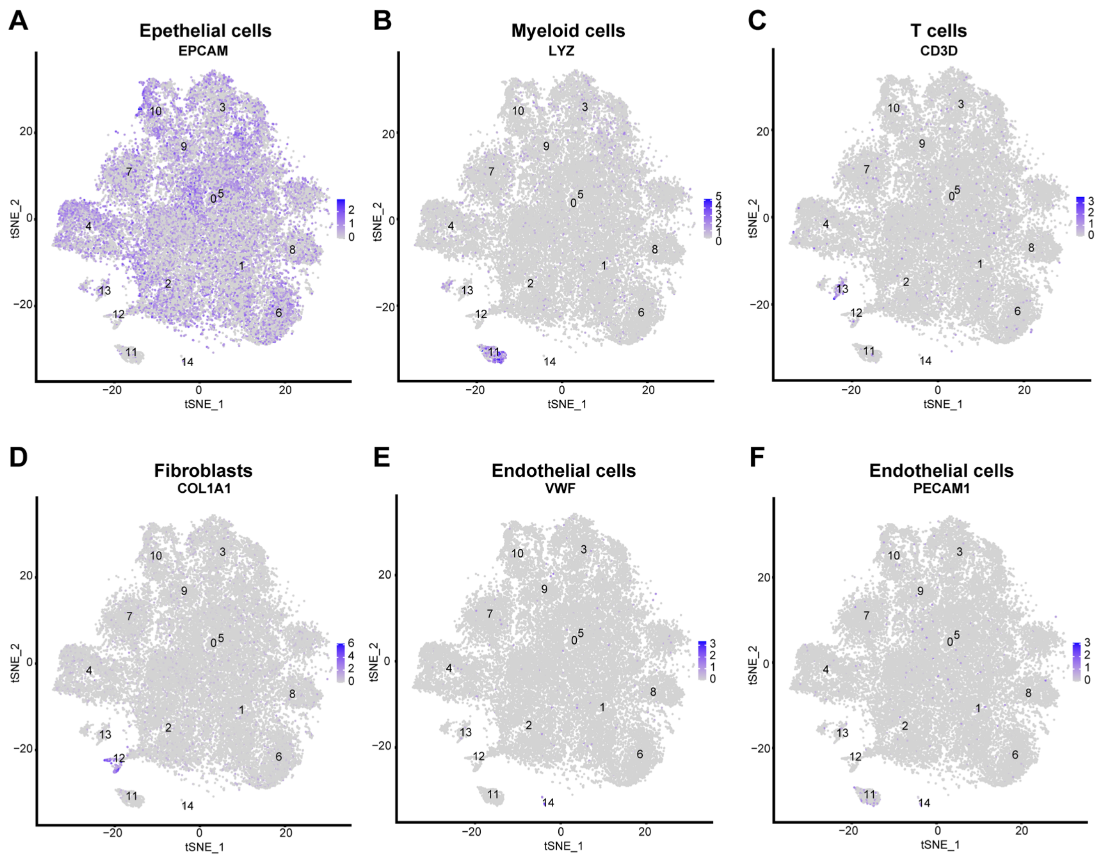

Figure S14. Expression of marker genes for identified cell types in GSE135337. Five major clusters were identified: epithelial cells (EPCAM), myeloid cells (LYZ), T cells (CD3D), fibroblasts (COL1A1), and endothelial cells (PECAM1 and VWF).

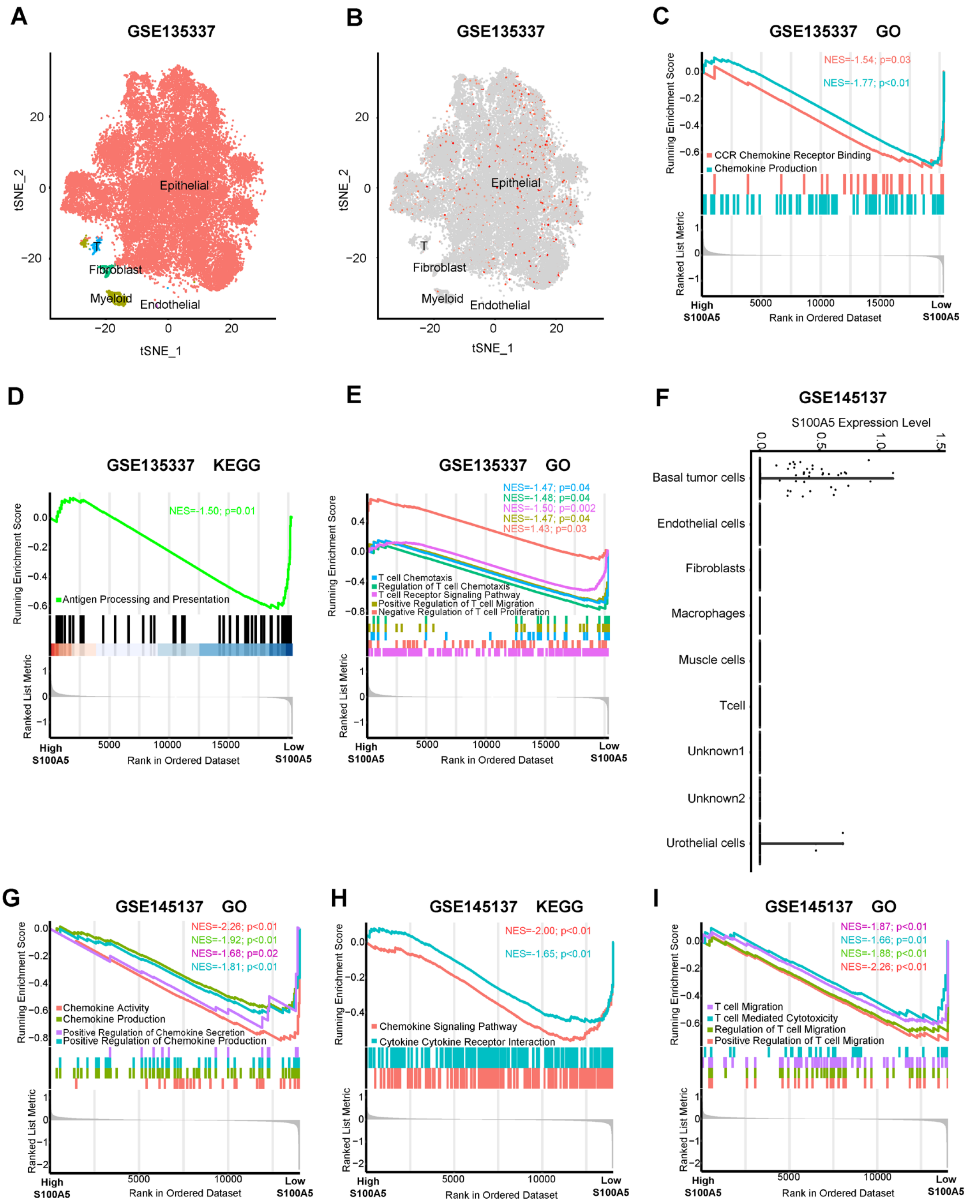

Figure S15. Validation of the specifical expression of S100A5 in malignant epithelial cells and its potential effects in the TME using GSE135337 and GSE145137. (A) tSNE plot of all the single cells from GSE135337, with each color representing different major cell types. (B) tSNE plot shows the expression patterns of S100A5 on the single cell level in GSE135337. (C-D) GSEA shows enrichment of cytokines and chemokines secretion related GO pathways (C) and antigen processing and presentation related KEGG pathways (D) between different S100A5 expression groups in malignant epithelial cells of GSE135337 cohort. (E) GSEA shows enrichment

of T cell proliferation and activation related GO pathways between different S100A5 expression groups in malignant epithelial cells of GSE135337 cohort. (F) Violin plot shows expression level of S100A5 across different cell types in GSE145137. (G-H) GSEA shows enrichment of cytokines and chemokines secretion related GO pathways (G) and KEGG pathways (H) in basal tumor cells of GSE145137. (I) GSEA shows enrichment of T cell proliferation and activation related GO pathways in basal tumor cells of GSE145137.

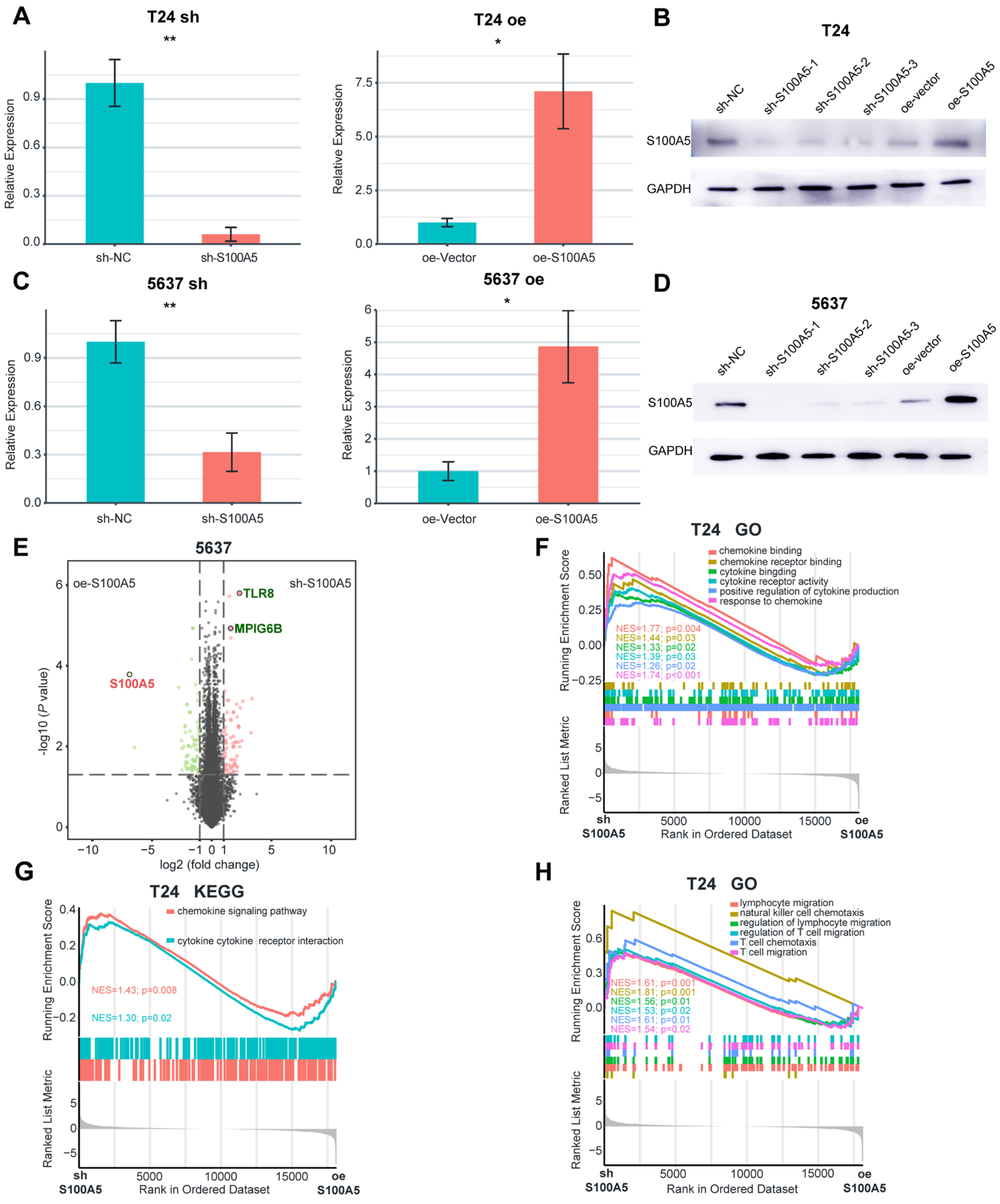

Figure S16. Successful construction of S100A5 OE and S100A5 KD cell lines and

RNA-seq data of 5637 and T24 cell lines. (A) Expression level of S100A5 in T24 cell line using RT-PCR. (B) Protein level of S100A5 in T24 cell line using Western Plot. (C). Expression level of S100A5 in 5637 cell line using RT-PCR. (D) Protein level of S100A5 in 5637 cell line using Western Plot. (E) Volcano plots between knockdown and overexpression of S100A5 in 5637cell lines. (F-G) GSEA shows enrichment of cytokines and chemokines secretion related GO pathways (F) and KEGG pathways (G) between the knockdown and overexpression of S100A5 in T24 cells. (H) GSEA shows the enrichment of T cell proliferation and activation-related GO pathways between the knockdown and overexpression of S100A5 in T24 cell lines.

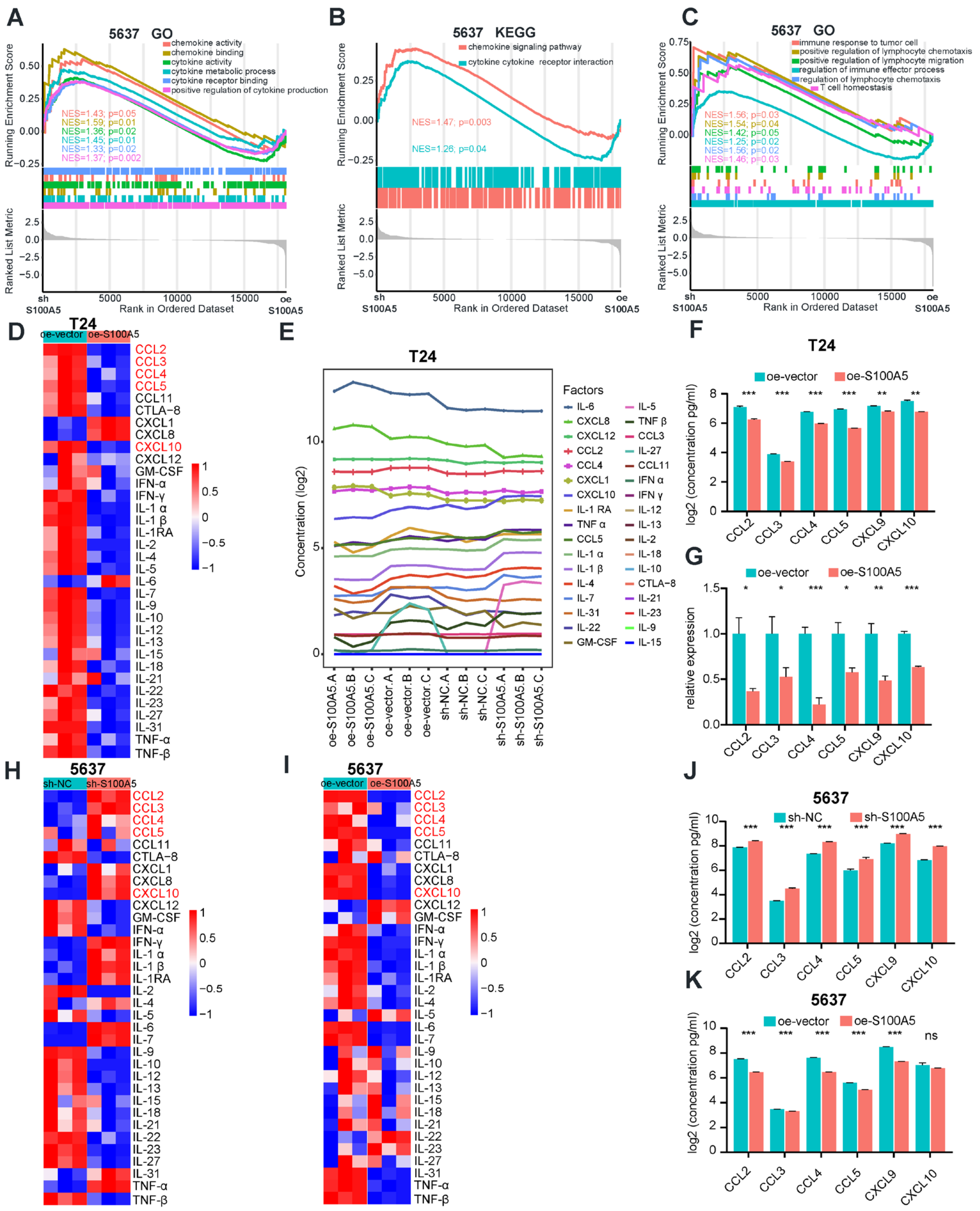

Figure S17. Other in vitro experiments. (A-B) GSEA shows enrichment of cytokines and chemokines secretion related GO pathways (A) and KEGG pathways (B) between knockdown and overexpression of S100A5 in 5637 cell lines. (C) GSEA shows enrichment of T cell proliferation and activation related GO pathways between knockdown and overexpression of S100A5 in 5637 cell lines. (D) Heatmap of multiple cytokines and chemokines detected by ProcartaPlex multiple immunoassays between overexpression of S100A5 and negative control groups in T24 cell line culture supernatants. (E) Line chart shows the concentrations of cytokines and chemokines

detected by ProcartaPlex multiple immunoassays among different T24 cell lines. (F-G) CCL2, CCL3, CCL4, CCL5, CXCL9 and CXCL10 levels detected using Elisa (F) and RT-PCR (G) respectively between overexpression of S100A5 and negative control groups in T24 cell line. (H-I) Heatmap of multiple cytokines and chemokines detected by ProcartaPlex multiple immunoassays between knockdown of S100A5 and negative control groups (H), overexpression of S100A5 and negative control groups (I) in 5637 cell culture supernatants. Red represents higher secretion, while blue represents lower secretion. (J-K) CCL2, CCL3, CCL4, CCL5, CXCL9 and CXCL10 levels detected using Elisa between knockdown of S100A5 and negative control groups (J), overexpression of S100A5 and negative control groups (K) in 5637 cell line. *$P < 0.05$; **$P < 0.01$; ***$P < 0.001$; ****$P < 0.0001$; ns, not statistically significant.

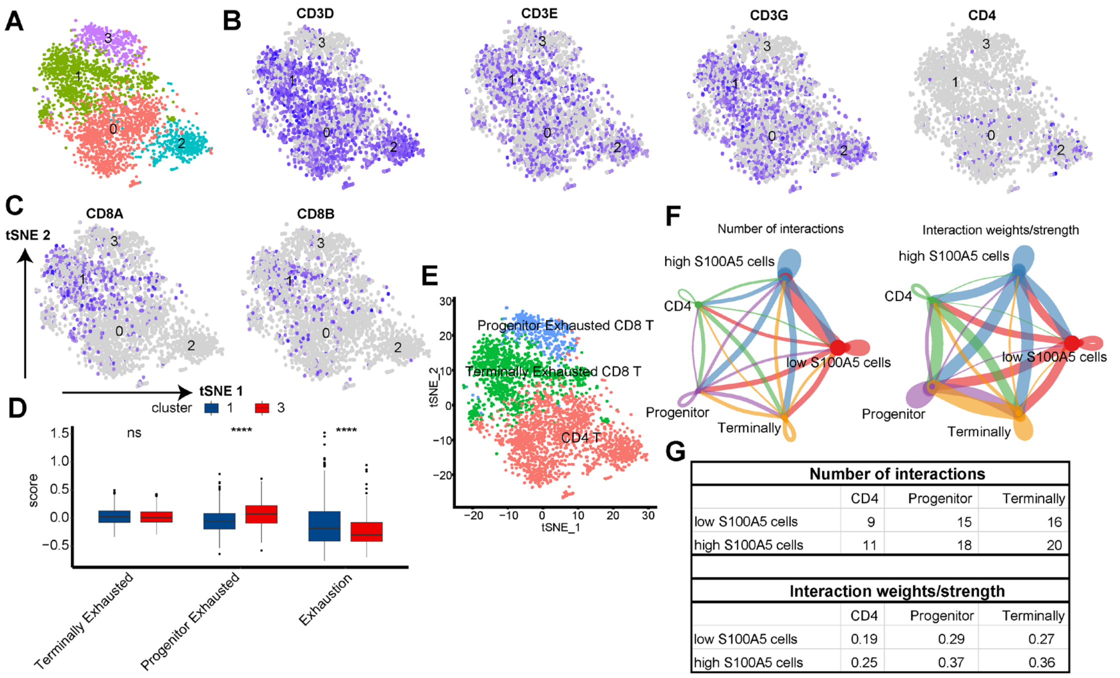

Number of interactions	CD4	Progenitor	Terminally
low S100A5 cells	9	15	16
high S100A5 cells	11	18	20

Interaction weights/strength	CD4	Progenitor	Terminally
low S100A5 cells	0.19	0.29	0.27
high S100A5 cells	0.25	0.37	0.36

Figure S18. Cell chat analysis in Xiangya scRNA-seq cohort. (A) Subclustering of T cells on the tSNE plots of the Xiangya scRNA-seq. (B-C) Marker genes for T cell subgroups. (D) Boxplots of T cell exhaustion signatures between $CD8^+$ T cell subgroups. (E) tSNE plots of the marked T cell subgroups. (F) Network plots showing the number of interactions and weights/strength among high/low S100A5 expression malignant epithelial cells and T cell subgroups. (G) Number of interactions and weights/strength among high/low S100A5 expression malignant epithelial cells and T cell subgroups.

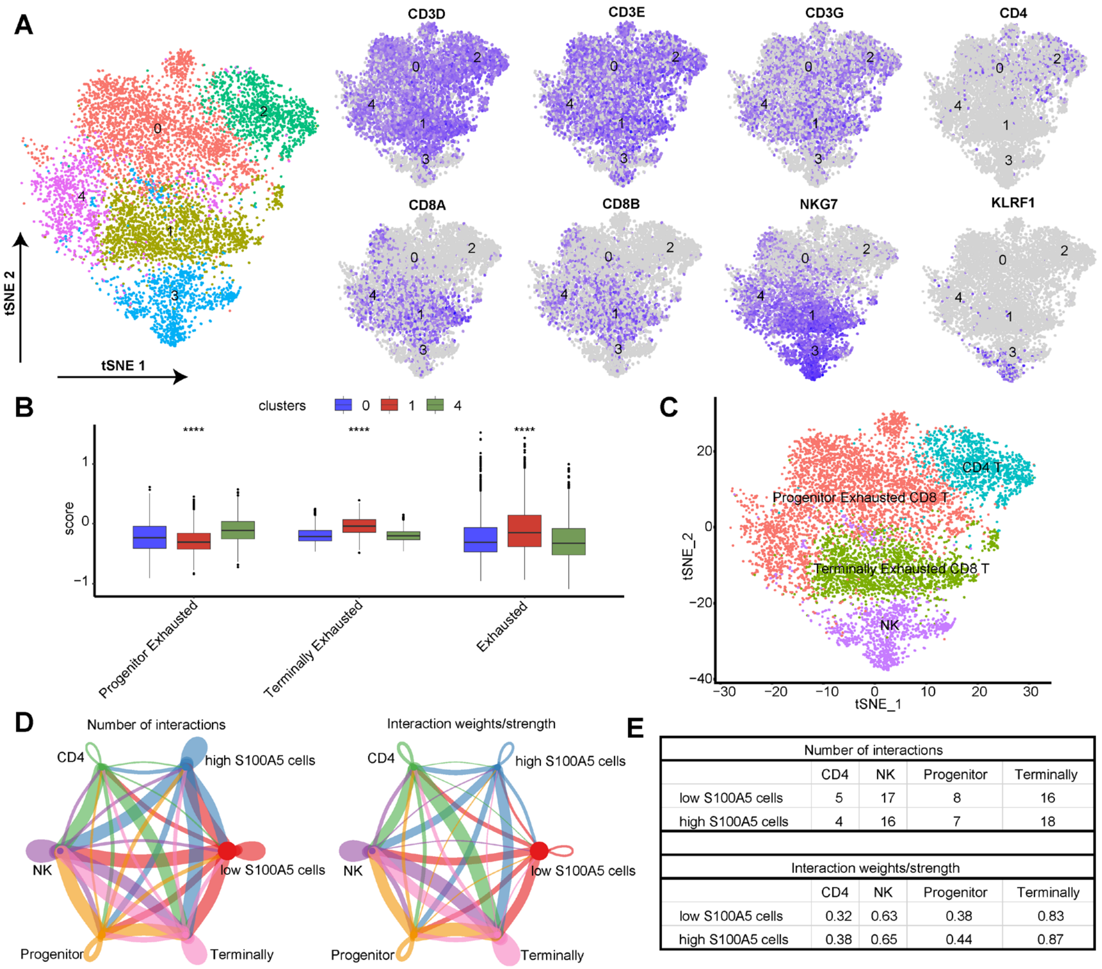

Number of interactions	CD4	NK	Progenitor	Terminally
low S100A5 cells	5	17	8	16
high S100A5 cells	4	16	7	18

Interaction weights/strength	CD4	NK	Progenitor	Terminally
low S100A5 cells	0.32	0.63	0.38	0.83
high S100A5 cells	0.38	0.65	0.44	0.87

Figure S19. Cell chat analysis in PRJNA662018 scRNA-seq cohort. (A) Subclustering of T cells and marker genes on the tSNE plots of the PRJNA662018 scRNA-seq. (B) Boxplots of T cell exhaustion signatures between $CD8^{+}$ T cell subgroups. (C) tSNE plots of the marked T cell subgroups. (D) Network plots showing the number of interactions and weights/strength among high/low S100A5 expression malignant epithelial cells and T cell subgroups. (E) Number of interactions and weights/strength among high/low S100A5 expression malignant epithelial cells and T cell subgroups.

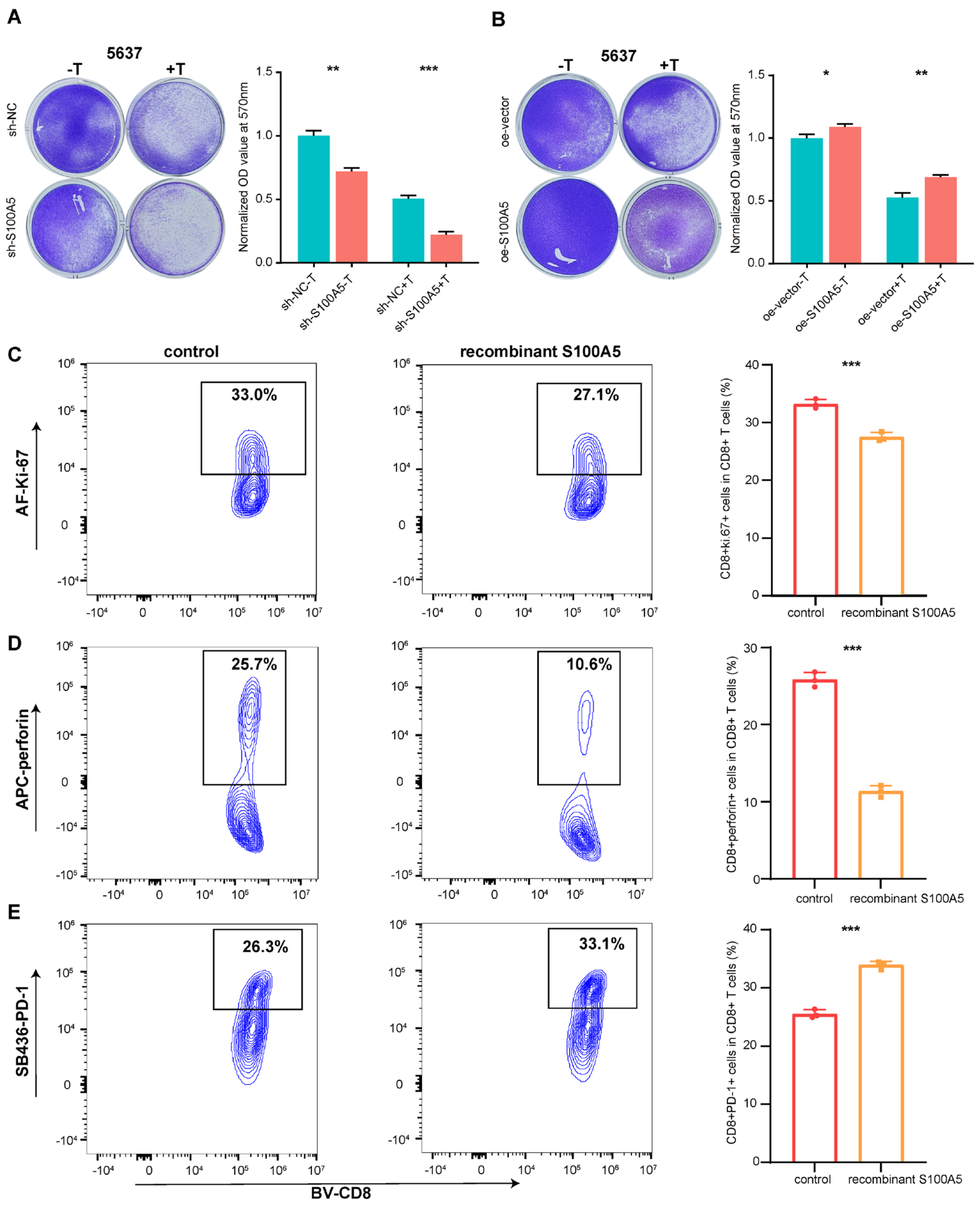

Figure S20. Recombinant S100A5 inhibited the function of CD8^{+} T cells. (A) Representative images and histogram plots of T cell-mediated tumor cell-killing assay between S100A5 knocking down and negative control groups in 5637 cell line. The OD values were normalized by the mean value in sh-NC group without T cell coculture. (B) Representative images and histogram plots of T cell-mediated tumor cell-killing assay between S100A5 overexpression and negative control groups in 5637 cell line. The OD values were normalized by the mean value in oe-vector group without T cell coculture. (C-E) Flow cytometry analysis shows the different Ki-67 (C), perforin (D) and PD-1

(E) expression on effector T cells after culture with Recombinant S100A5 or isotype control. *$P < 0.05$; **$P < 0.01$; ***$P < 0.001$.

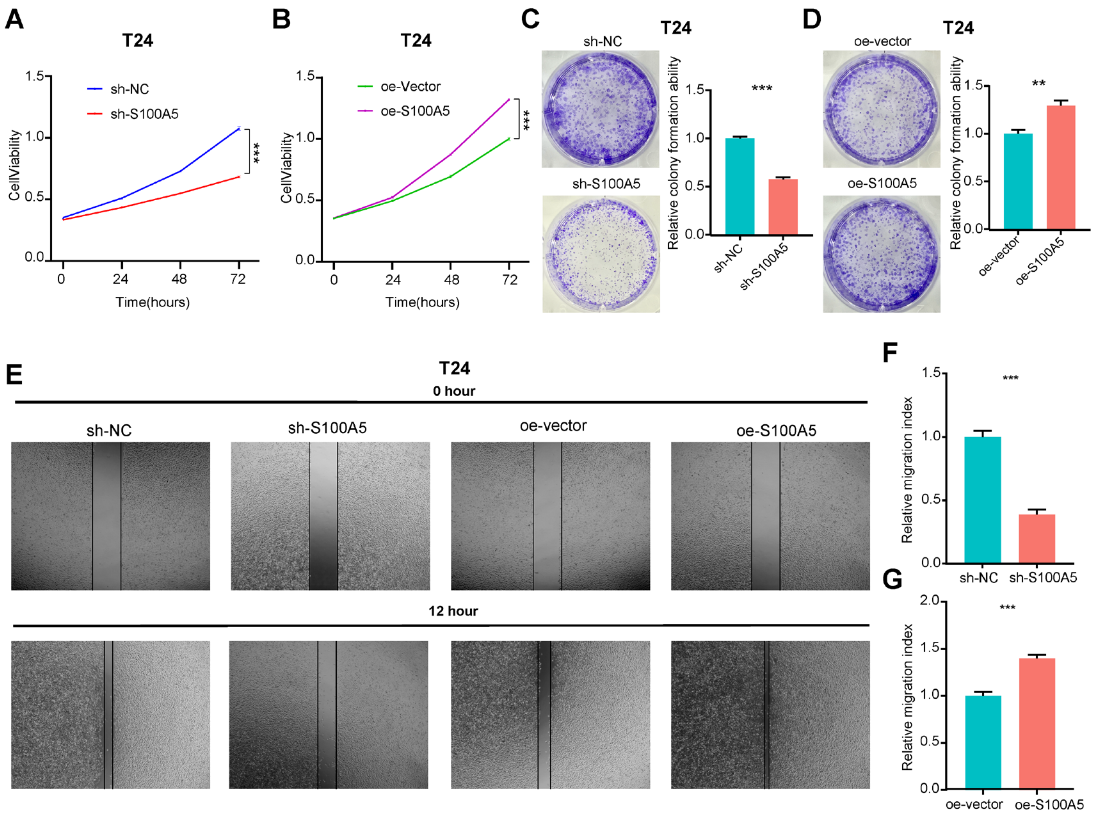

Figure S21. S100A5 acts as an oncogene and thus promotes tumor proliferation and invasion. (A-B) MTT cell viability assays of S100A5 knockdown (A) and overexpression (B) groups compared with their negative control in the T24 cell line. (C) Colony formation assays between S100A5 knockdown and negative control group in the T24 cell line. (D) Colony formation assays between S100A5 overexpression and negative control group in the T24 cell line. (E) Representative images of wound healing assays among S100A5 knockdown, overexpression, and their negative controls in the T24 cell line. (F-G) Histogram plots of the relative migration index between S100A5 knockdown and negative control (F) and S100A5 overexpression and negative control (G). Values were normalized to those of negative control groups. **$P < 0.01$; ***$P < 0.001$.

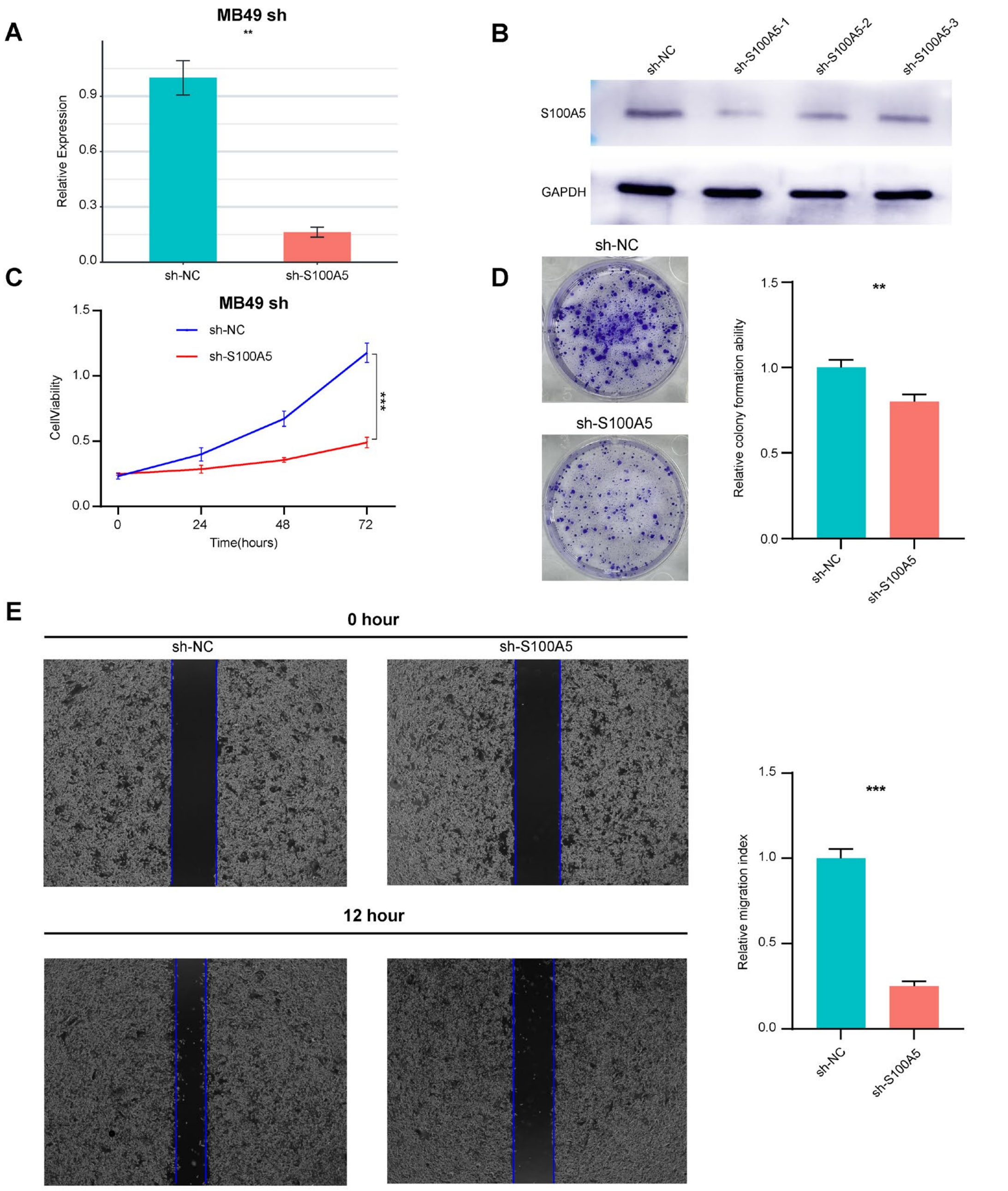

Figure S22. Cancer phenotype experiments in MB49 cell line. (A) Expression level of S100A5 in MB49 cell line using RT-PCR. (B) Protein level of S100A5 in MB49 cell line using Western Plot. (C) MTT cell viability assay between S100A5 knocking down and negative control groups in MB49 cell lines. (D) Colony formation assays between S100A5 knocking down and negative control group in MB49 cell line. (E) Representative images of wound healing assays and histogram plots of relative migration index between S100A5 knocking down and negative control groups in MB49 cell line. $**P < 0.01$; $***P < 0.001$.

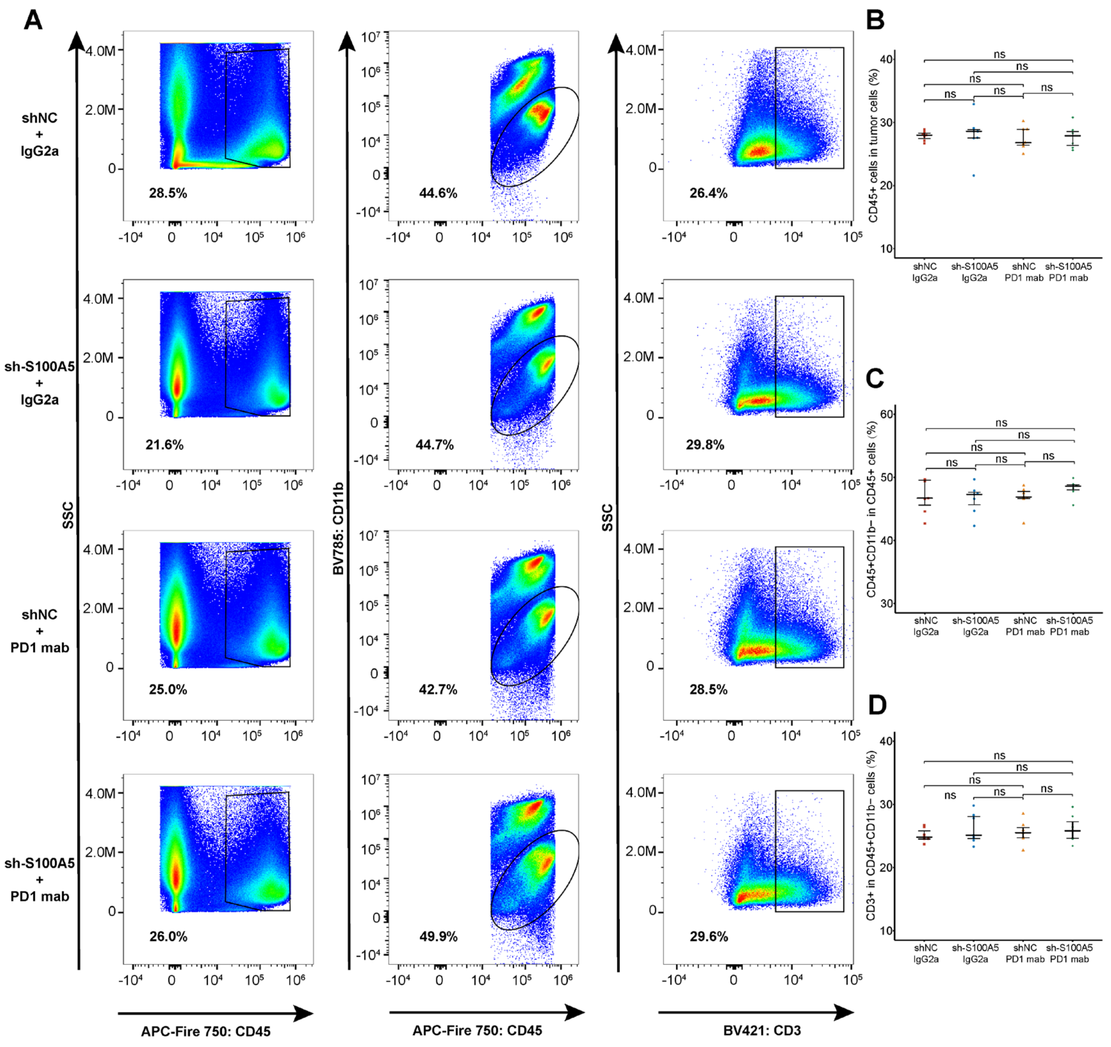

Figure S23. Other flow cytometry analysis for in vivo experiment. (A) Representative contour plots show the proportion of leukocytes ($CD45^{+}$) in tumor cells, lymphoid cells ($CD45^{+}CD11b^{-}$) in leukocytes and T cells ($CD3^{+}$) in lymphoid cells respectively. (B-D) Quantification results of flow cytometry analysis for proportion of leukocytes ($CD45^{+}$) in tumor cells (B), lymphoid cells ($CD45^{+}CD11b^{-}$) in leukocytes (C) and T cells ($CD3^{+}$) in lymphoid cells (D) respectively.

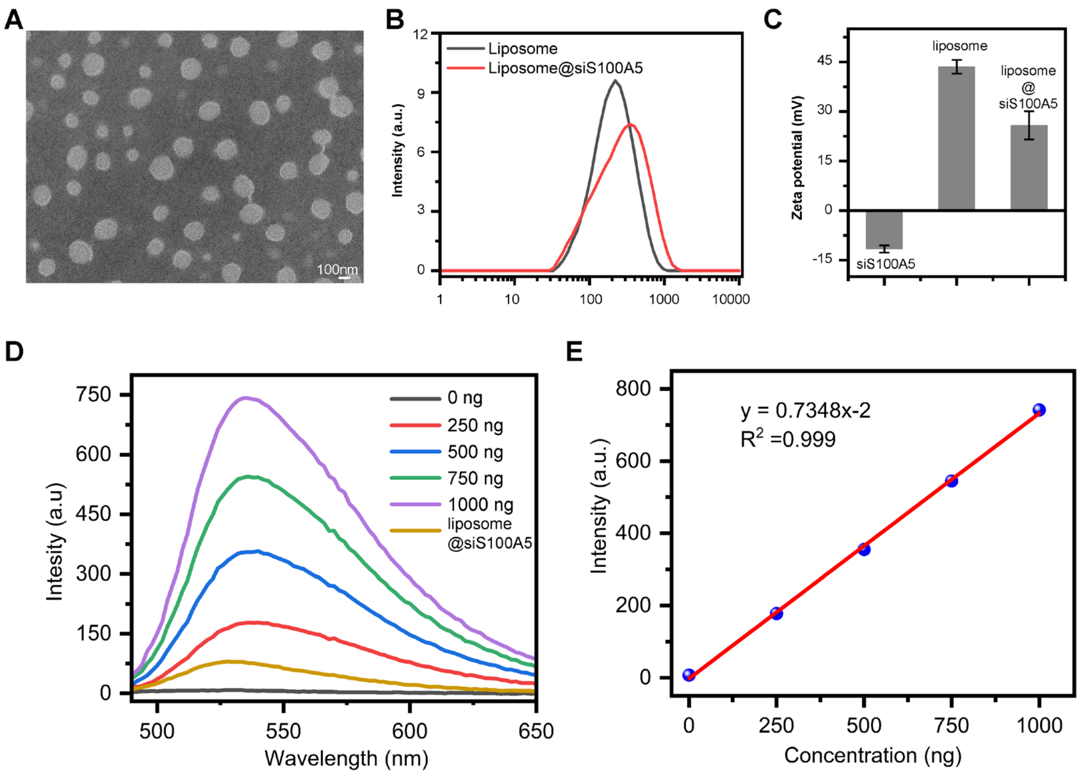

Figure S24. Successful synthesis and characterization of liposome@siS100A5. (A) TEM images of liposome@siS100A5. (B) The DLS of the liposome@siS100A5 and liposome. (C) Zeta potential of siS100A5, liposome and liposome@siS100A5. (D-E) Standard curve shows the relationship between fluorescence intensity and siS100A5 concentration.

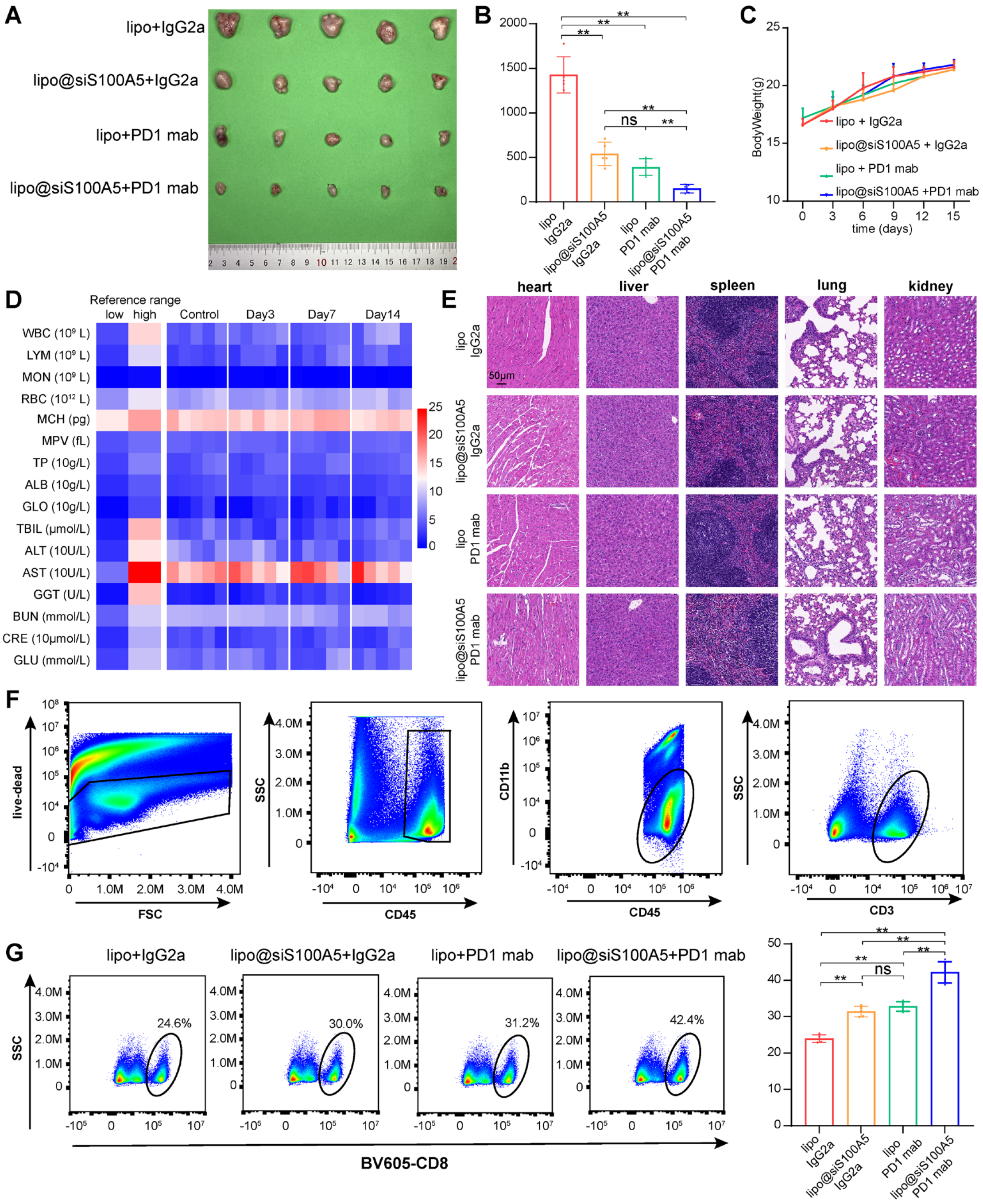

Figure S25. Targeting S100A5 enhanced the efficacy of anti-PD-1 treatment and CD8$^+$ T cells recruitment. (A) Tumor images among different experimental groups after mice sacrifice. (B) Histogram plot of tumor volumes among different experimental groups after mice sacrifice. (C) Scatter diagram plot of body weights among different groups during experimental procedure. (D) Heatmap plot of the mouse whole blood and blood biochemistry indexes. WBC: white blood cell; LYM: lymphocyte; MON: monocyte; RBC: red blood cell; MCH: mean corpuscular hemoglobin; MPV: mean platelet volume; TP: total protein; ALB: albumin; GLO: globulin; TBIL: total bilirubin;

ALT: alanine aminotransferase; AST: aspartate transaminase; GGT: γ-glutamyl transpeptidase; BUN: blood urea nitrogen; CRE: creatinine; GLU: glucose. (E) H&E staining of heart, liver, spleen, lung, and kidney among different experimental groups. (F) Gate strategy of flow cytometry analysis. (G) Representative contour plots and the proportion of $CD8^+$ T cells in T cells. ns, not statistically significant; $^{**}P < 0.01$.

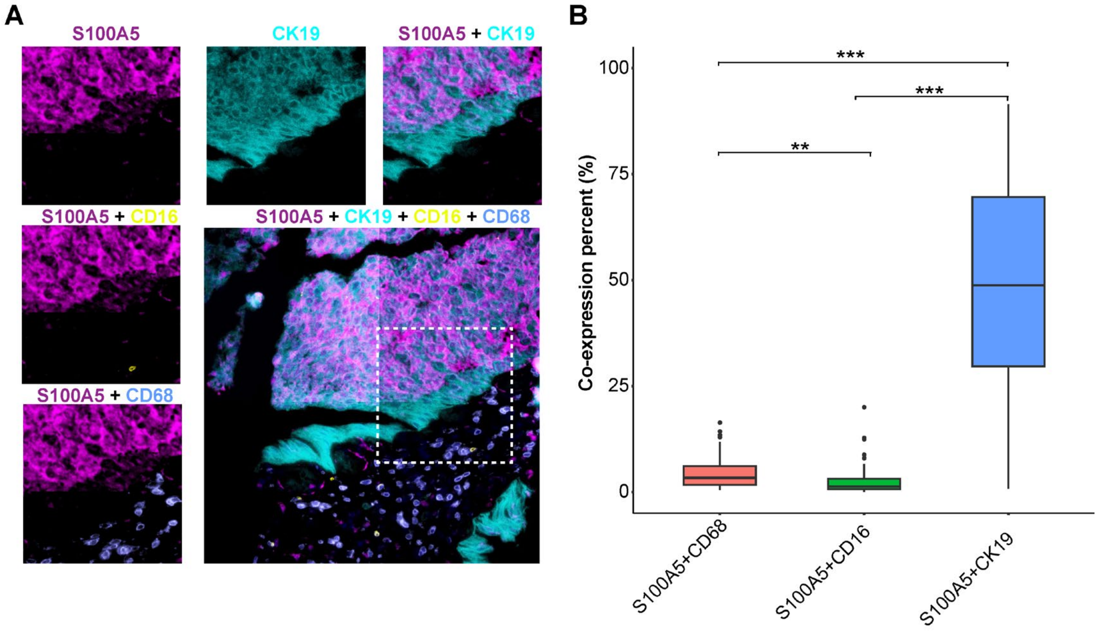

Figure S26. (A) Co-expression of S100A5 on malignant cell, NK cell and macrophage: S100A5 (purple), CK19 (azure), CD16 (yellow), CD68 (light blue). (B) The histograms of different $S100A5^+CD68^+$, $S100A5^+CD16^+$ and $S100A5^+CK19^+$ percent cells among the whole TMA.
